# Identification of novel risk loci for restless legs syndrome in genome-wide association studies in individuals of European ancestry: a meta-analysis

**DOI:** 10.1016/S1474-4422(17)30327-7

**Published:** 2017-11

**Authors:** Barbara Schormair, Chen Zhao, Steven Bell, Erik Tilch, Aaro V Salminen, Benno Pütz, Yves Dauvilliers, Ambra Stefani, Birgit Högl, Werner Poewe, David Kemlink, Karel Sonka, Cornelius G Bachmann, Walter Paulus, Claudia Trenkwalder, Wolfgang H Oertel, Magdolna Hornyak, Maris Teder-Laving, Andres Metspalu, Georgios M Hadjigeorgiou, Olli Polo, Ingo Fietze, Owen A Ross, Zbigniew Wszolek, Adam S Butterworth, Nicole Soranzo, Willem H Ouwehand, David J Roberts, John Danesh, Richard P Allen, Christopher J Earley, William G Ondo, Lan Xiong, Jacques Montplaisir, Ziv Gan-Or, Markus Perola, Pavel Vodicka, Christian Dina, Andre Franke, Lukas Tittmann, Alexandre F R Stewart, Svati H Shah, Christian Gieger, Annette Peters, Guy A Rouleau, Klaus Berger, Konrad Oexle, Emanuele Di Angelantonio, David A Hinds, Bertram Müller-Myhsok, Juliane Winkelmann, 23andMe Research Team, DESIR study group, B Balkau, P Ducimetière, E Eschwège, F Rancière, F Alhenc-Gelas, Y Gallois, A Girault, F Fumeron, M Marre, R Roussel, F Bonnet, A Bonnefond, S Cauchi, P Froguel, J Cogneau, C Born, E Caces, M Cailleau, O Lantieri, JG Moreau, F Rakotozafy, J Tichet, S Vol, Michelle Agee, Babak Alipanahi, Adam Auton, Robert K Bell, Katarzyna Bryc, Sarah L Elson, Pierre Fontanillas, Nicholas A Furlotte, David A Hinds, Bethann S Hromatka, Karen E Huber, Aaron Kleinman, Nadia K Litterman, Matthew H McIntyre, Joanna L Mountain, Carrie AM Northover, Steven J Pitts, J Fah Sathirapongsasuti, Olga V Sazonova, Janie F Shelton, Suyash Shringarpure, Chao Tian, Joyce Y Tung, Vladimir Vacic, Catherine H Wilson

**Affiliations:** a Institute of Neurogenomics, Helmholtz Zentrum München, German Research Centre for Environmental Health, Neuherberg, Germany; b Institute of Epidemiology II, Helmholtz Zentrum München, German Research Centre for Environmental Health, Neuherberg, Germany; c Research Unit of Molecular Epidemiology, Helmholtz Zentrum München, German Research Centre for Environmental Health, Neuherberg, Germany; d National Institute for Health Research Blood and Transplant Unit in Donor Health and Genomics at the University of Cambridge, Strangeways Research Laboratory, University of Cambridge, Cambridge, UK; e MRC/BHF Cardiovascular Epidemiology Unit, Department of Public Health and Primary Care, Strangeways Research Laboratory, University of Cambridge, Cambridge, UK; f Department of Haematology, University of Cambridge, Cambridge Biomedical Campus, Cambridge, UK; g NHS Blood and Transplant, Oxford, UK; h NHS Blood and Transplant, Cambridge, UK; i National Institute for Health Research Cambridge Biomedical Research Centre, Cambridge, UK; j Max Planck Institute of Psychiatry, Munich, Germany; k Sleep-Wake Disorders Centre, Department of Neurology, Hôpital Gui-de-Chauliac, INSERM U1061, CHU Montpellier, France; l Department of Neurology, Medical University of Innsbruck, Innsbruck, Austria; m Department of Neurology and Centre of Clinical Neuroscience, First Faculty of Medicine and General University Hospital in Prague, Charles University, Prague, Czech Republic; n Department of Neurology, Paracelsus Klinik, Osnabrueck, Germany; o Department of Clinical Neurophysiology, University Medical Centre, Georg August University Göttingen, Göttingen, Germany; p Clinic for Neurosurgery, University Medical Centre, Georg August University Göttingen, Göttingen, Germany; q Paracelsus-Elena Hospital, Centre of Parkinsonism and Movement Disorders, Kassel, Germany; r Department of Neurology, Philipps University Marburg, Marburg, Germany; s Department of Neurology, University of Ulm, Ulm, Germany; t Neuropsychiatry Centre Erding/München, Erding, Germany; u Estonian Genome Centre, University of Tartu and Estonian Biocentre, Tartu, Estonia; v Laboratory of Neurogenetics, Department of Neurology, Faculty of Medicine, University of Thessaly, University Hospital of Larissa, Biopolis, Larissa, Greece; w Unesta Research Centre, Tampere, Finland; x Department of Pulmonary Diseases, Tampere University Hospital, Tampere, Finland; y Department of Cardiology and Angiology, Centre of Sleep Medicine, Charité-Universitätsmedizin Berlin, Berlin, Germany; z Department of Neuroscience, Mayo Clinic, Jacksonville, FL, USA; aa Department of Neurology, Mayo Clinic, Jacksonville, FL, USA; ab British Heart Foundation Centre of Excellence, Division of Cardiovascular Medicine, Addenbrooke's Hospital, Cambridge, UK; ac Department of Human Genetics, Wellcome Trust Sanger Institute, Wellcome Trust Genome Campus, Hinxton, UK; ad Radcliffe Department of Medicine, BRC Haematology Theme and NHS Blood and Transplant, John Radcliffe Hospital, Headington, Oxford, UK; ae Department of Haematology and BRC Haematology Theme, Churchill Hospital, Oxford, UK; af Center for Restless Legs Study, Department of Neurology, Johns Hopkins University, Baltimore, MD, USA; ag Department of Neurology, Methodist Neurological Institute, Houston, TX, USA; ah Laboratoire de Neurogénétique, Centre de Recherche, Institut Universitaire en Santé Mentale de Montréal, Montréal, QC, Canada; ai Département de Psychiatrie, Université de Montréal, Montréal, QC, Canada; aj Department of Neurology and Neurosurgery, McGill University, Montréal, QC, Canada; ak Department of Human Genetics, McGill University, Montréal, QC, Canada; al Hôpital du Sacré-Coeur de Montréal, 67120, Center for Advanced Research in Sleep Medicine, Montréal, QC, Canada; am Montreal Neurological Institute, McGill University, Montréal, QC, Canada; an Department of Health, National Institute for Health and Welfare, Helsinki, Finland; ao Department of Molecular Biology of Cancer, Institute of Experimental Medicine, Academy of Science of Czech Republic, Prague, Czech Republic; ap Institute of Molecular Medicine FIMM, University of Helsinki, Helsinki, Finland; aq Biomedical Centre, Faculty of Medicine in Pilsen, Charles University in Prague, Pilsen, Czech Republic; ar Inserm UMR1087, CNRS UMR 6291, Institut du Thorax, Nantes, France; as Centre Hospitalier Universitaire (CHU) Nantes, Université de Nantes, France; at Institute of Clinical Molecular Biology, Kiel University, Kiel, Germany; au PopGen Biobank and Institute of Epidemiology, Christian Albrechts University Kiel, Kiel, Germany; av John and Jennifer Ruddy Canadian Cardiovascular Genetics Centre, University of Ottawa Heart Institute, Ottawa, ON, Canada; aw Department of Medicine, Duke University School of Medicine, Durham, NC, USA; ax Duke Clinical Research Institute, Duke University School of Medicine, Durham, NC, USA; ay German Centre for Diabetes Research (DZD), Neuherberg, Germany; az German Centre for Cardiovascular Disease Research (DZHK), Berlin, Germany; ba Institute of Epidemiology and Social Medicine, University of Münster, Münster, Germany; bb Munich Cluster for Systems Neurology (SyNergy), Munich, Germany; bc Institute of Translational Medicine, University of Liverpool, Liverpool, UK; bd Institute of Human Genetics, Technische Universität München, Munich, Germany; be Neurologische Klinik und Poliklinik, Klinikum rechts der Isar der Technischen Universität München, Munich, Germany; bf 23andMe, Mountain View, CA, USA

## Abstract

**Background:**

Restless legs syndrome is a prevalent chronic neurological disorder with potentially severe mental and physical health consequences. Clearer understanding of the underlying pathophysiology is needed to improve treatment options. We did a meta-analysis of genome-wide association studies (GWASs) to identify potential molecular targets.

**Methods:**

In the discovery stage, we combined three GWAS datasets (EU-RLS GENE, INTERVAL, and 23andMe) with diagnosis data collected from 2003 to 2017, in face-to-face interviews or via questionnaires, and involving 15 126 cases and 95 725 controls of European ancestry. We identified common variants by fixed-effect inverse-variance meta-analysis. Significant genome-wide signals (p≤5 × 10^−8^) were tested for replication in an independent GWAS of 30 770 cases and 286 913 controls, followed by a joint analysis of the discovery and replication stages. We did gene annotation, pathway, and gene-set-enrichment analyses and studied the genetic correlations between restless legs syndrome and traits of interest.

**Findings:**

We identified and replicated 13 new risk loci for restless legs syndrome and confirmed the previously identified six risk loci. *MEIS1* was confirmed as the strongest genetic risk factor for restless legs syndrome (odds ratio 1·92, 95% CI 1·85–1·99). Gene prioritisation, enrichment, and genetic correlation analyses showed that identified pathways were related to neurodevelopment and highlighted genes linked to axon guidance (associated with *SEMA6D*), synapse formation (*NTNG1*), and neuronal specification (*HOXB cluster* family and *MYT1*).

**Interpretation:**

Identification of new candidate genes and associated pathways will inform future functional research. Advances in understanding of the molecular mechanisms that underlie restless legs syndrome could lead to new treatment options. We focused on common variants; thus, additional studies are needed to dissect the roles of rare and structural variations.

**Funding:**

Deutsche Forschungsgemeinschaft, Helmholtz Zentrum München–Deutsches Forschungszentrum für Gesundheit und Umwelt, National Research Institutions, NHS Blood and Transplant, National Institute for Health Research, British Heart Foundation, European Commission, European Research Council, National Institutes of Health, National Institute of Neurological Disorders and Stroke, NIH Research Cambridge Biomedical Research Centre, and UK Medical Research Council.

## Introduction

Despite the prevalence of restless legs syndrome being up to 10% in populations of European ancestry, its genetic basis and underlying pathophysiology remain unclear. The restless legs syndrome phenotype is an unusual composite of sensory and motor symptoms that present with distinct circadian rhythmicity. The symptoms worsen or are only present in the evening or at night and markedly lessen in the early morning. Patients feel an overwhelming urge to move, often in conjunction with unpleasant sensations, usually in the legs. Rest and inactivity provoke the symptoms, whereas movement and other external stimuli lead to temporary relief.^1^ Due to the chronic progressive nature of the disorder, it has long-lasting effects on patients' mental and physical health. People with restless legs syndrome have substantially impaired sleep, reduced overall quality of life, and increased risk of depression, anxiety disorders, hypertension, and, possibly, cardiovascular disease.^2^ Around 2–3% of the general population have severe restless legs syndrome, and most need chronic treatment with dopaminergics, α_2_δ ligands, or even opioids.^1^ However, long-term use of dopaminergics can lead to severe side-effects, including the worsening of symptoms (augmentation). Hence, there is an urgent need for alternative treatments.

**Research in context**

**Evidence before this study**

We searched PubMed for articles published up to July, 2017, with combinations of the search term “restless legs AND (genomewide OR genome-wide OR GWAS)”, without restrictions on language of publication. This search yielded 42 original articles, including reports on genetic linkage analyses and genome-wide association studies (GWASs). Although linkage results on restless legs syndrome have not been reproducible, GWASs so far have revealed six risk loci, with the strongest signal being in MEIS1, a member of the three aminoacid loop extension homeobox gene class. Subsequent studies have suggested altered embryonic development of the striatum is also important in the aetiology of restless legs syndrome. Nevertheless, the pathogenesis needs further elucidation. Changes in dopaminergic signalling and brain iron deficiency seem to be involved and are targeted by approved drugs. Other suggested causes include peripheral hypoxia and unknown metabolic factors related to uraemia and pregnancy. The current recommended treatment for restless legs syndrome, although effective, can lead to serious adverse effects, including worsening of symptoms, thus alternatives are needed.

**Added value of this study**

We did a meta-analysis of three GWAS datasets, which yielded a total sample size more than one order of magnitude larger than any previously published restless legs syndrome GWAS. We discovered 13 new risk loci for restless legs syndrome, taking the total from six to 19. Assessment of these candidate genes will enable more granular dissection of the pathogenesis of restless legs syndrome, which could improve determination of shared genetic architecture with other neurological phenotypes and the prospects of developing novel and more effective treatment options. Our results strongly support the link to neurogenesis, changes in neuronal circuit formation, synaptogenesis, and axonal guidance, thereby strengthening the concept of restless legs syndrome as a neurodevelopmental disorder. The novel risk loci include the genes *CRBN*, which encodes cereblon, and *MEIS2*, which encodes its physiological substrate homeobox protein Meis2. As thalidomide targets this interaction, this drug could be a candidate for the treatment of restless legs syndrome in patients beyond reproductive age.

**Implications of all the available evidence**

The genes and associated pathways identified provide a much-needed basis for future investigation of restless legs syndrome, informing which pathophysiological and pharmacological concepts to examine in laboratory and clinical trials. Our findings further suggest investigating the role of neurodevelopmental processes in restless legs syndrome and the mechanism of the interaction between cereblon and homeobox protein Meis2 in the context of assessing the repurposing of thalidomide.

The likelihood of developing symptoms of restless legs syndrome is strongly affected by genetic factors. Family and twin studies have estimated that heritability is 50–60%.^3^ Individual genetic risk variants and their putative target genes, however, were identified only when genome-wide association studies (GWASs) became feasible. Six risk loci have so far been identified in this way,^4^, ^5^, ^6^, ^7^ and have notably shaped research by uncovering potential new pathophysiological mechanisms. Genes in these loci provided reliable entry points for functional investigations at the molecular level and for animal studies.^8^, ^9^, ^10^, ^11^

Given these genetically driven advances, we did a meta-analysis of GWAS on restless legs syndrome in people of European ancestry, followed by replication in an independent dataset. We hypothesised that this approach would enable us to detect novel risk loci and pathways associated with restless legs syndrome that would provide further insights into the molecular mechanisms underlying the disorder, and yield possible novel therapeutic targets or avenues for the repurposing of existing drugs.

## Methods

### Study populations and phenotype definitions

For the discovery meta-analysis, we combined three GWAS datasets. The EU-RLS-GENE consortium GWAS includes cases and population-matched controls recruited in eight European countries, Canada, and the USA. People with restless legs syndrome were recruited in specialist outpatient clinics for movement disorders and in sleep units. Restless legs syndrome was diagnosed in face-to-face interviews by an expert neurologist, based on the International Restless Legs Syndrome Study Group diagnostic criteria.^1^, ^12^ A subset of the samples had been used in previous GWASs for restless legs syndrome (appendix p 10).^4^, ^6^, ^7^

The INTERVAL study^13^, ^14^ included whole-blood donors recruited in England, enrolled between 2012 and 2014. The validated Cambridge-Hopkins Restless Legs Questionnaire^15^ was used to identify patients with restless legs syndrome and to exclude mimicking disorders. Probable and definite cases were combined in one group.

The 23andMe GWAS dataset comprised samples drawn from participants of the customer base of 23andMe (Mountain View, CA, USA), which is a genetic testing company. The restless legs syndrome phenotype was determined with one research question, “Have you ever been diagnosed with restless legs syndrome?”, which had three response options: yes, no, and not sure. We include respondents who answered “yes” as cases and those who answered “no” as controls, and we excluded those who answered “not sure”.

For the replication study, we used the 23andMe customer base to identify a distinct population. We used the same selection methods as for the discovery population.

Participants of all the original studies had provided informed consent, and the study protocols had been approved by the respective ethics committees. Demographics of the study samples are provided in the appendix (p 10).

### Genotyping, quality control, imputation, and statistical analysis

Genotyping, quality-control procedures, imputation, and statistical analysis methods of the individual studies are described in detail in the appendix (pp 4–5, pp 11–12). In brief, all studies did association analyses under an additive model by logistic regression on genotype dosage, adjusted for age, sex, and components from either multidimensional scaling or principal components analysis to correct for population stratification.

### Meta-analysis procedures

For the discovery stage, summary statistics from each individual GWAS dataset were subjected to further quality control with EasyQC, version 8.5, and we did a fixed-effect inverse-variance meta-analysis with METAL, release 2011-03-25 (appendix p 5). To address heterogeneity between studies in the identified association signals, we did a random-effects meta-analysis with METASOFT, version 2.0.1 (appendix p 5). Genomic control was done in each study separately before meta-analysis by calculating the inflation factor λ and adjusting for it.^16^

To define independent genome-wide significant signals (p≤5 × 10^−9^) in the discovery meta-analysis results, we used a two-step procedure (appendix p 5). Briefly, we first assigned variants to clusters with the “clump” command in PLINK software (version 1.90b3.36) based on the association p value and short-range (500 kb) linkage disequilibrium (LD), with each clump defined by one lead single-nucleotide polymorphism (SNP). Second, we tested for statistical independence of the lead SNPs with the stepwise model selection procedure implemented in GCTA, version 1.25.3, taking into account long-range LD (10 Mb) between lead SNPs (appendix p 5). Finally, we did standard conditional analysis in the remaining independent SNP clusters, adjusted for the top-associated lead SNP, to identify secondary independent signals within the cluster.

For the joint analysis of the discovery and replication stages, summary statistics for independent association signals were subjected to fixed-effect inverse-variance and random-effects meta-analysis, as in the discovery stage (appendix p 5).

### Heritability, partitioned heritability, and genetic correlation analysis

Heritability and partitioned heritability were estimated by LD-score regression (with LDSC, version 1.0.0) and using the summary statistics of the discovery meta-analysis without genomic control correction. Partitioned heritability analysis used publicly available partitioned LD scores for 52 functional categories based on the phase 3 dataset of the 1000 Genomes Project, as precomputed by the developers of LDSC. For the genetic correlation analysis, we used the LD Hub database (version 1.2.2), which provides access to summary-level GWAS statistics of more than 200 traits. The analytical procedures and settings are detailed in the appendix (pp 5–6). To estimate the variance explained by genome-wide significant association signals, we calculated Nagelkerke's pseudo-*R*^2^ in the EU-RLS-GENE dataset with tenfold cross validation.

### Genetic risk score analysis

Genetic risk profiles were generated by estimating weighted polygenic risk scores in the EU-RLS-GENE dataset. The weights were based on the effect size estimates of the discovery meta-analysis (fixed-effect model). Polygenic risk scores were calculated with PRSice software, version 1.25 (appendix p 6).

### Biological interpretation of association signals

As a first step, we did a literature-based annotation of protein-coding genes located in the genome-wide significant risk loci, defined based on LD, and in their vicinity (appendix p 6). To identify the biological and cellular pathways underlying the association signals, we applied bioinformatic methods for gene prioritisation and did enrichment analyses for pathways and tissues. In restless legs syndrome, the overall number of risk loci is small, restricting the use of standard tools, such as DEPICT.^17^ Moreover, the enrichment analysis could be hampered by the clinical heterogeneity of restless legs syndrome, its complex phenotype with somatosensory and motor symptoms, and pleiotropic effects of the candidate genes. Therefore, to enable efficient exploration of our data, we developed a new algorithm, called BI-ENRICH, which builds on the concept of biclustering used in gene-expression analysis (appendix pp 6–8). Additionally, we used the DEPICT software (version rel19413) to do gene prioritisation for each locus and to search for gene set and tissue enrichment among these genes (appendix p 8).

### Data sharing

BI-ENRICH code is available on GitHub. GWAS summary statistics will be made available for researchers (appendix p 3).

### Role of the funding source

The funders of the study had no role in study design, data collection, data analysis, data interpretation, or writing of the report. The corresponding author had full access to all the data in the study and had final responsibility for the decision to submit for publication.

## Results

**Figure 1:**
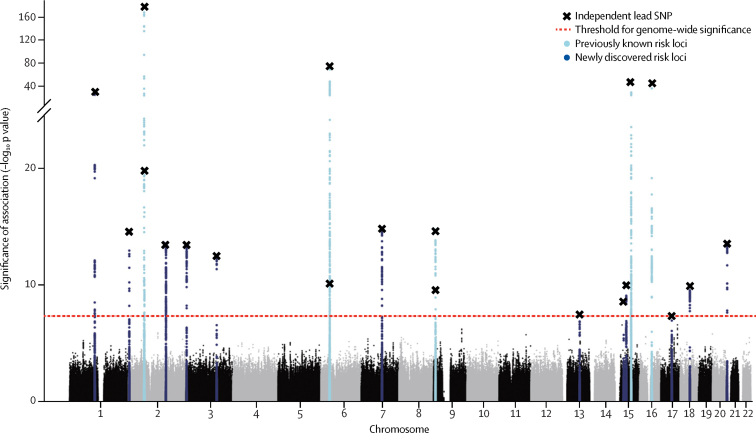
Manhattan plot showing results of the discovery meta-analysis Due to their close proximity, two independent risk loci on chromosome 2 (both previously known) and on chromosome 6 (one previously known, one new) appear as single peaks. Thus, 19 loci (six previously known and 13 new) are represented. SNP=single-nucleotide polymorphism.

The discovery stage association study was done in 15 126 cases and 95 725 controls: 6228 and 10 992 from EU-RLS-GENE; 3065 and 24 923 from INTERVAL; and 5833 and 59 810 from 23andMe. After quality control, 6 864 281 SNPs and indels with minor allele frequency 1% or greater were available for statistical analysis. We identified 20 independent association signals meeting genome-wide significance (figure 1, table 1, appendix p 13). No secondary independent association signals with genome-wide significance were found in these 20 regions by standard conditional analysis. We assigned the 20 independent association signals to 19 independent genomic risk loci (two mapped to the same gene, *PTPRD*, and are <500 kb apart; appendix p 65). Lead SNPs rs1836229 and rs62535767 of these two signals were not correlated (*r*^2^=0·0002, EU-RLS-GENE dataset). Genomic control showed negligible inflation of the median test statistic (λ_1000_=1·004, rescaled to adjust for the large sample size), which LD-score regression revealed was mainly due to polygenicity (intercept 1·018), as is expected for a common complex disorder, rather than being caused by population stratification or other confounders. We observed between-study heterogeneity at some loci, but all signals kept genome-wide significance in the random-effects meta-analysis (appendix p 14).

**Table 1:** Association results for lead single-nucleotide polymorphisms reaching genome-wide significance in the discovery meta-analysis

	**Chromosome**	**Position (bp)**	**Effect allele**	**Other allele**	**Effect-allele frequency**	**Protein-coding gene context**	**Discovery stage meta-analysis**		**Replication stage**		**Joint stage meta-analysis**	
							p value	Odds ratio (95% CI)	p value	Odds ratio (95% CI)	p value	Odds ratio (95% CI)
rs12046503	1	107195339	T	C	0·59	PRMT6*†, *NTNG1*†‡	3·32 × 10^−31^	0·85 (0·84–0·87)	2·03 × 10^−29^	0·90 (0·89–0·92)	3·25 × 10^−63^	0·88 (0·86–0·89)
rs10208712	2	4034446	G	A	0·36	DCDC2C*†	3·78 × 10^−15^	0·90 (0·88–0·91)	7·74 × 10^−19^	0·92 (0·91–0·94)	1·41 × 10^−34^	0·91 (0·90–0·92)
rs113851554	2§	66750564	T	G	0·07	MEIS1*†‡¶	1·1 × 10^−180^	2·16 (2·04–2·29)	4·80 × 10^−236^	1·82 (1·75–1·89)	2·00 × 10^−280^	1·92 (1·85–1·99)
rs1820989	2§	68069890	C	A	0·53	*MEIS1*¶, *C1D*, *APLF†	1·23 × 10^−20^	0·88 (0·86–0·90)	1·98 × 10^−39^	0·89 (0·87–0·90)	1·39 × 10^−58^	0·88 (0·87–0·90)
rs80319144	2	159199835	T	C	0·24	CCDC148*‡, *PKP4*†‡, *TANC1*‡	3·18 × 10^−14^	0·89 (0·85–0·92)	1·40 × 10^−22^	0·90 (0·89–0·92)	2·55 × 10^−26^	0·90 (0·88–0·92)
rs1848460	3	3448144	T	A	0·26	*CNTN4*‡, CRBN*‡, *LRRN1*‡	5·38 × 10^−14^	1·13 (1·08–1·17)	1·93 × 10^−9^	1·06 (1·04–1·08)	2·01 × 10^−13^	1·07 (1·05–1·10)
rs35987657	3	130535567	G	A	0·33	ATP2C1*‡, *ASTE1*†	4·37 × 10^−13^	0·90 (0·88–0·91)	3·34 × 10^−23^	0·91 (0·90–0·93)	3·96 × 10^−38^	0·90 (0·89–0·92)
rs17636328	6	37490531	G	A	0·20	*RNF8*†, CCDC167*, *MDGA1*†‡	6·43 × 10^−11^	0·89 (0·85–0·92)	7·63 × 10^−18^	0·90 (0·89–0·92)	2·55 × 10^−26^	0·90 (0·88–0·92)
rs61192259	6§	38453962	A	C	0·59	BTBD9*†‡¶, *GLO1*†	1·36 × 10^−78^	1·31 (1·28–1·34)	1·05 × 10^−112^	1·22 (1·20–1·25)	3·58 × 10^−202^	1·26 (1·25–1·28)
rs10952927	7	88359060	G	A	0·13	*ADAM2*‡, *STEAP4*‡, ZNF804B*‡	1·86 × 10^−15^	1·17 (1·13–1·22)	5·01 × 10^−17^	1·12 (1·09–1·14)	1·73 × 10^−34^	1·13 (1·11–1·15)
rs1836229	9§	8820573	G	A	0·48	PTPRD*†‡	1·94 × 10^−15^	0·90 (0·88–0·91)	1·57 × 10^−29^	0·90 (0·89–0·92)	7·36 × 10^−42^	0·90 (0·89–0·91)
rs62535767	9§	9290311	T	C	0·32	PTPRD*†‡	3·13 × 10^−10^	0·91 (0·88–0·95)	8·77 × 10^−7^	0·95 (0·93–0·97)	3·23 × 10^−9^	0·94 (0·93–0·96)
rs340561	13	72848156	T	G	0·20	DACH1*†‡, *DIS3*†	3·93 × 10^−8^	1·09 (1·05–1·14)	4·91 × 10^−7^	1·05 (1·03–1·07)	3·23 × 10^−9^	1·06 (1·04–1·08)
rs996064	15	36208998	T	A	0·06	DPH6*, *MEIS2*‡	2·96 × 10^−9^	1·21 (1·14–1·28)	5·45 × 10^−21^	1·22 (1·17–1·27)	3·39 × 10^−27^	1·22 (1·17–1·26)
rs111652004	15	47360367	T	G	0·10	SEMA6D*†‡	1·05 × 10^−10^	0·84 (0·80–0·89)	3·83 × 10^−17^	0·87 (0·84–0·90)	2·69 × 10^−16^	0·86 (0·83–0·89)
rs868036	15§	68055013	T	A	0·32	*SMAD3*†, MAP2K5*¶, *SKOR1*¶, *CLN6*†	1·09 × 10^−48^	0·80 (0·77–0·83)	9·23 × 10^−70^	0·85 (0·84–0·87)	5·48 × 10^−69^	0·84 (0·83–0·86)
rs45544231	16§	52632730	G	C	0·42	TOX3*‡¶	4·72 × 10^−48^	0·81 (0·79–0·83)	4·36 × 10^−87^	0·84 (0·83–0·86)	7·27 × 10^−133^	0·83 (0·81–0·84)
rs12450895	17	46772776	A	G	0·21	*HOXB cluster*†‡, PRAC1*	4·87 × 10^−8^	1·09 (1·05–1·14)	2·01 × 10^−10^	1·07 (1·05–1·09)	4·27 × 10^−14^	1·08 (1·06–1·10)
rs12962305	18	41870243	T	C	0·25	SETBP1*‡	1·37 × 10^−10^	1·11 (1·06–1·15)	6·59 × 10^−5^	1·04 (1·02–1·06)	1·11 × 10^−7^	1·05 (1·03–1·07)
rs365032	20	62795405	G	A	0·27	MYT1*†‡	3·36 × 10^−14^	1·13 (1·08–1·17)	7·83 × 10^−36^	1·13 (1·11–1·15)	1·73 × 10^−34^	1·13 (1·11–1·15)

All p values were obtained by fixed-effect inverse-variance meta-analyses. The threshold for genome-wide significance was p≤5 × 10^−8^. For each locus, only the selected protein-coding genes are listed by genomic position (direction 5′ to 3′ on chromosome). GWAS=genome-wide association study. Position=GRCh37/hg19 coordinates. bp=base pair.

* Selected by nearest gene.

† Selected by BI-ENRICH prioritisation (nominal p<0·05).

‡ Selected by manual annotation.

§ Loci already discovered in previous GWASs.^4^, ^6^, ^7^

¶ Gene reported in previous GWASs.^4^, ^6^, ^7^

Association data for all 20 signals were obtained from the replication dataset, which included 30 770 cases and 286 913 controls (appendix p 10). All 20 association signals of the discovery stage were replicated, three at a Bonferroni-corrected level of p<0·0025, and 17 meeting genome-wide significance (table 1).

In the joint analysis of discovery and replication stages, all loci but one had genome-wide significance in the fixed-effect meta-analysis (table 1). All loci in the joint random-effects meta-analysis had genome-wide significance (appendix p 15). Overall, 13 new risk loci were identified and all six known genomic risk loci for restless legs syndrome were replicated (figure 1, table 1). The *MEIS1* locus on chromosome 2 was confirmed as the strongest genetic risk factor for restless legs syndrome (table 1). The lead SNP, rs113851554, located in a putative regulatory element in intron eight of *MEIS1,* is a low-frequency variant with odds ratio (OR) estimates of 1·82–2·16, which clearly distinguish it from the other risk loci for restless legs syndrome. The amount of heritability of restless legs syndrome attributable to all SNPs available in our dataset in the LD-score regression was 19·6%. Focusing on the 20 independent association signals, these explained 11·7% of the observed variance (60% of the SNP heritability). In the assessment of distribution of genetic risk, calculated from polygenic risk scores based on the 20 association signals (appendix p 6), individuals in the highest risk group (polygenic risk score >4·4, 99·5% quantile), had a significantly increased risk of restless legs syndrome (OR 17·6, 95% CI 8·5–42·3, p=6·9 × 10^−26^). As this group represents only a very small subset of the population, we also compared the genetic risk profiles of the 25% quantile (polygenic risk score <2·48) and the 75% quantile (polygenic risk score >3·15) of our population, which gave an OR of 5·9 (95% CI 5·3–6·5, p=8·5 × 10^−304^). The distributions of the polygenic risk scores, however, substantially overlapped between the cases and controls, which precluded the use of these scores for risk prediction (appendix p 43).

To identify biological pathways related to restless legs syndrome, we annotated protein-coding genes in or close to the genomic risk loci we identified. Six loci contained only one gene and had no genes in the immediate vicinity: three of the newly identified loci, *SEMA6D* (15q21·1), *SETBP1* (18q12·3), *MYT1* (20q13·33), and three of the previously described genes, *MEIS1* (2p14), *PTPRD* (9p24·1–p23), and *TOX3* (16q12·1). These genes function in axonal pathfinding and signalling, synaptogenesis, neuronal differentiation, and neurogenesis (table 2). Most of the remaining loci also contained genes linked to neurodevelopment, among others (table 2). A basic annotation for additional genes linked to the risk loci and regional association plots for all loci are provided in the appendix (pp 16–19, pp 44–86).

**Table 2:** Candidate genes linked to functions in neurodevelopment

	**Genes**	**Functions related to neurodevelopment**
rs12046503	*NTNG1*	Presynaptic cell-adhesion molecule involved in synapse formation^18^
rs10208712	*DCDC2C*	Encodes neuronal migration protein doublecortin, a member of the DCX protein family of cell-adhesion molecules; unknown function, but other members of the DCX family act in neuronal migration and axonal growth and have been linked to neurological and developmental disorders^19^
rs113851554 rs1820989	*MEIS1*	Implicated in neurogenesis, specification of neuronal cell type, and establishing connectivity between neurons and their target field; binds HOX proteins of all paralogue groups, participates in controlling *HOX* gene expression^20^
rs80319144	*PKP4*	Encodes the cell-adhesion molecule plakophilin-4, which serves as a scaffold for signalling complexes and plays a part in cell adhesion and neurite outgrowth^21^
rs1848460	*CRBN, CNTN4*	Cereblon, encoded by *CRBN*, is the substrate receptor of a Cullin4a RING E3 ubiquitin ligase and regulates assembly and expression of calcium-activated potassium channels in the brain;^22^, ^23^ contactin-4, encoded by *CNTN4*, is a cell-adhesion molecule with an important role in axon guidance, synapse formation, and neuronal network plasticity^24^
rs17636328	*MDGA1*	Encodes MAM domain-containing glycosylphosphatidylinositol anchor protein 1, which is a trans-synaptic cell-adhesion molecule implicated in synapse development^25^, ^26^
rs10952927	*ZNF804B, ADAM22*	ZNF804B, which is highly homologous to ZNF804A, has been associated with schizophrenia and bipolar disorder;^27^ ADAM22 is a synaptic receptor involved in synaptic transmission and synaptic disorders^28^
rs1836229 rs62535767	*PTPRD*	Related to functions in axon guidance and synaptogenesis, especially in the formation of excitatory synapses^29^, ^30^
rs340561	*DACH1*	Dach1 is a transcription factor acting as a neurogenic cell-fate determining factor^31^
rs996064	*MEIS2*	Involved in neurogenesis and contributes to determination of dopaminergic-cell fate; binds HOX proteins of all paralogue groups and participates in controlling expression of *HOX* genes^32^
rs111652004	*SEMA6D*	Involved in axonal pathfinding and signalling; exerts repulsive or attractive effects on axons, depending on the specific combinations of its main receptor with co-receptors; *SEMA6D* knockout mice show misdirection of proprioceptive axons and their associated oligodendrocytes in the dorsal horn, affecting proper synapse formation^33^, ^34^
rs45544231	*TOX3*	Implicated in neurogenesis, specification of neuronal cell type, and establishing connectivity between neurons and their target fields^35^
rs12450895	*HOXB cluster* family	Assign positional identities to neurons along the rostrocaudal axis in hindbrain and spinal cord, which is crucial in the specification of neural subpopulations and their target cells; mouse models show the necessity of *Hoxb* genes for correct neuronal specification, migration, and circuit formation^36^, ^37^
rs365032	*MYT1*	Myt1 kinase is a transcription factor expressed in neural progenitor cells in the central and peripheral nervous systems; involved in neuronal differentiation by suppressing neural progenitor fate and promoting neurogenesis^38^

Only risk loci with candidate genes linked to functions in neurodevelopment are listed. Annotation of the genes was done as described in the appendix (p 6).

**Figure 2:**
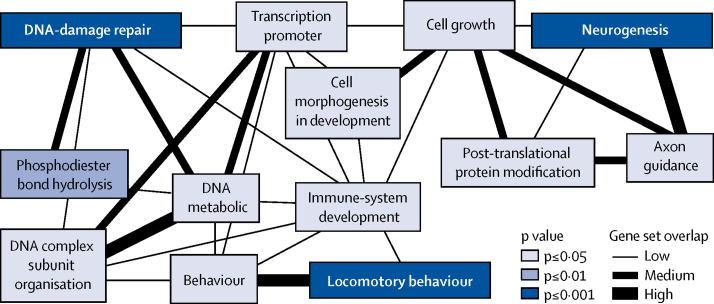
Representation of significantly enriched functional gene sets found by the BI-ENRICH analysis Similarities between gene sets are measured with the Jaccard index, with low being *J*<0·3, medium being *J*≥0·3 to <0·5, and high being *J*≥0·5. Empirical p values are shown.

These observations were substantiated by bioinformatic enrichment and gene prioritisation analyses. The BI-ENRICH algorithm showed that most of the top pathways identified for restless legs syndrome were related to neurodevelopment, including neurogenesis (genes *MDGA1, MYT1, NTNG1*, and *SEMA6D*), cell-junction organisation (*PKP4* and *SMAD3*), and axon guidance (*NTNG1* and *SEMA6D*). Moreover, locomotor behaviour (*BTBD9, CLN6, HOXB8*, and *MEIS1*) was highlighted (figure 2, appendix pp 20–22). Pathways related to DNA repair and maintenance (*APLF, ASTE1, DIS3, PRMT6*, and *RNF8*) were also detected by the enrichment analysis. Consistent with these results, BI-ENRICH prioritised genes related to neurodevelopment and DNA-damage repair (appendix pp 23–30). Use of the standard version of DEPICT returned no significant results (appendix pp 31–32). However, including the UniProtKB biological process annotations in the gene-set definitions, genes prioritised by DEPICT were enriched for neurogenesis (false discovery rate <0·05, appendix p 33). Finally, we did stratified LD-score regression to partition the heritability carried by all SNPs to specific functional categories. 14·8% of the SNPs that reside in regions associated with histone marks in the CNS explain 44·7% of the variance. This CNS enrichment is significant (enrichment factor 44·7/14·8=3·0, p=0·0043). Consistent with this observation, tissue enrichment analyses with BI-ENRICH and DEPICT ranked brain and spinal cord tissues at the top, but false discovery rates were greater than 0·2, and only spinal-cord tissue (BI-ENRICH) and mesencephalon (DEPICT) were significant (appendix pp 34–37).

**Figure 3:**
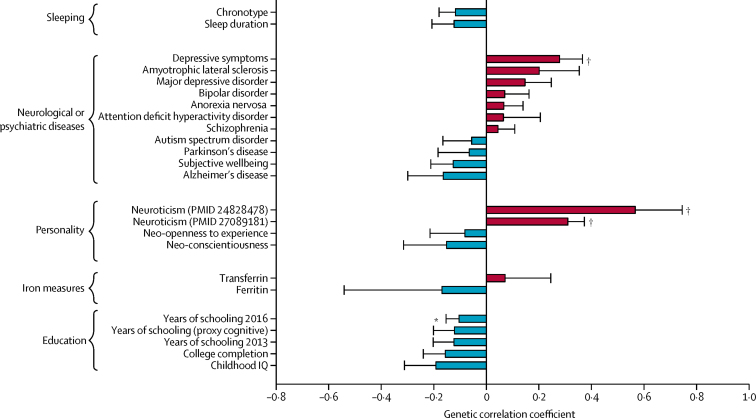
Genetic correlation between restless legs syndrome and other traits Data are mean (SE) correlations, based on linkage disequilibrium score regression in LD-Hub (appendix pp 5–6). PMID=PubMed article unique identifier. *p<0·05. †p<0·005.

Finally, we used LD-score regression to assess the genetic correlation between restless legs syndrome and diseases and traits of interest (figure 3, appendix p 38). Restless legs syndrome showed some positive correlations with neuropsychiatric diseases, whereas correlations were mainly negative with neurodegenerative disorders (figure 3), although none reached nominal significance. Few supporting data are available from corresponding epidemiological studies. Two small studies showed increased prevalence of restless legs syndrome among people with amyotrophic lateral sclerosis.^39^, ^40^ One study showed prevalence of 4% among people with Alzheimer's disease, which is lower than the average 6–12% in elderly people of European descent,^41^, ^42^ but making a diagnosis of restless legs syndrome in cognitively impaired individuals is challenging and the findings are difficult to interpret. Data on restless legs syndrome and Parkinson's disease were inconclusive, showing wide-ranging prevalence (0–50%), with some studies proposing restless legs syndrome as a risk factor for Parkinson's disease and others showing protective effects.^43^ Moreover, dopaminergic treatment of Parkinson's disease has been suggested to precipitate restless legs syndrome in some patients.^44^ The risk locus on chromosome 15q23 (encompassing *MAP2K5* and *SKOR1*) overlaps with GWAS signals of four different traits, including the posterior cortical atrophy variant of Alzheimer's disease (appendix pp 39–41). The ORs of the shared SNP rs11637445 were 1·48 for posterior cortical atrophy and 0·89 for restless legs syndrome, which is consistent with the negative correlation between these two disorders. We found a significant positive correlation between restless legs syndrome and depressive symptom phenotypes and with neuroticism (figure 3, appendix p 38). These genetic correlations are in line with epidemiological associations, which show increased prevalence of these phenotypes in patients with restless legs syndrome.^42^, ^45^ Overall, the correlations between restless legs syndrome and neurological or psychiatric phenotypes were low to moderate. We found a positive genetic correlation with number of children (*r*=0·22, p=0·0079). Pregnancy is a validated risk factor for restless legs syndrome, and parity increases the risk of developing restless legs syndrome in later life.^46^ Sleep-related phenotypes and iron-related traits showed correlations in line with published data,^1^, ^47^ but none reached nominal significance. Finally, negative genetic correlations were generally seen between restless legs syndrome and measures of educational attainment (figure 3), although this association does not seem to be widely reported.

## Discussion

This association study identified 20 independent association signals that reached genome-wide significance in 19 risk loci, of which 13 had not been previously reported. A subset of these signals showed between-study heterogeneity in our meta-analyses; in a random-effects meta-analysis that accounted for heterogeneity, all reached genome-wide significance. Effect-size estimates and strength of the association signal in these loci varied between studies, probably because of differences in phenotyping methods (clinical face-to-face interview, self-reporting by participants via a validated questionnaire, and self-reporting by use of one question). These differences can lead to variation in the proportions of misclassified cases and controls and might have modified some of the association signals. Moreover, the specific features of the restless legs syndrome phenotype, such as disease severity or preponderance of motor or sensory symptoms, might have varied between the studies. Detailed clinical data were scarce, and the restless legs syndrome phenotypes in the INTERVAL and 23andMe datasets were not clinically validated and, therefore, we cannot further address the underlying causes of heterogeneity. Studies including detailed assessments of restless legs syndrome symptoms are needed to dissect the specific roles of and possible interactions between genetic variants in the risk loci and the resulting phenotypes.

Together, the 20 association signals accounted for 60% of the SNP-based heritability of 19·6% estimated in this study. Our dataset was limited to SNPs and small indels with minor allele frequency of 1% or greater. Therefore, we could not assess the contribution of rarer or structural variants, which are likely to have larger effect sizes and might explain another part of the heritability of restless legs syndrome. Future large-scale whole-genome sequencing efforts might have the power to detect such variants.

The main aim of our study was to provide new clues to understanding the biology of restless legs syndrome. Candidate genes and pathway analyses across the 19 risk loci converge on functions important in the development of the CNS, such as neurogenesis and neural-circuit formation, including axon guidance and synaptogenesis. Additionally, the BI-ENRICH analysis highlighted DNA-damage repair, which is important for development and maintenance of the nervous system, as having a relevant role.^48^, ^49^ The involvement of perturbations in neurodevelopmental processes is in line with previous functional studies of *MEIS1* that identified the embryonic ganglionic eminences as relevant structures of restless legs syndrome biology.^50^ Our pathway analysis was designed to have high sensitivity to avoid downward bias, such as false-negative results due to incomplete information provided by the annotation databases. To avoid upward bias (ie, false-positive enrichment results), we corrected by sampling and null-GWAS permutation (phenotype label permutation). Significance of results in the tissue enrichment analyses might have been hindered by a lack of appropriate samples from relevant anatomical regions or developmental stages in the input datasets. Nonetheless, neuronal tissues were consistently prioritised, and nominally significant regions, such as midbrain structures and spinal cord, have previously been implicated in restless legs syndrome.^51^

At present, major concepts in the pathophysiology of restless legs syndrome address alterations in the dopaminergic neurotransmitter system and in brain iron metabolism, and are supported by evidence from animal, imaging, and human post-mortem studies.^47^ Even though our enrichment analyses did not specifically highlight corresponding biological processes, the new risk loci offer potential bases for functional studies that might shed light on mechanisms underlying the suspected changes of the dopaminergic neurotransmitter system or brain iron metabolism. Moderate, albeit non-significant, positive genetic correlations are being reported with neuropsychiatric disorders, such as schizophrenia or bipolar disorder, for which evidence of contributing pathological events that affect early neurodevelopment and the correct setup of neuronal circuitry is growing.^52^, ^53^

Finally, annotation of the risk loci identified a gene with a product targeted by an existing drug that is readily available for repurposing. The ubiquitin ligase substrate receptor, cereblon, which is encoded by *CRBN* (locus on chromosome 3p26) is a target of the drug thalidomide, and homeobox protein Meis2, which is encoded by *MEIS2* (locus on chromosome 15q14), has been identified as an endogenous substrate of cereblon.^33^ Thalidomide was initially licensed as a hypnotic, but was withdrawn from the market because of teratogenicity. Thalidomide and its analogues are now used as immunomodulatory drugs in cancer. They block binding of homeobox protein Meis2 to the ubiquitin ligase, thereby modulating its activity.^20^ Thalidomide and thalidomide-like substances, therefore, could be potential candidates for therapeutic use in restless legs syndrome, including in women beyond reproductive age. Moreover, this finding might also provide clues to the sleep-promoting mechanisms of this drug. Carefully designed clinical trials are needed to further investigate these drugs.

With the 13 new risk loci identified for restless legs syndrome, the total becomes 19. Our results suggest that altered embryonic neurodevelopment, impaired neurogenesis at later age, or both, could underlie restless legs syndrome pathogenesis. The exact molecular mechanisms, the relevant times in life, and the connection to brain iron metabolism and the dopaminergic system are unknown, but the implicated loci, candidate genes, and candidate pathways will provide impetus for further functional research in restless legs syndrome. Finally, the possibility of repurposing thalidomide could lead to translation of these research findings into the care of patients.
