# Supplementary material for: Identification of novel risk loci for restless legs syndrome in genome-wide association studies in individuals of European ancestry: a meta-analysis
Source: Lancet Neurol. 2017 Nov;16(11):898–907. doi: 10.1016/S1474-4422(17)30327-7 (PMC5755468; doi:10.1016/S1474-4422(17)30327-7)
Supplement: Supplementary appendix [file mmc1.pdf]

THE LANCET
Neurology

Supplementary appendix

This appendix formed part of the original submission and has been peer reviewed. We post it as supplied by the authors.

Supplement to: Schormair B, Zhao C, Bell S, et al, in collaboration with the 23andMe Research Team and the DESIR study group. Identification of novel risk loci for restless legs syndrome in genome-wide association studies in individuals of European ancestry: a meta-analysis. *Lancet Neurol* 2017; **16:** 898–907.

Supplementary Appendix

Supplement to

Barbara Schormair, Chen Zhao, 23andMe Research Team, Steven Bell et al. Identification of novel risk loci for restless legs syndrome: a meta-analysis of genome-wide association studies in individuals of European ancestry

Supplementary Methods, Tables and Figures – overview

1. Supplementary Methods

2. Supplementary Tables

Supplementary Table 1: Demographic data of study populations

Supplementary Table 2: Summary of quality control and statistical analysis procedures of individual studies

Supplementary Table 3: Results of conditional analysis using stepwise model selection procedures implemented in GCTA (--cojo-slct)

Supplementary Table 4: Assessment of heterogeneity in discovery stage meta-analysis and results of random-effects model in discovery stage

Supplementary Table 5: Results of random-effects meta-analysis of the joint analysis of discovery and replication stage

Supplementary Table 6: Basic annotation of selected candidate genes in risk loci

Supplementary Table 7: Results of geneset (pathway) enrichment analysis using BI-ENRICH

Supplementary Table 8: Results of gene prioritization analysis using BI-ENRICH

Supplementary Table 9: Results of gene set (pathway) enrichment analysis using DEPICT

Supplementary Table 10: Results of gene set (pathway) analysis using DEPICT and UniProtKB annotations

Supplementary Table 11: Results of tissue enrichment analysis using BI-ENRICH

Supplementary Table 12: Results of tissue enrichment analysis using DEPICT

Supplementary Table 13: Results of genetic correlation analysis of RLS versus traits in LD-hub

Supplementary Table 14: Overlap of RLS risk loci with NHGRI-EBI GWAS catalog signals

Supplementary Table 15: Results of gene prioritization analysis using DEPICT

3. Supplementary Figures

Supplementary Figure 1: Regional association plots of all genome-wide significant association signals of the discovery stage meta-analysis

Supplementary Figure 2: Density plot for polygenic risk score showing distribution of scores in EU-RLS-GENE study

Data availability statement

Summary statistics for the EU-RLS-GENE GWAS dataset will be made available to qualified researchers. Please contact Juliane Winkelmann (winkelmann@lrz.tu-muenchen.de) for details and to apply to access the data. Correspondence and requests for materials relating to the UK INTERVAL data set should be addressed to Dr Emanuele Di Angelantonio (ed303@medschl.cam.ac.uk) and Prof John Danesh (jd292@medschl.cam.ac.uk). Summary statistics for the 23andMe dataset will be made available through 23andMe to qualified researchers under an agreement with 23andMe that protects the privacy of the 23andMe participants. Please contact David Hinds (dhinds@23andme.com) for more information and to apply to access the data.

The code and manual to reproduce the results for the pathway analysis in manuscript figure 2 are available on GitHub (https://github.com/Chen-Zhao/BI-ENRICH-LN). And additional necessary code and data will be available on request to reproduce the results in the paper.

1. Supplementary Methods

Genotyping, quality control, imputation and statistical analysis in individual GWAS

EU-RLS-GENE consortium GWAS: All samples were genotyped on the Affymetrix Axiom CEU array. Genotyping was performed following the manufacturer's protocol. According to best practice recommendations by the manufacturer, genotype calling was performed in batches based on genotyping center and genotyping date using the Axiom GT1 algorithm.

Individuals were excluded if they had a call rate < 98%, had incorrect or ambiguous sex calls or showed potential sample contamination. We excluded related individuals (PIHAT ≥ 0.09375) and population outliers (deviation ≥ 4 SD from the population mean in MDS analysis). SNPs were excluded if they had a call rate < 98%, a MAF < 0·01, showed > 1 discordant genotype in duplicate samples, had a p value for deviation from Hardy-Weinberg-Equilibrium (p_{HWE}) ≤ 1 x 10^{-5} in controls or failed cluster quality criteria implemented in the Affymetrix SNPolisher R package.

Imputation in the EU-RLS-GENE consortium GWAS was carried out in a two-step procedure. First, study genotypes were pre-phased to produce best-guess haplotypes. This was followed by imputation into these haplotypes with the 1000Genomes Phase 1 reference panel (Phase I integrated variant set release version 3). Pre-phasing was carried out per chromosome using SHAPEIT2 with standard settings, followed by imputation with IMPUTE2 in all samples in chunks of 5 Mb with a buffer of 250 kb. SNPs with p_{HWE} ≤ 1 x 10^{-5} and an IMPUTE2 info score < 0·5 were filtered out.

Genetic association analysis was performed in SNPTEST v2.5.4 with genotype dosages using an additive model. Age, sex, and the first ten principal components from the MDS analysis in PLINK were included as covariates.[1]

UK INTERVAL GWAS study: Samples were genotyped on the Affymetrix UK Biobank Axiom array. Genotype calling was performed using the Axiom GT1 algorithm following the manufacturer's recommendations. Individuals were excluded if they had a call rate < 97%, showed potential sample contamination (> 10% or > 3% and > 10 close relatives), had a sex mismatch or missing sex information. In addition, individuals not of European ancestry were excluded (PCA-based scores on PC1 or PC2 < 0). For SNPs, a call rate filter of 99% over the batches that a variant was not failed in, and a global call rate filter of 75% (effectively ensuring a variant passed in at least eight of the ten genotype batches) were used. SNPs with a MAF < 0·005. were excluded, as well as monomorphic variants, non-autosomal and multi-allelic variants. We also excluded variants from all batches if they failed in at least four of the batches due to deviation from HWE (p_{HWE} < 5 x 10^{-6}), low call rate or Affymetrix variant exclusion criteria. Imputation was carried out in a two-step procedure for all autosomes. IMPUTE3 was used for phasing the study genotypes in sets of 5,000 variants with an overlap of 250 variants between sets. Imputation was performed using the Sanger Imputation Server (https://imputation.sanger.ac.uk), which implements the Burrows-Wheeler transform imputation algorithm PBWT and analyses whole chromosomes.[2,3] The combined UK10K + 1000Genomes Phase 3 reference panel was used as provided by the Imputation Server.

Imputed variants with a MAF < 0·005 and/or info score > 3 were excluded from statistical analysis. Genetic association analysis was performed in SNPTEST 2.5.2 as logistic regression using probabilistic imputed allele dosages with adjustment for age, sex and the first 5 ancestry principal components.

23andMe GWAS study and replication dataset: Genotyping, quality control, imputation and statistical analysis were similar for both independent datasets. Research participants were genotyped on one of four genotyping platforms. The V1 and V2 platforms were variants of the Illumina HumanHap550+ BeadChip, with added custom content by 23andMe. The V3 platform is a variant of the Illumina OmniExpress+ BeadChip, again with custom content. The V4 platform is a fully custom design.

Participants were restricted to a set of individuals who have > 97% European ancestry, as determined through an analysis of local ancestry. A maximal set of unrelated individuals was chosen for each analysis using a segmental identity-by-descent (IBD) estimation algorithm. Individuals were defined as related if they shared more than 700 cM IBD, including regions where the two individuals share either one or both genomic segments identical-by-descent. This level of relatedness (roughly 20% of the genome) corresponds approximately to the minimal expected sharing

between first cousins in an outbred population. SNPs with $p_{HWE} \leq 1 \times 10^{-20}$, a call rate < 95%, or with large allele frequency discrepancies compared to European 1000Genomes reference data were excluded.

Participant genotype data were imputed in a two-step procedure using the same reference panel and version as the EU-RLS-GENE consortium GWAS. Pre-phasing and imputation were carried out separately for each genotyping platform used in the 23andMe dataset. First, Beagle (version 3.3.1) was used to phase batches of 8000-9000 individuals across chromosomal segments of no more than 10,000 genotyped SNPs, with overlaps of 200 SNPs. Then each phased segment was imputed against all-ethnicity 1000Genomes haplotypes (excluding monomorphic and singleton sites) using Minimac2, using 5 rounds and 200 states for parameter estimation.

Variants with average $r^2 < 0{\cdot}5$ or minimum $r^2 < 0{\cdot}3$ in any imputation batch, as well as SNPs that had strong evidence of an imputation batch effect were removed prior to statistical analysis. Association analysis was carried out by logistic regression (likelihood ratio test) assuming additive allelic effects and imputed dosages. Age, sex and the top five principal components were included as covariates to account for residual population structure. In the replication dataset, this association analysis was carried out for the 20 genome-wide significant SNPs of the discovery stage.

Meta-analysis of discovery stage and joint analysis of discovery and replication stage

We performed additional quality-control of the input GWAS datasets, which had undergone stringent quality control in the individual studies, using the software EasyQC.[4] Following an initial check with default settings to ensure that no systematic errors were present in the input files, we performed a final filtering to exclude variants with a MAF < 0.01 and an info score < 0.5. Only high-quality markers with association data in all three studies were kept for subsequent analyses.

Meta-analysis of discovery stage and joint analysis of discovery and replication stage was carried out as a fixed-effect inverse-variance meta-analysis (STERR scheme) using METAL (release 2011-03-25). One round of genomic control was performed in each study prior to meta-analysis. To assess inflation in the discovery meta-analysis, the standard genomic inflation factor λ_{GC} and the rescaled λ_{1000}, a λ scaled to a study of 1000 cases and 1000 controls, were calculated. In addition, LD score regression was used to quantify the contribution of true polygenicity and biases such as cryptic relatedness and population stratification to the summary statistics of the meta-analysis.[5]

In order to account for the heterogeneity between the different studies in the discovery stage, the data was also analyzed with a random-effects model implemented in METASOFT (v2.0.1) using default settings.[6]

Identification of genome-wide significant SNPs

To define independent genome-wide significant signals in the discovery meta-analysis results, we first used the “clump” command of PLINK (v1.90b3.36) to group SNPs into LD-based independent “clumps”. To this end, lead SNPs were required to have a p value $< 5 \times 10^{-8}$ and the SNPs to be clumped had to have a p value $< 1 \times 10^{-5}$, an r^2 to the lead SNP $> 0{\cdot}05$ (LD based on EU RLS GENE genotype data), and a distance to the lead SNP < 500 kb. Thereby, 35 independent clumps were generated.

Second, we adopted the stepwise model selection procedure implemented in GCTA, to select long-distance independently associated SNPs between clumps, taking long-range LD into account.[7] The conditional analysis was performed on all 35 SNP clumps, using GCTA default settings (p value threshold for genome-wide significance (--cojo-p) set at 5×10^{-8}, distance window (--cojo-wind) set at 10 Mb, and collinearity cutoff (--cojo-collinear) at 0.9). LD was calculated again based on EU RLS GENE genotype data.

Heritability and genetic correlation analysis

Here, we estimated heritability, partitioned heritability, and genetic correlations using LD score regression as implemented in LDSC (v1.0.0) and LD-Hub (v1.2.2). Briefly, these applications are extensions of the LD score regression approach developed to measure the contributions of polygenicity and confounders to the inflation of test statistics seen in GWAS.[5] These methods account for the LD structure between SNPs by estimating the LD score of each variant, which measures the amount of genetic variation tagged by it.[5] They use GWAS summary-level results data to estimate the SNP-heritability of complex diseases, partition this heritability into functional categories defined

by genomic marks such as e.g. histone modifications. Moreover, genetic correlations between different phenotypes can be calculated if summary statistics of GWAS of the different traits are available.

We applied these methods to our dataset following the recommendations given in the respective manuscripts using the genome-wide summary statistics of our discovery meta-analysis without genomic control correction.[8-10] For the analyses, we limited our dataset to SNPs which had a MAF > 0·01, and a Chi-square statistic < 80, were located outside of the MHC region, and could be mapped to SNPs in HapMap. The summary statistics of SNPs fulfilling these criteria were the input data for the following analyses. The study sample size N is a critical multiplier in LD score regression. In order to avoid introducing a bias in case of a skewed case-control ratio, N needs to be corrected, yielding the effective sample size $N' = 4C(N-C)/N$ instead of the total sample size N with C being the number of cases.[11] Heritability and partitioned heritability were calculated with the LDSC software using the default analysis settings.[8,9] LD score for the regression is EU population based (LDSC options used: --w-ld-chr eur_w_ld_chr --ref-ld-chr eur_w_ld_chr), which is provided by LDSC v1.0.0 at (https://data.broadinstitute.org/alkesgroup/LDSCORE/). For partitioned heritability calculations, we used precomputed partitioned LD scores for 52 functional categories based on 1000Genomes Phase 3, which are provided by the developers of LDSC and are recommend for this type of analysis (LDSC options used: --ref-ld-chr baseline. --w-ld-chr weights).[10] For the genetic correlation analysis between RLS and other traits, we used the LD-Hub[8] interface, a centralized online database which stores the summary-level GWAS data for more than 100 traits and provides a web interface for the calculation.

To estimate variance explained by genome-wide significant association signals, we calculated Nagelkerke's pseudo-R^2 in the EU-RLS-GENE dataset with 10-fold cross validation.

Genetic risk score analysis

To calculate polygenic risk scores (PRS), we estimated the weighted polygenic risk score in the EU-RLS-GENE study, where genotype dosage data was available for all individuals. The weights were obtained from the discovery stage meta-analysis (fixed-effect model). PRSice v1.25 was used to calculate the PRS.[13] The relative distribution of the PRS in the EU-RLS-GENE dataset was plotted using the ggplot2 R package.

Biological Interpretation of association signals

Manual gene annotation: We searched PubMed for all protein-coding genes which i) contained associated variants or their LD proxies, ii) were located in the LD blocks tagged by the association signal ($r^2 > 0·05$), or which iii), for loci containing no genes, were located in the vicinity of the LD block (± 500 kb from lead SNP and $r^2 > 0·05$).

BI-ENRICH algorithm: Adopting and extending the framework of DEPICT, we implemented a sensitive and efficient method which we called BI-ENRICH. Our algorithm builds on the concept of biclustering used in gene expression analysis.[13] Briefly, it first iteratively tests for enrichment of the candidate genes in pathways (tissues). If a gene has already been assigned to a pathway (tissue) in a previous round, it is excluded from the enrichment calculation for another pathway (tissue) in later rounds unless it is part of that pathway (of that tissue's expression pattern). Thereby, a "greedy search" is implemented which counteracts the overly conservative tendency of multiple testing correction in case of overlaps between the pathways (tissue expression patterns) that are tested for enrichment. The iteration stops as soon as no further pathway enrichment is indicated. The true p values of the enrichments are derived in the end from null simulations with permutated phenotype labels. BI-ENRICH also involves gene prioritization at each locus, which is based on functional or tissue-specific relatedness to genes at the other loci.

The method consists of three steps:

Step 1: Identification of the candidate genes at all GWAS loci (defined as the clumps used for "*Identification of genome-wide significant SNPs*", see above).

a. Genes overlapping a GWAS locus.
b. Genes whose expression levels are affected by a SNP within a GWAS locus, with the SNPs having a p value $< 10^{-5}$ and a significant ($p < 10^{-6}$) eQTL-effect according to the GTEx V6 eQTL dataset.
c. Genes within 1 Mb distance of the locus if the locus contains enhancer elements as defined by Roadmap or VISTA Enhancer markers.

Step 2: Enrichment analysis

The biclustering procedure of BI-ENRICH is explained here for the case of pathway enrichment. (Pathways included all GSEA curated pathways, UniProt Knowledgebase defined biological processes, and GO biological processes.). The procedure is iterative, going through several rounds, each time comparing the candidate set with all available pathways. In each round, it accumulates information which it then uses for adapting its "greedy search" in the next round. BI-ENRICH stops if in spite of the latest adaptation no further pathways overcome the defined threshold.

In detail, for each round r, a working enrichment score $q_{r,j}$ of the comparison of the candidate gene set Z with a set S_j of genes with function j, is defined as the result of a one-tailed Fisher's exact test. In the first round the score is

$$q_{1,j} =: 1 - \sum_{k=0}^{a-1} \binom{a+b}{k} \binom{c+d}{a+c-k} / \binom{n}{a+c}$$

where, among n genes with at least one function, a is the number of genes that are both in Z and in S_j, b is the number of genes in Z but not in S_j, c is the number of genes in S_j but not in Z, and d is the number of genes neither in Z nor in S_j, with $n = a + b + c + d$, as shown in the contingency table,

Geneset S_j / Candidate set Z	Yes	No
Yes	a	b
No	c	d

Here, $q_{1,j}$ equals the probability that S_j and Z just randomly share a or more of the n genes. While most other enrichment tools would take the multiple testing-corrected first-round results as final, BI-ENRICH now completes a biclustering process in the following rounds. There, it applies an indicator function $I_{g,r,j}$ for each function j and each gene g of Z which maintains that g is ignored in the Fisher calculation of $q_{r,j}$, if it does not belong to S_j, but does belong to at least one set S_i, i ≠ j, to which working enrichment was attributed in a previous round. The working enrichment threshold is defined as score q being smaller than 0·05. Thus, b decreases in each round ($b_{r,j} \geq b_{r+1,j}$) while a, c, and d remain unchanged, resulting in $q_{r+1,j} \leq q_{r,j}$, so that in each new round additional pathways may overcome the threshold. The algorithm stops if no additional pathway can be found to be enriched anymore. For the following permutation-based empirical significance analysis of the enriched pathways (see below), BI-ENRICH uses the q-scores from the latest round.

The biclustering algorithm of BI-ENRICH counteracts the overly conservative tendency of standard enrichment analysis (i.e, of the first-round outcomes) which results from Bonferroni multiple testing correction in spite of the overlaps between the multiple pathways that are tested for. However, the algorithm may inflict a bias in the other direction. Therefore, BI-ENRICH derives empirical p values and FDRs from a null distribution that is produced by 1) performing 1000 null GWASes with permuted RLS phenotype labels, 2)

running the top 20 independent loci of each null GWAS result through a BI-ENRICH enrichment analysis, and 3) using the BI-ENRICH enrichment scores of all these null GWASes as the null distribution. The permutation-based significance determination implicitly also corrects for biases such as chromosome gene density or pathway size (number of genes in the pathway). To match the gene density of the candidate loci in the permutation GWASes, a maximum of 10 genes are included for each locus which is the maximum number of genes obtained per locus in our GWAS candidate loci.

For tissue enrichment, an analogous procedure is performed in order to assign the candidate genes to one or more sets of tissues, in which they are preferentially expressed.[14] Tissue-specific expression level is provided as a *z*-score (data from GTEx, RPKM expression values). The working score for the enrichment of candidate gene expressions in a tissue is derived from a one-sided Kolmogorov–Smirnov test in a fashion analogous to the GSEA procedure[15], assessing for the candidate genes the distribution of their expression levels in that tissue. Similar to the pathway enrichment analysis, a gene is removed if, in a previous round, it contributed to the enrichment of another tissue but does not show over-expression (i.e., less than the median of all gene expressions) in the tissue presently tested. Again, p values are derived from a permutation-based null distribution.

Step 3: Gene prioritization
Based on enrichment results, a gene in a locus is prioritized if it tends to share function (pathway) or tissue enrichment with other genes at any of the associated loci.
The score of gene *g* for gene prioritization is defined as

$$P_g = \max_{i:g\in S_i} F_i + \max_t W_{gt} T_t,$$

where F_i is the *z*-score that corresponds to the empirical p value of the enrichment for function *i*, W_{gt} is the gene-wise scaled *z*-score of gene *g* in tissue *t*, and T_t is the *z*-score that is calculated based on the empirical p value for enrichment in tissue *t*. F_i and T_t increase with the numbers of genes from the candidate set that contribute to the enrichment of function *i* or tissue *t*, respectively, and the indicator functions $\max_{i:g\varepsilon Si}$ and $\max_t$ maintain that for F_i and $W_{gt}T_t$ the maximal values are chosen. For each locus the gene with the maximal prioritization score P_g is selected. Significance of P_g is derived from permutation-based null distribution.

Pathway/gene set enrichment using DEPICT: In addition to the algorithm described above, we used DEPICT rel194[16] to perform gene prioritization for each locus and to search for gene set and tissue enrichment among these genes. Parameters were set as the default values, i.e., association p value < 5 x 10^{-8}, background Plink clumping p < 5 x 10^{-4}. This resulted in 27 SNPs in 16 independent loci containing protein-coding genes for the analysis (Supplementary Table 16). Functional annotation was performed using default resources in DEPICT.

In addition, all prioritized genes from the DEPICT analysis were annotated based on UniProtKB (release-2016_08) keywords (knowledge based), biological process category, with a total of 139 categories. Hypergeometric testing of the candidate gene set was performed in pairwise comparison with all categories that contained at least one gene (GOstats package v2.36.0). The entire set of genes annotated to the biological process category was used as background.

Tissue enrichment: DEPICT and BI-ENRICH were used to identify the tissues that the genome-wide significant loci might be enriched for. The DEPICT tissue enrichment analysis was performed at the same step as the gene prioritization analysis. For the BI-ENRICH tissue enrichment analysis (see above), the expression data from 43 tissue types were included, based on GTEx V6 excluding tissue types with less than 10 replicates or belonging to sexual organs.

References for supplementary methods:

1 Marchini J, Howie B, Myers S, McVean G, Donnelly P. A new multipoint method for genome-wide association studies by imputation of genotypes. *Nat Genet* 2007; **39**: 906–13.

2 Durbin R. Efficient haplotype matching and storage using the positional Burrows-Wheeler transform (PBWT). *Bioinformatics* 2014; **30**: 1266–72.

3 McCarthy S, Das S, Kretzschmar W, *et al.* A reference panel of 64,976 haplotypes for genotype imputation. *Nat Genet* 2016; **48**: 1279–83.

4 Winkler TW, Day FR, Croteau-Chonka DC, *et al.* Quality control and conduct of genome-wide association meta-analyses. *Nat Protoc* 2014; **9**: 1192–212.

5 Bulik-Sullivan BK, Loh P-R, Finucane HK, *et al.* LD Score regression distinguishes confounding from polygenicity in genome-wide association studies. *Nat Genet* 2015; **47**: 291–5.

6 Han B, Eskin E. Random-effects model aimed at discovering associations in meta-analysis of genome-wide association studies. *Am J Hum Genet* 2011; **88**: 586–98.

7 Yang J, Lee SH, Goddard ME, Visscher PM. GCTA: a tool for genome-wide complex trait analysis. *Am J Hum Genet* 2011; **88**: 76–82.8

8 Zheng J, Erzurumluoglu AM, Elsworth BL, *et al.* LD Hub: a centralized database and web interface to perform LD score regression that maximizes the potential of summary level GWAS data for SNP heritability and genetic correlation analysis. *Bioinformatics*. 2017;**33**:272-279.

9 Bulik-Sullivan B, Finucane HK, Anttila V, et al. An Atlas of Genetic Correlations across Human Diseases and Traits. Nature genetics. 2015;47(11):1236-1241. doi:10.1038/ng.3406.

10 Finucane HK, Bulik-Sullivan B, Gusev A, *et al.* Partitioning heritability by functional annotation using genome-wide association summary statistics. *Nat Genet* 2015; **47**: 1228–35.

11 Charan J, Biswas T. How to calculate sample size for different study designs in medical research? *Indian J Psychol Med* 2013; **35**: 121.

12 Euesden J, Lewis CM, O'Reilly PF. PRSice: Polygenic Risk Score software. *Bioinformatics* 2015; **31**: 1466–8.

13 Madeira SC, Oliveira AL. Biclustering algorithms for biological data analysis: a survey. *IEEE/ACM Trans Comput Biol Bioinforma* 2004; **1**: 24–45.

14 Kryuchkova-Mostacci N, Robinson-Rechavi M. A benchmark of gene expression tissue-specificity metrics. *Brief Bioinform* 2016; : bbw008.

15 Subramanian A, Tamayo P, Mootha VK, *et al.* Gene set enrichment analysis: a knowledge-based approach for interpreting genome-wide expression profiles. *Proc Natl Acad Sci U S A* 2005; **102**: 15545–50.

16 Pers TH, Karjalainen JM, Chan Y, *et al.* Biological interpretation of genome-wide association studies using predicted gene functions. *Nat Commun* 2015; **6**: 5890.

2. Supplementary Tables

Supplementary Table 1: Demographic data of study populations. * A subset of cases (n = 954) and controls (n = 1,814) were part of the genome-wide stage of previously published GWAS on Restless Legs Syndrome (PMIDs: 17637780 and 21779176).

STUDY Details			SAMPLES						
			Samples before quality control		Samples after quality control				
Study Name	Study Reference	Ancestry	Cases (N)	Controls (N)	Cases (N)	Controls (N)	Age in Years (Mean and SD)	% female	Age at Onset in Years (Mean and SD)
EU-RLS-GENE*	na	European	6,650	11,819	6,228	10,992	57·5 (± 14·5) NA for 11	52·8	39·5 (± 18·5) na for 2,190
INTERVAL	PMID:25230735	European	3,141	25,641	3,065	24,923	46·1 (± 13·7) years	49·3	na
23andMe Discovery	na	European	5,905	64,365	5,833	59,810	51·0 (± 15·8) years	52·3	na
23andMe Replication	na	European	32,328	332,551	30,770	286,913	52·1 (± 16·7) years	56·3	na

Supplementary Table 2: Summary of quality control and statistical analysis procedures of individual studies.

	Study Name	EU-RLS-GENE	INTERVAL	23andME
**Genotyping sample QC**	**Call rate**	< 98%	< 97%	< 98%
	**other exclusions**	Individuals were excluded if they had a callrate < 98%, had incorrect or ambiguous sex calls or showed potential sample contamination. We excluded related individuals (PIHAT ≥ 0·09375) and population outliers (deviation ≥ 4 SD from the population mean in MDS analysis).	Poor signal intensity (dish QC < 0·82), potential sample contamination (>10% or >3% and > 10 close relatives), mismatch of sex phenotype/genotype or missing sex. We also excluded samples that were evidently not of European ancestry (scores < 0 on PC1 or PC2 following a PCA including INTERVAL samples with 1000 Genomes major ancestry populations), duplicate samples ($\hat{\pi} \geq$ 0·9 using PLINK Method-of-Moments IBD approach) and related individuals ($\hat{\pi} \geq$ 18.75%) using autosomal variants with MAF > 0·05, p(HWE) < 1x10-6, r2 ≤ 0·2 between pairs of variants.	We excluded individuals who had >3% non-European ancestry based on a local ancestry painting analysis using three HapMap2 reference populations, and individuals who shared more than 700 cM identical by descent
**Genotyping SNP QC**	**Platform**	Affymetrix Axiom Genome-Wide CEU Array	UK Biobank Affymetrix Axiom array	custom Illumina arrays
	**Genotype calling algorithm**	Axiom GT1 algorithm	Axiom GT1 algorithm	Illumina GenomeStudio
	**MAF**	< 0·01	< 0·005	<0·001
	**SNP Call rate**	global call rate < 98%	call rate < 99% over the batches in which a variant did not fail, and a global call rate < 75%	90%
	**p for HWE**	$< 5 \times 10^{-5}$	$< 5 \times 10^{-6}$	$< 1 \times 10^{-20}$ in 400k European individuals
	**other exclusion**	We excluded SNPs with ≥ 1 discordant genotype call in duplicate samples and variants which failed Affymetrix variant exclusion criteria in at least one of the genotyping batches.	We removed all monomorphic variants, non-autosomal and multi-allelic variants. We also excluded variants from all batches if they failed in at least four of the batches due to deviation from HWE, low call rate or Affymetrix variant exclusion criteria.	We removed SNPs with large allele frequency differences with the corresponding European 1000 Genomes reference data

Imputation	**SNPs that met QC criteria (N)**	450,602	654,966	869,862
	**Phasing software (version and settings used)**	SHAPEIT (release v2 (r644)) with standard settings and the –output-max option (MCMC: 35 iterations [7 B + 8 P + 20 M]; model: 100 states per window [100 H + 0 PM + 0 R] / Windows of ~2·0 Mb / Ne = 15000).	IMPUTE3 (sets of 5,000 variants with an overlap of 250 variants between sets)	Beagle version 3.3.1, in batches of <10,000 samples, in segments of <10,000 SNPs with overlaps of 200 SNPs
	**Imputation software (version and settings used)**	IMPUTE2 version 2.3.0· Imputation was carried out in all samples in chunks of 5 MB with a buffer of 250kb. Options in effect were: -use_prephased_g, -known_haps_g, -buffer 250, -Ne 20000, -pgs_miss, -align_by_maf_g, -os 0 1 2 3.<br>Reference panel: 1000Genomes Phase 1 reference panel (Phase I integrated variant set release version 3).	Sanger Imputation Server (https://imputation.sanger.ac.uk) which uses the Burrows-Wheeler transform imputation algorithm and analyses whole chromosomes. Imputed using a combined 1,000 Genomes Phase 3-UK10K imputation panel.	Minimac2, 5 rounds and 200 states, with 1000 Genomes Phase I v3 reference panel
	**Post imputation QC if any**	SNPs with deviation from HWE more significant than p ≤ 1 x 10-5 and an IMPUTE2 info score < 0·5 were filtered out.	We excluded imputed variants with MAF < 0·005 and/or info score >3.	We excluded variants with imputed r^2 < 0·3 in any one imputation batch or average r^2 < 0·5 across batches.
**Association analysis**	**Analysis software (version and settings used)**	SNPTEST v2.5.4. (additive model with imputed dosages (options –frequentist 1, -method expected); model adjusted for age, sex, 10 principal components from the MDS analysis in PLINK	SNPTEST v2.5.2 (logistic regression using genotype dosages [method=expected] with adjustment for age, sex and the first 5 ancestry principal components)	Custom software for logistic regression using genotype dosages, adjusted for age, sex, and 5 principal components

Supplementary Table 3: Results of conditional analysis using stepwise model selection procedures implemented in GCTA (--cojo-slct). Freq, reference allele frequency in joint meta-analysis; n, estimated effective sample size; freq_geno, genotype frequency in joint meta-analysis; bJ, beta estimate from GCTA analysis; bJ_se, standard error for beta estimate from GCTA analysis;pJ, p value from GCTA analysis.

Chr	SNP	bp	refA	freq	n	freq_geno	bJ	bJ_se	pJ
1	rs12046503	107195339	T	0·5887	115123	0·568674	-0·1621	0·0140081	5·72E-31
2	rs10208712	4034446	A	0·6404	111368	0·655376	0·1149	0·014604	3·61E-15
2	rs113851554	66750564	T	0·071	114642	0·0798813	0·759532	0·0269458	8·34E-175
2	rs1820989	68069890	A	0·4685	108853	0·516721	-0·136473	0·0142064	7·51E-22
2	rs80319144	159199835	T	0·2431	110469	0·240712	-0·1244	0·0164042	3·37E-14
3	rs1848460	3448144	A	0·7433	116246	0·748603	-0·1178	0·0157037	6·31E-14
3	rs35987657	130535567	A	0·666	112209	0·677384	0·1073	0·0148034	4·22E-13
6	rs17636328	37490531	A	0·8018	106191	0·821061	0·110709	0·0180092	7·88E-10
6	rs61192259	38453962	A	0·5945	109071	0·608717	0·25072	0·0147301	5·75E-65
7	rs10952927	88359060	A	0·8725	119256	0·868626	-0·161	0·0203053	2·21E-15
9	rs1836229	8820573	A	0·5219	113396	0·517851	0·111315	0·0139041	1·19E-15
9	rs62535767	9290311	T	0·3213	111443	0·308894	-0·0950024	0·0150028	2·42E-10
13	rs340561	72848156	T	0·2037	115293	0·203519	0·0937	0·0171022	4·28E-08
15	rs996064	36208998	A	0·9446	102040	0·956866	-0·1898	0·0320054	3·02E-09
15	rs111652004	47360367	T	0·096	90392	0·0708909	-0·1704	0·0264059	1·10E-10
15	rs868036	68055013	A	0·6806	108694	0·69756	0·2229	0·015215	1·35E-48
16	rs45544231	52632730	C	0·5789	109517	0·602101	0·2077	0·0143137	1·04E-47
17	rs12450895	46772776	A	0·2115	116181	0·202874	0·0917	0·0168021	4·82E-08
18	rs12962305	41870243	T	0·2474	116162	0·253204	0·102	0·0159027	1·42E-10
20	rs365032	62795405	A	0·7298	108297	0·781761	-0·1212	0·0160042	3·65E-14

Supplementary Table 4: Assessment of heterogeneity in discovery stage meta-analysis and results of random-effects model in discovery stage.
Chr, chromosome; Association P value, P value of SNP association to RLS observed in the respective studies; OR (95% CI), odds ratio with 95% confidence interval; direction of effect, direction of the effect size estimate for the three GWAS included in the discovery meta-analysis; I2, I2 measure of heterogeneity; Q, Cochran's Q; Q P value, P value for Cochran's Q.

		EU-RLS-GENE GWAS of discovery stage		UK INTERVAL GWAS of discovery stage		23andME GWAS of discovery stage		Discovery stage Fixed-effect meta-analysis			Discovery stage Random-effects meta-analysis				
**Lead SNP**	**Chr**	**Association P value**	**OR (95% CI)**	**Association P value**	**OR (95% CI)**	**Association P value**	**OR (95% CI)**	**association P value**	**direction of effect**	**I2**	**association P value**	**OR (95% CI)**	**I2**	**Q**	**Q P value**
rs12046503	1	6·73E-22	0·79 (0·76-0·83)	2·19E-10	0·84 (0·79-0·89)	1·70E-08	0·89 (0·86-0·93)	3·32E-31	---	84·6	1·29E-35	0·84 (0·78-0·9)	86·1	14·39	0·0008
rs10208712	2	7·22E-08	0·87 (0·83-0·92)	1·14E-05	0·88 (0·83-0·93)	5·33E-06	0·91 (0·87-0·95)	3·78E-15	---	0	4·92E-16	0·89 (0·87-0·92)	0	1·87	0·392
rs113851554	2	5·44E-179	3·55 (3·26-3·88)	4·64E-22	1·67 (1·5-1·85)	9·07E-47	1·77 (1·64-1·92)	1·1E-180	+++	98·7	6·03E-240	2·19 (1·36-3·53)	98·8	173·36	2·27E-38
rs1820989	2	3·91E-12	0·84 (0·8-0·88)	2·61E-07	0·87 (0·82-0·92)	5·92E-07	0·9 (0·87-0·94)	1·23E-20	---	50·4	3·22E-22	0·87 (0·84-0·91)	55	4·44	0·1084
rs80319144	2	2·57E-10	0·84 (0·79-0·88)	1·03E-02	0·92 (0·86-0·98)	1·76E-06	0·89 (0·85-0·94)	3·18E-14	---	58·5	1·85E-15	0·88 (0·84-0·93)	63·2	5·44	0·0658
rs1848460	3	5·75E-09	1·17 (1·11-1·24)	3·95E-04	1·12 (1·05-1·19)	1·42E-05	1·1 (1·06-1·15)	5·38E-14	+++	24·3	5·44E-15	1·13 (1·09-1·17)	33·4	3	0·2228
rs35987657	3	1·13E-03	0·92 (0·88-0·97)	4·30E-05	0·89 (0·84-0·94)	9·38E-08	0·89 (0·86-0·93)	4·37E-13	---	0	2·46E-13	0·9 (0·87-0·93)	0	1·13	0·5679
rs17636328	6	2·49E-06	0·86 (0·81-0·92)	2·20E-03	0·9 (0·84-0·96)	5·22E-05	0·9 (0·86-0·95)	6·43E-11	---	0	1·46E-11	0·89 (0·86-0·92)	0	1·37	0·5031
rs61192259	6	1·51E-32	1·34 (1·28-1·41)	3·57E-10	1·2 (1·13-1·27)	8·73E-48	1·36 (1·3-1·41)	1·36E-78	+++	84·9	9·58E-85	1·3 (1·21-1·4)	85·4	13·67	0·0011
rs10952927	7	3·56E-09	1·23 (1·15-1·32)	5·60E-03	1·12 (1·03-1·21)	5·42E-08	1·17 (1·11-1·24)	1·86E-15	+++	25·8	1·82E-16	1·18 (1·12-1·23)	33·1	2·99	0·2241
rs1836229	9	5·55E-15	0·83 (0·79-0·87)	5·24E-01	0·98 (0·93-1·04)	1·92E-08	0·89 (0·86-0·93)	1·94E-15	---	89·8	1·26E-19	0·9 (0·82-0·98)	90·7	21·49	2·16E-05
rs62535767	9	8·44E-07	0·88 (0·84-0·93)	2·00E-04	0·89 (0·84-0·95)	2·23E-03	0·94 (0·9-0·98)	3·13E-10	---	41·1	5·48E-11	0·91 (0·87-0·94)	45·7	3·68	0·1588
rs340561	13	1·09E-04	1·12 (1·06-1·19)	6·86E-04	1·12 (1·05-1·2)	4·22E-03	1·07 (1·02-1·13)	3·93E-8	+++	0	1·37E-08	1·1 (1·06-1·14)	0	1·76	0·4146
rs996064	15	1·68E-04	1·23 (1·1-1·37)	8·23E-05	1·26 (1·12-1·41)	2·10E-03	1·16 (1·06-1·28)	2·96E-9	+++	0	1·07E-09	1·21 (1·14-1·29)	0	1·13	0·5684
rs111652004	15	3·15E-06	0·8 (0·74-0·88)	4·43E-04	0·84 (0·77-0·93)	3·07E-04	0·87 (0·8-0·94)	1·05E-10	---	0	2·37E-11	0·84 (0·8-0·89)	0	1·52	0·4682
rs868036	15	1·48E-41	0·7 (0·66-0·74)	1·30E-10	0·82 (0·77-0·87)	2·08E-13	0·85 (0·82-0·89)	1·09E-48	---	93·5	1·52E-59	0·79 (0·7-0·89)	94·3	35·05	2·45E-08
rs45544231	16	9·87E-30	0·76 (0·72-0·79)	1·18E-14	0·8 (0·76-0·85)	9·04E-15	0·85 (0·82-0·89)	4·72E-48	---	84·1	9·92E-54	0·8 (0·75-0·87)	85·7	13·96	0·0009
rs12450895	17	8·72E-07	1·16 (1·09-1·22)	7·48E-04	1·12 (1·05-1·19)	3·65E-02	1·05 (1-1·1)	4·87E-8	+++	65·7	5·05E-09	1·11 (1·04-1·17)	68·6	6·37	0·0414
rs12962305	18	2·37E-04	1·11 (1·05-1·17)	1·57E-02	1·08 (1·02-1·15)	4·91E-07	1·12 (1·07-1·17)	1·37E-10	+++	0	5·94E-11	1·11 (1·07-1·14)	0	0·9	0·638
rs365032	20	9·82E-10	1·21 (1·14-1·29)	2·51E-01	1·04 (0·98-1·1)	7·63E-10	1·15 (1·1-1·2)	3·36E-14	+++	84	2·47E-16	1·13 (1·04-1·23)	85·3	13·56	0·0011

Supplementary Table 5: Results of random-effects meta-analysis of the joint analysis of discovery and replication stage. Chr, chromosome; Association P value, P value of SNP association to RLS observed in the respective meta-analysis; OR (95% CI), odds ratio with 95% confidence interval; direction of effect, direction of the effect size estimate for the two datasets (discovery and replication) included in the joint analysis; I2, I2 measure of heterogeneity; Q, Cochran's Q; Q P value, P value for Cochran's Q.

		Joint stage Fixed-effect meta-analysis				Joint stage Random-effects meta-analysis				
**Lead SNP**	**Chr**	**Association P value**	**OR (95% CI)**	**direction of effect**	**I2**	**Association P value**	**OR (95% CI)**	**I2**	**Q**	**Q P value**
rs12046503	1	3.254E-63	0·88 (0·86-0·89)	--	93.3	3.70E-27	0.88 (0.82-0.94)	72.6	3.65	0.0562
rs10208712	2	1.406E-34	0·91 (0·90-0·92)	--	73.3	3.78E-29	0.91 (0.88-0.94)	76.9	4.33	0.0374
rs113851554	2	2.0E-280	1·92 (1·85-1·99)	++	94.6	4.84E-199	1.82 (1.75-1.9)	0	0.56	0.4531
rs1820989	2	1.392E-58	0·88 (0·87-0·90)	--	0	4.81E-42	0.88 (0.87-0.9)	0	0.55	0.4581
rs80319144	2	2.553E-26	0·90 (0·88-0·92)	--	0	7.19E-28	0.9 (0.89-0.92)	0	0.85	0.3571
rs1848460	3	2.005E-13	1·07 (1·05-1·10)	++	83.3	4.34E-17	1.09 (1.03-1.16)	87.7	8.13	0.0043
rs35987657	3	3.956E-38	0·90 (0·89-0·92)	--	40	9.37E-31	0.91 (0.9-0.92)	0	0.85	0.3552
rs17636328	6	2.553E-26	0·90 (0·88-0·92)	--	0	4.19E-33	0.9 (0.89-0.92)	0	0.88	0.3475
rs61192259	6	3.58E-202	1·26 (1·25-1·28)	++	95.1	1.60E-98	1.25 (1.18-1.32)	60.4	2.53	0.1119
rs10952927	7	1.734E-34	1·13 (1·11-1·15)	++	76	1.61E-36	1.14 (1.08-1.2)	74.6	3.93	0.0474
rs1836229	9	7.357E-42	0·90 (0·89-0·91)	--	0	2.09E-24	0.9 (0.89-0.92)	0	0.03	0.8707
rs62535767	9	3.227E-09	0·94 (0·93-0·96)	--	62.5	2.88E-11	0.93 (0.89-0.98)	79	4.77	0.029
rs340561	13	3.227E-09	1·06 (1·04-1·08)	++	62.5	4.02E-13	1.07 (1.03-1.12)	81.4	5.38	0.0203
rs996064	15	3.348E-27	1·22 (1·17-1·26)	++	0	2.01E-31	1.22 (1.18-1.26)	0	0.06	0.8036
rs111652004	15	2.694E-16	0·86 (0·83-0·89)	--	0	8.74E-22	0.86 (0.83-0.89)	1.3	1.01	0.3141
rs868036	15	5.478E-69	0·84 (0·83-0·86)	--	83.3	7.66E-60	0.84 (0.79-0.89)	32.9	1.49	0.2221
rs45544231	16	7.27E-133	0·83 (0·81-0·84)	--	85	2.23E-71	0.83 (0.8-0.87)	36.8	1.58	0.2086
rs12450895	17	4.267E-14	1·08 (1·06-1·10)	++	0	2.23E-14	1.08 (1.06-1.1)	0	0.92	0.337
rs12962305	18	1.113E-07	1·05 (1·03-1·07)	++	83.3	3.32E-13	1.07 (1.01-1.14)	91.2	11.34	0.0008
rs365032	20	1.734E-34	1·13 (1·11-1·15)	++	0	1.44E-34	1.13 (1.11-1.15)	0	0	0.9719

Supplementary Table 6: Manual annotation of selected candidate genes in risk loci. A basic annotation for selected genes is provided based on information from literature research in NCBI PubMed and Gene databases (https://www.ncbi.nlm.nih.gov/pubmed/ and https://www.ncbi.nlm.nih.gov/gene).

Gene	Name	Chromosome	Lead SNP	Comment
PRMT6	Protein Arginine Methyltransferase 6	1	rs12046503	The protein encoded by this gene belongs to the arginine N-methyltransferase family, which catalyze the sequential transfer of methyl group from S-adenosyl-L-methionine to the side chain nitrogens of arginine residues within proteins, to form methylated arginine derivatives and S-adenosyl-L-homocysteine. This protein also forms a complex with, and methylates DNA polymerase beta, resulting in stimulation of polymerase activity by enhancing DNA binding and processivity. Enhancer of transcriptional activity often implicated with invasiveness of cancers.
VAV3	Vav 3 Guanine Nucleotide Exchange Factor	1	rs12046503	The VAV proteins are guanine nucleotide exchange factors (GEFs) for Rho family GTPases that activate pathways leading to actin cytoskeletal rearrangements and transcriptional alterations. Plays an important role in angiogenesis and integrin-mediated cell adhesion. Involved in GABAergic axon guidance events important for the proper function of brainstem neurons controlling cardiovascular, respiratory, and renal parameters.
SLC25A24	Solute Carrier Family 25, Member 24	1	rs12046503	Calcium-dependent mitochondrial solute carrier. Mediates the reversible, electroneutral exchange of Mg-ATP or Mg-ADP against phosphate ions, catalyzing the net uptake or efflux of adenine nucleotides across the mitochondrial inner membrane.
NTNG1	Netrin G1	1	rs12046503	Netrin G1 (NTNG1) belongs to a conserved family of proteins that act as axon guidance cues during vertebrate nervous system development. Involved in controlling patterning and neuronal circuit formation at the laminar, cellular, subcellular and synaptic levels. Promotes neurite outgrowth of both axons and dendrites. Expressed in ganglionic eminences.
DCDC2C	Doublecortin Domain Containing 2C	2	rs10208712	Function is unknown; the protein belongs to the DCX protein family. Other members of this family act in neuronal migration and axonal growth and have been linked to neurological and developmental disorders.
ALLC	Allantoicase	2	rs10208712	Allantoicase participates in the uric acid degradation pathway. Its enzymatic activity, like that of urate oxidase, was lost during vertebrate evolution.
LINC01304	Long intergenic non-protein coding RNA 1304	2	rs10208712	Non-coding RNA, mainly expressed in testis.
RPS7	Ribosomal protein S7	2	rs10208712	This gene encodes a ribosomal protein that is a component of the 40S subunit. The protein belongs to the S7E family of ribosomal proteins. It is located in the cytoplasm.
CMPK2	Mitochondrial Cytidine Monophosphate (UMP-CMP) Kinase 2	2	rs10208712	May participate in dUTP and dCTP synthesis in mitochondria. May be involved in mtDNA depletion caused by long term treatment with ddC or other pyrimidine analogs.
COLEC11	Collectin sub-family member 11	2	rs10208712	This gene encodes a member of the collectin family of C-type lectins that possess collagen-like sequences and carbohydrate recognition domains. Collectins are secreted proteins that play important roles in the innate immune system by binding to carbohydrate antigens on microorganisms, facilitating their recognition and removal.
C1D	C1D nuclear receptor corepressor	2	rs1820989	The protein encoded by this gene is a DNA binding and apoptosis-inducing protein and is localized in the nucleus. It is also a Rac3-interacting protein which acts as a corepressor for the thyroid hormone receptor. This protein is thought to regulate TRAX/Translin complex formation. Alternate splicing results in multiple transcript variants that encode the same protein.
APLF	Aprataxin And PNKP Like Factor	2	rs1820989	Component of the cellular response to chromosomal DNA damage. Nuclease involved in single-strand and double-strand DNA break repair. Displays apurinic-apyrimidinic (AP) endonuclease and 3-5 exonuclease activities in vitro.
PKP4	Plakophilin 4 (aka p0071)	2	rs80319144	Multifunctional "armadillo protein" involved in cytoskeleton organization and cell adhesion.
CCDC148	Coiled-Coil Domain Containing 148	2	rs80319144	Protein-coding gene. Function not known.
UPP2	Uridine Phosphorylase 2	2	rs80319144	Catalyzes the reversible phosphorylytic cleavage of uridine and deoxyuridine to uracil and ribose- or deoxyribose-1-phosphate.

TANC1	Tetratricopeptide Repeat, Ankyrin Repeat And Coiled-Coil Containing 1	2	rs80319144	TANC1 is important for dendritic spine maintenance and spatial memory, and implicated in embryonic development. May be a scaffold component in the postsynaptic density (By similarity). TANC1 antibody is used as a synaptic marker. Regulates dendritic spines and excitatory synapses.
DAPL1	Death Associated Protein Like 1	2	rs80319144	Protein-coding gene. Function unknown. It was reported as a susceptibility locus for age-related macular degeneration.
CRBN	Cereblon	3	rs1848460	The protein binds to the intracellular C-terminus of the large conductance Ca(2+)-activated K(+)channel "Found in the cytoplasm localized with a calcium channel membrane protein, and is thought to play a role in brain development" - involved in human intelligence
SUMF1	Sulfatase modifying factor 1	3	rs1848460	The SUMF1 gene encodes an enzyme required for posttranslational modification and catalytic activation of the family of sulfatase enzymes.
CNTN4	Contactin 4	3	rs1848460	Contactins are axon-associated cell adhesion molecules that function in neuronal network formation and plasticity. The encoded protein is a glycosylphosphatidylinositol-anchored neuronal membrane protein that may play a role in the formation of axon connections in the developing nervous system.
LRRN1	Leucine rich repeat neuronal 1	3	rs1848460	A single-pass transmembrane proteins with conserved domain architecture. Lrrn1 may regulate the subcellular localisation of specific components of signalling or cell-cell recognition pathways in neuroepithelial cells in chick. It has the potential to regulate the subcellular trafficking of groups of functionally interacting molecules, such as receptors or ion channels, and could thereby regulate the integration of signalling pathways in NPCs during neural development.
TRNT1	tRNA nucleotidyl transferase, CCA-adding, 1	3	rs1848460	The CCA-adding enzyme TRNT1 is an essential enzyme that catalyzes the addition of the CCA terminus to the 3-prime end of tRNA precursors.
ATP2C1	ATPase, Ca++ Transporting, Type 2C, Member 1	3	rs35987657	This magnesium-dependent enzyme catalyzes the hydrolysis of ATP coupled with the transport of calcium ions. Defects in this gene cause Hailey-Hailey disease, an autosomal dominant disorder.
COL6A6	Collagen, type VI, alpha 6	3	rs35987657	COL6A6 gene is expressed in a wide range of fetal and adult tissues including lung, kidney, liver, spleen, thymus, heart and skeletal muscle
COL6A5	Collagen, type VI, alpha 5	3	rs35987657	COL6A6 gene is expressed in a wide range of fetal and adult tissues including kidney, salivary glands, intestinal tract and gallbladder.
NEK11	NIMA-related kinase 11	3	rs35987657	This gene encodes a member of the never in mitosis gene A family of kinases. The encoded protein localizes to the nucleoli, and may function with NEK2A in the S-phase checkpoint. The encoded protein appears to play roles in DNA replication and response to genotoxic stress.
CPNE4	Copine IV	3	rs35987657	This gene belongs to the highly conserved copine family. It encodes a calcium-dependent, phospholipid-binding protein, which may be involved in membrane trafficking, mitogenesis and development. Expressed in all brain regions.
COL6A5	Collagen, Type VI, Alpha 5	3	rs35987657	The encoded protein contains multiple von Willebrand factor A-like domains and may interact with the alpha 1 and alpha 2 chains of collagen VI to form the complete collagen VI trimer. Polymorphisms in this gene may be linked to dermal phenotypes, such as eczema.
CHST13	Carbohydrate (Chondroitin 4) Sulfotransferase 13	3	rs35987657	Catalyzes the transfer of sulfate to position 4 of the N-acetylgalactosamine (GalNAc) residue of chondroitin. Chondroitin sulfate constitutes the predominant proteoglycan present in cartilage.
UROC1	Urocanate Hydratase 1	3	rs35987657	This gene encodes an enzyme involved in histidine catabolism, metabolizing urocanic acid to formiminoglutamic acid. The gene product is known to protect the skin from ultra violet rays and is contained in human sweat.
PIK3R4	Phosphoinositide-3-Kinase, Regulatory Subunit 4	3	rs35987657	Regulatory subunit of the PI3K complex. May regulate membrane trafficking late in the endocytic pathway --> possible endocytosis function
ASTE1	Asteroid Homolog 1 (Drosophila)	3	rs35987657	Protein-coding gene. Possible role in EGF receptor signaling.
MDGA1	MAM Domain Containing Glycosylphosphatidylinositol Anchor 1	6	rs17636328	Required for radial migration of cortical neurons in the superficial layer of the neocortex (By similarity). Plays a role in the formation or maintenance of inhibitory synapses. May function by inhibiting the activity of NLGN2.
RNF8	Ring Finger Protein 8	6	rs17636328	The protein encoded by this gene contains a RING finger motif and an FHA domain. This protein has been shown to interact with several class II ubiquitin-conjugating enzymes (E2), including UBE2E1/UBCH6, UBE2E2, and

				UBE2E3, and may act as an ubiquitin ligase (E3) in the ubiquitination of certain nuclear proteins. This protein is also known to play a role in the DNA damage response and depletion of this protein causes cell growth inhibition and cell cycle arrest. Alternative splicing results in multiple transcript variants.
CCDC167	Coiled-Coil Domain Containing 167	6	rs17636328	Protein-coding gene. Function unknown.
GLO1	Glyoxalase 1	6	rs61192259	The enzyme encoded by this gene is responsible for the catalysis and formation of S-lactoyl-glutathione from methylglyoxal condensation and reduced glutatione.
ZNF804B	Zinc finger protein 804B	7	rs10952927	Candidate for anorexia nervosa. Zinc finger protein of unknown function.
ADAM22	ADAM Metallopeptidase Domain 22	7	rs10952927	Members of this family are membrane-anchored proteins, having been implicated in a variety of biological processes involving cell-cell and cell-matrix interactions, including fertilization, muscle development, and neurogenesis. This gene is highly expressed in the brain and may function as an integrin ligand in the brain. In mice, it has been shown to be essential for correct myelination in the peripheral nervous system.
STEAP4	STEAP Family Member 4	7	rs10952927	The protein encoded by this gene belongs to the STEAP (six transmembrane epithelial antigen of prostate) family, and resides in the golgi apparatus. It functions as a metalloreductase that has the ability to reduce both Fe(3+) to Fe(2+) and Cu(2+) to Cu(1+), using NAD(+) as acceptor. Important factor in metal uptake into the cells.
DACH1	Dachshund Family Transcription Factor 1	13	rs340561	This gene encodes a chromatin-associated protein that associates with other DNA-binding transcription factors to regulate gene expression and cell fate determination during development. The protein contains a Ski domain that is highly conserved from Drosophila to human. Expression of this gene is lost in some forms of metastatic cancer, and is correlated with poor prognosis. Multiple transcript variants encoding different isoforms have been found for this gene.
DIS3	DIS3 Homolog, Exosome Endoribonuclease And 3'-5' Exoribonuclease	13	rs340561	Putative catalytic component of the RNA exosome complex which has 3->5 exoribonuclease activity and participates in a multitude of cellular RNA processing and degradation events. DIS3 has both 3-5 exonuclease and endonuclease activities.
DPH6	Diphthamine Biosynthesis 6	15	rs996064	Amidase that catalyzes the last step of diphthamide biosynthesis using ammonium and ATP. Diphthamide biosynthesis consists in the conversion of an L-histidine residue in the translation elongation factor (EEF2) to diphthamide (By similarity)
MEIS2	Meis Homeobox 2	15	rs996064	Obviously, similar to Meis1. Involved in transcriptional regulation. Proposed to be involved in the transcriptional activation of EPHA8 in the developing midbrain.
SEMA6D	Sema domain, transmembrane domain (TM), and cytoplasmic domain, (semaphorin) 6D	15	rs111652004	Semaphorins are a large family, many of which have been implicated as inhibitors or chemorepellents in axon pathfinding, fasciculation and branching, and target selection. Possible role in the development of spinal cord and the sensory axons. Also plays a role in synaptic formation. Acts as a "gate-keeper" between central and peripheral nervous systems.
AAGAB	Alpha- And Gamma-Adaptin Binding Protein	15	rs868036	The protein encoded by this gene interacts with the gamma-adaptin and alpha-adaptin subunits of complexes involved in clathrin-coated vesicle trafficking. May be involved in endocytic recycling of growth factor receptors such as EGFR.
C15orf61	Chromosome 15 Open Reading Frame 61	15	rs868036	Protein-coding gene. Function not known.
CALML4	Calmodulin-Like 4	15	rs868036	Protein-coding gene. Function not known. Associated with breast cancer.
CLN6	Ceroid-Lipofuscinosis, Neuronal 6, Late Infantile, Variant	15	rs868036	This gene is one of eight which have been associated with neuronal ceroid lipofuscinoses. Likely encodes proteins involved in the degradation of post-translationally modified proteins in lysosomes
IQCH	IQ Motif Containing H	15	rs868036	Protein-coding gene. May play a regulatory role in spermatogenesis
MAP2K5	Mitogen-Activated Protein Kinase Kinase 5	15	rs868036	The protein encoded by this gene is a dual specificity protein kinase that belongs to the MAP kinase kinase family. The signal cascade mediated by this kinase is involved in growth factor stimulated cell proliferation and muscle cell differentiation

PIAS1	Protein Inhibitor Of Activated STAT, 1	15	rs868036	It inhibits STAT1-mediated gene activation and the DNA binding activity, binds to Gu protein/RNA helicase II/DEAD box polypeptide 21, and interacts with androgen receptor (AR). It functions in testis as a nuclear receptor transcriptional coregulator and may have a role in AR initiation and maintenance of spermatogenesis.
SKOR1	SKI Family Transcriptional Corepressor 1	15	rs868036	Function not well known. Acts as a transcriptional corepressor of LBX1 (By similarity). Could inhibit BMP signaling.
SMAD3	SMAD Family Member 3	15	rs868036	The protein encoded by this gene belongs to the SMAD, a family of proteins similar to the gene products of the Drosophila gene 'mothers against decapentaplegic' (Mad) and the C. elegans gene Sma. SMAD proteins are signal transducers and transcriptional modulators that mediate multiple signaling pathways. This protein functions as a transcriptional modulator activated by transforming growth factor-beta and is thought to play a role in the regulation of carcinogenesis.
PRAC	Prostate Cancer Susceptibility Candidate 1	17	rs12450895	This gene is reported to be specifically expressed in prostate, rectum and distal colon. Sequence analysis suggests that it may play a regulatory role in the nucleus.
SETBP1	SET binding protein 1	18	rs12962305	The encoded protein has been shown to bind the SET nuclear oncogene which is involved in DNA replication. Mutations in this gene are associated with Schinzel-Giedion midface retraction syndrome.
SLC14A2	Solute Carrier Family 14 (Urea Transporter), Member 2	18	rs12962305	This protein is expressed in the inner medulla of the kidney, and mediates rapid transepithelial urea transport across the inner medullary collecting duct.
MYT1	Myelin Transcription Factor 1	20	rs365032	Neural specific, zinc finger-containing DNA-binding protein. The protein binds to the promoter regions of proteolipid proteins of the central nervous system and plays a role in the developing nervous system. Regulates neural transcription and is expressed in early stages of neural differentiation.
PPDPF	Pancreatic Progenitor Cell Differentiation And Proliferation Factor	20	rs365032	Probable regulator of exocrine pancreas development (by similarity).
PTK6	Protein Tyrosine Kinase 6	20	rs365032	Non-receptor tyrosine-protein kinase implicated in the regulation of a variety of signaling pathways that control the differentiation and maintenance of normal epithelia, as well as tumor growth. Function seems to be context dependent and differ depending on cell type, as well as its intracellular localization.
ABHD16B	Abhydrolase Domain Containing 16B	20	rs365032	Not much information available. A protein-coding gene. GO annotations related to this gene include hydrolase activity.
TPD52L2	Tumor Protein D52-Like 2	20	rs365032	This gene encodes a member of the tumor protein D52-like family. Expression of this gene may be a marker for breast cancer and acute lymphoblastic leukemia.
UCKL1	Uridine-Cytidine Kinase 1-Like 1	20	rs365032	GO annotations related to this gene include uridine kinase activity and phosphotransferase activity, alcohol group as acceptor. May contribute to UTP accumulation needed for blast transformation and proliferation.
DNAJC5	DnaJ (Hsp40) Homolog, Subfamily C, Member 5	20	rs365032	This gene is a member of the J protein family. The encoded protein plays a role in membrane trafficking and protein folding, and has been shown to have anti-neurodegenerative properties. The encoded protein is known to play a role in cystic fibrosis and Huntington's disease.
OPRL1	Opiate receptor-like 1	20	rs365032	G-protein coupled opioid receptor that functions as receptor for the endogenous neuropeptide nociceptin. Plays a role in modulating nociception and the perception of pain. Plays a role in the regulation of locomotor activity by the neuropeptide nociceptin.

Supplementary Table 7: Results of gene set (pathway) enrichment analysis using BI-ENRICH. Gene set, predefined gene set; the prefix UNIKB represents the category defined by UniProtKB, GO by Gene ontology, and the others from GSEA curated data set; z, enrichment z-score corresponding to the p value of one-side Fisher test; total.gene, total gene number in a gene set; genes, number of genes in the intersection between a gene set and the candidate genes; gene list, acronyms of the genes in the intersection between a gene set and the candidate genes; p, empirical p value based on phenotype label permutation; FDR, adjusted q-value (Benjamini-Hochberg) based on phenotype label permutation; sampling-p-value, empirical p value determined by random resampling of pseudo candidate gene sets; sampling-FDR, empirical false discovery rate determined by random resampling of pseudo candidate gene sets. *GO_DNA_REPAIR also represents genesets GO_DOUBLE_STRAND_BREAK_REPAIR and UNIKB_DNA_damage; **GO_IMMUNE_SYSTEM_DEVELOPMENT also represents gene set GO_MYELOID_CELL_DIFFERENTIATION.

Gene set	z	total.gene	genes	Gene list	p	FDR	sampling-p-value	sampling-FDR	Shown in figure 4
GO_LOCOMOTORY_BEHAVIOR	5·690	181	4	*BTBD9 CLN6 HOXB8 MEIS1*	0·002	0·029	3·04E-07	0	Y
GO_DNA_REPAIR	4·978	480	4	*APLF ASTE1 PRMT6 RNF8*	0·005	0·036	1·48E-04	0·01	Y*
UNIKB_Neurogenesis	5·481	244	4	*MDGA1 MYT1 NTNG1 SEMA6D*	0·005	0·036	2·69E-06	0	Y
GO_DOUBLE_STRAND_BREAK_REPAIR	3·904	165	2	*APLF RNF8*	0·009	0·060	2·87E-03	0·649	N*
GO_NUCLEIC_ACID_PHOSPHODIESTER_BOND_HYDROLYSIS	4·648	254	3	*APLF ASTE1 DIS3*	0·010	0·061	2·12E-05	0	Y
GO_PROTEIN_DNA_COMPLEX_SUBUNIT_ORGANIZATION	3·742	229	2	*DACH1 RNF8*	0·011	0·063	4·99E-03	0·716	Y
GO_IMMUNE_SYSTEM_DEVELOPMENT	5·460	582	5	*GLO1 HOXB3 MEIS1 RNF8 SMAD3*	0·013	0·070	3·61E-04	0·029	Y**
GO_NEGATIVE_REGULATION_OF_TRANSCRIPTION_ FROM_RNA_POLYMERASE_II_PROMOTER	5·242	740	5	*DACH1 HOXB3 PRMT6 RNF8 SMAD3*	0·016	0·077	6·87E-04	0·148	Y
GO_REGULATION_OF_DNA_METABOLIC_PROCESS	4·463	340	3	*APLF DACH1 RNF8*	0·017	0·077	3·39E-04	0·028	Y
GO_MYELOID_CELL_DIFFERENTIATION	3·837	189	2	*GLO1 MEIS1*	0·017	0·077	1·75E-04	0·011	N**
GO_BEHAVIOR	5·566	516	5	*BTBD9 CLN6 DACH1 HOXB8 MEIS1*	0·019	0·082	1·22E-04	0·007	Y
REACTOME_POST_TRANSLATIONAL_PROTEIN_MODIFICATION	3·840	188	2	*SEMA6D SUMF1*	0·020	0·082	2·06E-03	0·585	Y
UNIKB_DNA_damage	4·479	332	3	*APLF PRMT6 RNF8*	0·022	0·086	6·95E-05	0·002	N*
GO_POSITIVE_REGULATION_OF_CELL_MORPHOGENESIS_ INVOLVED_IN_DIFFERENTIATION	3·913	162	2	*PTPRD SMAD3*	0·023	0·086	4·47E-03	0·706	Y
GO_NEGATIVE_REGULATION_OF_CELL_GROWTH	3·889	170	2	*SEMA6D SMAD3*	0·024	0·086	8·77E-05	0·004	Y
KEGG_AXON_GUIDANCE	4·022	129	2	*NTNG1 SEMA6D*	0·025	0·086	9·02E-05	0·004	Y
GO_SKELETAL_SYSTEM_DEVELOPMENT	5·019	455	4	*HOXB3 HOXB8 MEIS1 SMAD3*	0·031	0·103	1·59E-04	0·01	
GO_CELL_JUNCTION_ORGANIZATION	3·848	185	2	*PKP4 SMAD3*	0·037	0·118	1·40E-05	0	
GO_REGULATION_OF_CELL_MORPHOGENESIS_INVOLVED_ IN_DIFFERENTIATION	4·469	337	3	*PTPRD SEMA6D SMAD3*	0·042	0·128	1·80E-03	0·569	
GO_NEGATIVE_REGULATION_OF_NEURON_DIFFERENTIATION	3·832	191	2	*MEIS1 SEMA6D*	0·043	0·128	3·81E-03	0·69	
GO_NEGATIVE_REGULATION_OF_CELL_DIFFERENTIATION	4·790	609	4	*HOXB8 MEIS1 SEMA6D SMAD3*	0·046	0·132	4·44E-04	0·034	

GO_COVALENT_CHROMATIN_MODIFICATION	3·530	345	2	*PRMT6 RNF8*	0·053	0·147	7·64E-03	0·796
GO_PROTEIN_CATABOLIC_PROCESS	4·108	579	3	*CLN6 CRBN RNF8*	0·060	0·161	6·64E-03	0·764
GO_NEGATIVE_REGULATION_OF_LOCOMOTION	3·671	263	2	*DACH1 SEMA6D*	0·069	0·170	4·52E-03	0·707
GO_LYMPHOCYTE_ACTIVATION	3·535	342	2	*RNF8 SMAD3*	0·069	0·170	6·21E-03	0·75
GO_REGULATION_OF_BINDING	3·634	283	2	*CRBN SMAD3*	0·070	0·170	2·51E-03	0·631
GO_POSITIVE_REGULATION_OF_PROTEOLYSIS	3·503	363	2	*CLN6 SMAD3*	0·071	0·170	4·29E-03	0·702
GO_MACROMOLECULE_CATABOLIC_PROCESS	4·441	926	4	*CLN6 CRBN DIS3 RNF8*	0·075	0·172	4·79E-03	0·71
GO_REGULATION_OF_CELL_DEVELOPMENT	5·128	836	5	*HOXB3 MEIS1 PTPRD SEMA6D SMAD3*	0·076	0·172	9·41E-04	0·304
GO_NCRNA_PROCESSING	3·470	386	2	*DIS3 SMAD3*	0·078	0·172	7·96E-03	0·819
GO_SENSORY_PERCEPTION	3·759	938	3	*BTBD9 CLN6 HOXB8*	0·108	0·232	1·09E-02	1
GO_REGULATION_OF_NERVOUS_SYSTEM_DEVELOPMENT	4·620	750	4	*HOXB3 MEIS1 PTPRD SEMA6D*	0·116	0·243	2·61E-03	0·635
GO_CELL_CELL_ADHESION	4·073	608	3	*PKP4 PTPRD SMAD3*	0·122	0·250	3·10E-03	0·674
UNIKB_Transcription	5·273	2341	8	*DACH1 HOXB3 HOXB8 MEIS1 MYT1 PRMT6 SMAD3 TOX3*	0·125	0·250	4·82E-03	0·711
GO_REGULATION_OF_NEURON_DIFFERENTIATION	4·138	554	3	*MEIS1 PTPRD SEMA6D*	0·128	0·250	3·30E-03	0·678
GO_REGULATION_OF_TRANSCRIPTION_FROM_ RNA_POLYMERASE_II_PROMOTER	5·260	1784	7	*DACH1 GLO1 HOXB3 MEIS1 PRMT6 RNF8 SMAD3*	0·133	0·254	2·12E-03	0·595
GO_REGULATION_OF_CYTOPLASMIC_TRANSPORT	3·350	481	2	*BTBD9 SMAD3*	0·151	0·272	8·52E-03	1
GO_REGULATION_OF_MULTICELLULAR_ ORGANISMAL_DEVELOPMENT	5·343	1672	7	*APLF HOXB3 HOXB8 MEIS1 PTPRD SEMA6D SMAD3*	0·152	0·272	2·09E-03	0·595
GO_REGULATION_OF_CELL_CYCLE_PROCESS	3·267	558	2	*DACH1 PKP4*	0·152	0·272	1·51E-02	1
GO_GROWTH	3·438	410	2	*PRMT6 SMAD3*	0·158	0·272	1·04E-02	1
GO_REGULATION_OF_CELL_DIFFERENTIATION	5·039	1492	6	*HOXB3 HOXB8 MEIS1 PTPRD SEMA6D SMAD3*	0·159	0·272	2·14E-03	0·597
GO_REGULATION_OF_IMMUNE_SYSTEM_PROCESS	4·069	1403	4	*APLF HOXB8 MEIS1 SMAD3*	0·164	0·272	1·18E-02	1
GO_NEUROGENESIS	5·110	1402	6	*HOXB3 MDGA1 MEIS1 NTNG1 PTPRD SEMA6D*	0·168	0·272	2·71E-03	0·638
GO_REGULATION_OF_NEURON_PROJECTION_DEVELOPMENT	3·440	408	2	*PTPRD SEMA6D*	0·169	0·272	7·54E-03	0·795
GO_ORGANIC_HYDROXY_COMPOUND_METABOLIC_PROCESS	3·349	482	2	*BTBD9 CLN6*	0·171	0·272	4·56E-03	0·708
GO_REGULATION_OF_CELL_CYCLE	3·751	949	3	*DACH1 PKP4 SMAD3*	0·180	0·278	1·18E-02	1
GO_CENTRAL_NERVOUS_SYSTEM_DEVELOPMENT	4·493	872	4	*HOXB3 HOXB8 MDGA1 SEMA6D*	0·187	0·278	3·36E-03	0·678
GO_POSITIVE_REGULATION_OF_HYDROLASE_ACTIVITY	3·786	905	3	*DIS3 PKP4 SMAD3*	0·189	0·278	6·89E-03	0·771

GO_CELLULAR_AMIDE_METABOLIC_PROCESS	3·114	727	2	*CLN6 GLO1*	0·189	0·278	2·12E-02	1
GO_PROTEIN_UBIQUITINATION	3·198	629	2	*CRBN RNF8*	0·191	0·278	2·09E-02	1
GO_REGULATION_OF_CELLULAR_COMPONENT_MOVEMENT	3·904	771	3	*DACH1 SEMA6D SMAD3*	0·195	0·280	6·36E-03	0·756
GO_BIOLOGICAL_ADHESION	4·347	1032	4	*COL6A6 PKP4 PTPRD SMAD3*	0·200	0·282	3·19E-03	0·676
UNIKB_Cell_adhesion	3·368	466	2	*COL6A6 PKP4*	0·213	0·289	4·29E-03	0·702
GO_CATABOLIC_PROCESS	4·366	1773	5	*CLN6 COL6A6 CRBN DIS3 RNF8*	0·214	0·289	1·03E-02	1
GO_HEAD_DEVELOPMENT	3·965	709	3	*HOXB3 MDGA1 SEMA6D*	0·215	0·289	6·12E-03	0·75
GO_NEGATIVE_REGULATION_OF_CELL_DEATH	3·814	872	3	*GLO1 SMAD3 TOX3*	0·225	0·298	7·95E-03	0·819
GO_REGULATION_OF_IMMUNE_RESPONSE	3·015	858	2	*APLF SMAD3*	0·231	0·301	2·85E-02	1
GO_NEGATIVE_REGULATION_OF_ CELLULAR_COMPONENT_ORGANIZATION	3·150	684	2	*CRBN SEMA6D*	0·237	0·303	2·69E-02	1
GO_PROTEOLYSIS	3·565	1208	3	*CRBN RNF8 SMAD3*	0·241	0·303	2·30E-02	1
GO_CARDIOVASCULAR_SYSTEM_DEVELOPMENT	3·888	788	3	*HOXB3 MEIS1 SMAD3*	0·247	0·303	9·10E-03	1
GO_CIRCULATORY_SYSTEM_DEVELOPMENT	3·888	788	3	*HOXB3 MEIS1 SMAD3*	0·247	0·303	9·10E-03	1
GO_CELL_DEVELOPMENT	4·599	1426	5	*MEIS1 NTNG1 RNF8 SEMA6D SMAD3*	0·257	0·311	5·17E-03	0·723
GO_REGULATION_OF_GTPASE_ACTIVITY	3·159	673	2	*DIS3 PKP4*	0·263	0·314	1·77E-02	1
NABA_MATRISOME	3·690	1028	3	*COL6A6 NTNG1 SEMA6D*	0·268	0·316	1·10E-02	1
GO_NEGATIVE_REGULATION_OF_CELL_PROLIFERATION	3·186	643	2	*DACH1 SMAD3*	0·311	0·361	2·00E-02	1
GO_NEURON_DIFFERENTIATION	3·812	874	3	*MDGA1 NTNG1 PTPRD*	0·318	0·365	1·14E-02	1
GO_ORGANONITROGEN_COMPOUND_METABOLIC_PROCESS	3·832	1796	4	*BTBD9 CLN6 GLO1 SUMF1*	0·327	0·367	1·44E-02	1
GO_TRANSCRIPTION_FROM_RNA_POLYMERASE_II_PROMOTER	3·116	724	2	*DACH1 MEIS1*	0·329	0·367	2·78E-02	1
GO_CELL_CELL_SIGNALING	3·082	767	2	*PKP4 PTPRD*	0·336	0·370	2·47E-02	1
GO_LOCOMOTION	3·628	1114	3	*CLN6 MDGA1 SEMA6D*	0·346	0·377	1·70E-02	1
GO_POSITIVE_REGULATION_OF_MOLECULAR_FUNCTION	3·835	1791	4	*CRBN DIS3 PKP4 SMAD3*	0·367	0·395	1·72E-02	1
GO_CELL_DEATH	2·920	1001	2	*SMAD3 TOX3*	0·411	0·436	4·67E-02	1
GO_CELL_MOTILITY	3·031	835	2	*MDGA1 SEMA6D*	0·429	0·445	3·05E-02	1
GO_LOCALIZATION_OF_CELL	3·031	835	2	*MDGA1 SEMA6D*	0·429	0·445	3·05E-02	1
GO_POSITIVE_REGULATION_OF_GENE_EXPRESSION	3·270	1733	3	*MEIS1 SMAD3 TOX3*	0·494	0·502	4·81E-02	1
GO_TISSUE_DEVELOPMENT	3·381	1518	3	*HOXB3 SEMA6D SMAD3*	0·498	0·502	3·68E-02	1
GO_REGULATION_OF_TRANSPORT	3·236	1804	3	*BTBD9 CRBN SMAD3*	0·502	0·502	4·83E-02	1

Supplementary Table 8: Results of gene prioritization analysis using BI-ENRICH. p, empirical p value based on phenotype label permutation; FDR, adjusted q-value (Benjamini-Hochberg) based on phenotype label permutation; Locus ID, ID of the 20 independent signals in the present meta-analysis on RLS; Overlap, indicates that the gene is located within the genomic region of the locus defined by PLINK clumping (see "Identification of genome-wide significant SNPs in Supplementary Methods") and is "NA" if no overlap; eQTL, eQTL info, including rs-ID of the SNP, p value in meta-analysis (less than 1e-5), minimal eQTL p value, and corresponding tissue; Enhancer, gene is located within a distance of 1 Mb from an enhancer annotated by VISTA or ROADMAP.

Gene	p	FDR	Locus lead SNP rsID	Overlap (locus lead SNP rsID)	eQTL (rsid; rlspvalue; eQTLpvalue; eQTLtissue)	Enhancer
PKP4	3·4E-05	0·0018	rs80319144	rs80319144	NA	NA
MEIS1	2·7E-04	0·0070	rs113851554	rs113851554	rs10188003;1·161e-24;9·577861e-07;Brain_Caudate_basal_ganglia; rs10194077;9·979e-06;9·577861e-07;Brain_Caudate_basal_ganglia; rs10196555;3·32e-07;9·277834e-09;Cells_Transformed_fibroblasts; rs11897119;9·572e-06;9·577861e-07;Brain_Caudate_basal_ganglia; rs12713581;3·311e-07;9·284326e-09;Cells_Transformed_fibroblasts; rs13029520;1·754e-24;9·577861e-07;Brain_Caudate_basal_ganglia; rs2300481;2·474e-24;1·481635e-06;Brain_Caudate_basal_ganglia; rs3891585;4·126e-11;3·162709e-06;Brain_Caudate_basal_ganglia; rs4233937;2·678e-11;2·181349e-06;Brain_Caudate_basal_ganglia; rs7603236;6·138e-11;3·149119e-06;Brain_Caudate_basal_ganglia;	NA
HOXB8	1·2E-03	0·0152	rs12450895	rs12450895	NA	NA
MYT1	1·1E-03	0·0152	rs365032	rs365032	NA	NA
SEMA6D	2·2E-03	0·0231	rs111652004	rs111652004	NA	NA
MDGA1	2·9E-03	0·0250	rs17636328	NA	rs11752456;2·374e-13;1·295993e-05;Adipose_Subcutaneous; rs1738456;4·041e-08;2·192363e-08;Esophagus_Mucosa; rs4714101;5·567e-06;1·11428e-07;Whole_Blood; rs708020;1·969e-08;2·200614e-08;Esophagus_Mucosa; rs708022;8·775e-09;2·19565e-08;Esophagus_Mucosa;rs804816;2·39e-08;5·001761e08;Skin_Sun_Exposed_Lower_leg; rs9394447;6·841e-10;1·030476e-06;Brain_Cerebellum;	NA
PTPRD	5·6E-03	0·0333	rs1836229, rs62535767	rs1836229, rs62535767	NA	NA
SMAD3	5·8E-03	0·0333	rs868036	rs868036	NA	NA
BTBD9	5·3E-03	0·0333	rs61192259	rs61192259	rs10947726;2·142e-19;1·507626e-07;Muscle_Skeletal; rs10947728;8·884e-22;4·420247e-06;Muscle_Skeletal; rs111624546;3·167e-08;6·973475e-06;Muscle_Skeletal; rs115847695;2·626e-07;5·43518e-06;Muscle_Skeletal; rs12201631;1·492e-20;4·28422e-06;Muscle_Skeletal; rs12208912;7·368e-20;6·377098e-07;Muscle_Skeletal; rs12212723;7·037e-06;9·082119e-06;Muscle_Skeletal; rs12212983;2·4e-16;2·161695e-06;Muscle_Skeletal; rs12215850;9·742e-20;2·912857e-07;Muscle_Skeletal; rs17543178;6·936e-08;6·615696e-07;Muscle_Skeletal; rs17544798;9·011e-08;4·959167e-07;Muscle_Skeletal; rs28445226;1·802e-19;2·06387e-07;Muscle_Skeletal; rs55835070;1·429e-08;5·976503e-07;Muscle_Skeletal; rs57180089;8·02e-09;6·005257e-07;Muscle_Skeletal; rs73733937;1·396e-08;6·092468e-07;Muscle_Skeletal; rs73733941;1·399e-08;6·092712e-07;Muscle_Skeletal; rs73733942;1·399e-08;6·083656e-07;Muscle_Skeletal; rs73733958;1·255e-08;6·134498e-07;Muscle_Skeletal; rs73733994;1·546e-08;5·799496e-07;Muscle_Skeletal; rs73733996;1·938e-08;5·990985e-07;Muscle_Skeletal; rs73734417;6·261e-07;2·804732e-06;Muscle_Skeletal; rs75271174;1·638e-08;5·973147e-07;Muscle_Skeletal; rs75705800;3·003e-06;1·820336e-08;Muscle_Skeletal; rs78293217;1·639e-08;1·488303e-06;Muscle_Skeletal;	NA
NTNG1	1·0E-02	0·0425	rs12046503	NA	NA	Enh
DIS3	1·1E-02	0·0425	rs340561	NA	NA	Enh
CLN6	9·9E-03	0·0425	rs868036	rs868036	rs7177443;1·002e-07;1·358184e-05;Nerve_Tibial;	NA
HOXB9	9·0E-03	0·0425	rs12450895	rs12450895	NA	NA
HOXB7	1·4E-02	0·0466	rs12450895	rs12450895	rs12952262;2·492e-06;1·954671e-06;Adipose_Subcutaneous; rs2303488;5·042e-06;1·052736e-05;Adipose_Subcutaneous; rs66459980;4·95e-06;1·712926e-05;Adipose_Subcutaneous; rs8066740;2·04e-06;2·541822e-06;Adipose_Subcutaneous;	NA

APLF	1·4E-02	0·0466	rs1820989	NA	rs11126167;2·941e-06;1·1579e-06;Uterus; rs1156860;3·112e-06;7·960463e-07;Uterus; rs13000088;2·933e-06;1·158432e-06;Uterus; rs13415993;2·35e-06;1·667236e-06;Uterus; rs1503239;1·591e-06;1·723186e-06;Uterus; rs34036669;1·543e-06;1·629415e-06;Uterus;	NA
GLO1	1·3E-02	0·0466	rs61192259	rs61192259	rs10484854;4·274e-06;3·893686e-06;Muscle_Skeletal; rs10807196;4·959e-28;1·193406e-08;Artery_Tibial; rs10807198;2·322e-14;1·654273e-07;Thyroid; rs11961189;5·339e-16;6·875747e-06;Thyroid; rs11966098;2·277e-14;2·797122e-06;Artery_Tibial; rs12209475;2·927e-06;1·023343e-08;Lung; rs12209477;3·686e-06;2·031443e-07;Thyroid; rs12210866;2·962e-07;1·291331e-08;Lung; rs13200763;1·695e-07;1·034852e-08;Lung; rs13213477;3·696e-13;1·014761e-05;Esophagus_Muscularis; rs1626200;9·514e-11;1·998806e-05;Nerve_Tibial; rs1699005;3·211e-14;1·620903e-07;Thyroid; rs1739625;5·902e-07;4·704299e-07;Muscle_Skeletal; rs1739627;3·642e-14;1·050612e-05;Esophagus_Muscularis; rs1739630;1·977e-14;1·539803e-06;Artery_Aorta; rs1739632;3·745e-14;1·7255e-08;Thyroid; rs1781715;2·873e-11;6·658268e-07;Artery_Tibial; rs1781717;4·61e-07;1·127247e-08;Esophagus_Muscularis; rs1781719;4·373e-07;1·208098e-08;Esophagus_Muscularis; rs1781735;9·451e-07;1·077137e-18;Muscle_Skeletal; rs1781739;8·089e-07;1·094296e-06;Muscle_Skeletal; rs1781741;8·359e-07;6·087026e-07;Muscle_Skeletal; rs1997949;4·02e-06;8·442933e-07;Muscle_Skeletal; rs2092797;4·079e-10;2·949301e-06;Thyroid; rs2144328;9·383e-14;1·774968e-08;Thyroid; rs2180104;5·915e-06;2·79949e-08;Artery_Tibial; rs2471999;5·753e-11;1·473275e-08;Artery_Tibial; rs2474245;2·245e-10;3·300084e-08;Artery_Tibial; rs2490026;6·308e-09;1·18643e-07;Thyroid; rs2748157;6·859e-09;3·003696e-09;Artery_Tibial; rs2748164;2·136e-10;1·534458e-05;Esophagus_Muscularis; rs2748165;5·125e-15;1·116469e-08;Artery_Tibial; rs2748166;5·597e-07;5·191831e-07;Muscle_Skeletal; rs2748172;8·718e-06;2·939795e-07;Thyroid; rs2814883;6·237e-15;4·129588e-07;Thyroid; rs2814885;7·645e-06;1·773129e-08;Artery_Tibial; rs2814889;6·704e-15;1·736453e-08;Artery_Tibial; rs2814893;3·933e-07;3·37773e-07;Muscle_Skeletal; rs2814894;5·332e-14;1·675374e-08;Thyroid; rs34871682;6·763e-25;1·643218e-06;Thyroid; rs4546477;3·436e-15;2·310758e-06;Artery_Tibial; rs4599628;2·011e-12;2·944908e-06;Artery_Tibial; rs4629680;1·876e-16;1·568435e-05;Thyroid; rs4714160;1·174e-37;1·975261e-05;Thyroid; rs4714162;3·495e-39;1·49467e-05;Thyroid; rs4714175;3·792e-11;1·473275e-08;Artery_Tibial; rs61661697;2·584e-12;1·012054e-07;Artery_Tibial; rs6458065;4·464e-12;1·127182e-05;Esophagus_Muscularis; rs66961365;1·249e-14;5·475972e-06;Esophagus_Muscularis; rs6932235;4·484e-10;2·165118e-06;Thyroid; rs73414350;3·498e-14;2·206793e-06;Artery_Tibial; rs7739414;5·945e-15;2·310956e-06;Artery_Tibial; rs7750172;6·415e-15;2·301276e-06;Artery_Tibial; rs7774816;4·569e-15;2·349351e-06;Artery_Tibial; rs910516;3·455e-14;2·24696e-08;Artery_Tibial; rs9366973;2·027e-10;4·704954e-06;Thyroid; rs9369062;2·688e-50;4·20999e-06;Thyroid; rs9380766;6·801e-15;2·324863e-06;Artery_Tibial;	NA
HOXB2	1·9E-02	0·0548	rs12450895	NA	rs12450895;4·884e-08;1·718022e-06;Cells_Transformed_fibroblasts; rs12453466;1·232e-07;1·723669e-06;Cells_Transformed_fibroblasts; rs12952262;2·492e-06;1·075997e-09;Lung; rs2303488;5·042e-06;1·055306e-10;Adipose_Subcutaneous; rs2326016;9·596e-07;1·684382e-07;Adipose_Subcutaneous; rs2326017;2·53e-06;2·722149e-06;Thyroid; rs28575348;4·95e-06;1·158905e-09;Cells_Transformed_fibroblasts; rs3096644;3·612e-06;1·103066e-06;Adipose_Subcutaneous; rs3744772;3·082e-06;1·653765e-06;Cells_Transformed_fibroblasts; rs4793943;6·466e-06;1·027271e-07;Adipose_Visceral_Omentum; rs58702553;5·329e-06;1·003943e-06;Whole_Blood; rs66459980;4·95e-06;1·096829e-14;Lung; rs71366902;4·858e-06;1·202501e-07;Thyroid; rs8066740;2·04e-06;1·091478e-10;Adipose_Subcutaneous; rs872760;7·808e-06;1·091528e-12;Adipose_Subcutaneous; rs9896015;5·558e-06;1·580941e-08;Whole_Blood; rs9913026;6·505e-06;1·906023e-07;Adipose_Subcutaneous;	NA
HOXB3	1·8E-02	0·0548	rs12450895	rs12450895	rs12952262;2·492e-06;6·997766e-07;Muscle_Skeletal; rs4793943;6·466e-06;1·720341e-07;Muscle_Skeletal; rs66459980;4·95e-06;1·484331e-07;Muscle_Skeletal; rs872760;7·808e-06;1·53993e-07;Muscle_Skeletal;	NA
HOXB6	2·0E-02	0·0548	rs12450895	rs12450895	NA	NA
RNF8	2·3E-02	0·0593	rs17636328	NA	NA	Enh
HOXB5	3·0E-02	0·0741	rs12450895	rs12450895	NA	NA
PRMT6	3·5E-02	0·0789	rs12046503	NA	NA	Enh
ASTE1	3·5E-02	0·0789	rs35987657	NA	NA	Enh
DACH1	4·8E-02	0·1046	rs340561	NA	NA	Enh
TOX3	5·0E-02	0·1046	rs45544231	rs45544231	NA	NA
MAP2K5	5·6E-02	0·1115	rs868036	rs868036	rs10152422;1·492e-20;1·20642e-05;Skin_Sun_Exposed_Lower_leg; rs10152595;3·623e-06;1·170795e-10;Artery_Tibial; rs10152913;4·134e-08;1·363515e-06;Nerve_Tibial; rs1026731;9·292e-06;1·114565e-06;Adipose_Subcutaneous; rs1026733;8·476e-06;1·014896e-06;Adipose_Subcutaneous; rs1026734;7·586e-06;1·114565e-06;Adipose_Subcutaneous;	NA

	rs1026735;3·572e-16;1·114565e-06;Adipose_Subcutaneous; rs1026737;5·375e-13;1·232094e-05;Nerve_Tibial; rs10438355;9·5e-08;1·102002e-11;Artery_Tibial; rs10468041;3·596e-12;1·023678e-06;Skin_Sun_Exposed_Lower_leg; rs10518739;5·442e-18;2·704612e-08;Heart_Atrial_Appendage; rs10775194;5·989e-20;1·00205e-06;Esophagus_Muscularis; rs11071940;3·665e-08;2·201925e-11;Artery_Tibial; rs113203559;3·202e-08;1·363515e-06;Nerve_Tibial; rs11401837;2·296e-16;1·473338e-06;Thyroid; rs11451847;3·762e-18;1·023594e-14;Nerve_Tibial; rs11631200;1·025e-14;1·53938e-05;Thyroid; rs11637027;8·743e-10;1·239205e-06;Muscle_Skeletal; rs11637445;2·131e-15;1·884404e-07;Muscle_Skeletal; rs11638507;1·21e-12;1·880643e-07;Muscle_Skeletal; rs11854197;1·121e-16;1·51777e-05;Skin_Sun_Exposed_Lower_leg; rs11856740;1·307e-12;1·205385e-07;Muscle_Skeletal; rs11857017;1·499e-22;1·045757e-13;Whole_Blood; rs12148363;1·503e-08;1·076241e-10;Thyroid; rs12438118;3·039e-20;1·000149e-10;Adipose_Subcutaneous; rs12439895;2·321e-17;1·0432e-08;Artery_Aorta; rs12441823;7·864e-20;1·040636e-17;Artery_Tibial; rs12442323;4·109e-08;1·000858e-06;Lung; rs12591495;4·968e-08;1·348448e-06;Nerve_Tibial; rs12592101;9·351e-15;2·088743e-06;Heart_Atrial_Appendage; rs12592315;4·026e-15;3·0387e-06;Muscle_Skeletal; rs12593813;7·892e-17;1·842217e-07;Thyroid; rs12594229;4·241e-08;1·013836e-06;Lung; rs12594234;4·835e-08;1·012643e-06;Lung; rs12595334;1·279e-07;1·073452e-06;Artery_Aorta; rs12899051;4·385e-08;1·213094e-06;Nerve_Tibial; rs12900758;5·904e-08;1·412397e-08;Whole_Blood; rs12901789;2·897e-08;1·029685e-07;Whole_Blood; rs12902804;1·979e-17;1·074569e-05;Skin_Sun_Exposed_Lower_leg; rs12905509;7·015e-17;1·412032e-05;Skin_Sun_Exposed_Lower_leg; rs12910062;6·387e-08;1·16858e-06;Nerve_Tibial; rs12910196;5·201e-08;1·353692e-06;Nerve_Tibial; rs12911370;4·42e-08;1·218532e-06;Nerve_Tibial; rs12912974;6·81e-17;1·384927e-08;Heart_Atrial_Appendage; rs12915085;5·893e-08;1·539986e-09;Adipose_Subcutaneous; rs12915438;3·904e-16;1·088649e-10;Whole_Blood; rs12916403;3·837e-08;1·059961e-06;Lung; rs13329413;6·92e-29;1·225409e-10;Adipose_Subcutaneous; rs13329567;9·766e-13;2·198714e-09;Whole_Blood; rs1444935;3·999e-08;1·022605e-06;Lung; rs1478941;3·927e-12;1·007625e-05;Muscle_Skeletal; rs16950804;5·276e-08;1·218532e-06;Nerve_Tibial; rs16951001;2·486e-10;1·199283e-08;Heart_Atrial_Appendage; rs16951105;1·043e-20;1·035472e-06;Esophagus_Muscularis; rs16953610;6·23e-08;2·080878e-07;Esophagus_Muscularis; rs17241403;1·46e-12;1·553677e-07;Muscle_Skeletal; rs17242605;4·764e-17;1·549421e-05;Skin_Sun_Exposed_Lower_leg; rs17243334;5·409e-18;2·704612e-08;Heart_Atrial_Appendage; rs17526859;3·606e-08;1·363515e-06;Nerve_Tibial; rs1871307;3·062e-16;1·118382e-06;Adipose_Subcutaneous; rs201144707;6·464e-06;8·602205e-06;Whole_Blood; rs2033783;4·35e-08;1·678246e-07;Esophagus_Muscularis; rs2127163;1·434e-28;1·040824e-06;Lung; rs2166185;3·096e-08;1·543211e-06;Thyroid; rs2241422;7·641e-22;1·948411e-07;Heart_Atrial_Appendage; rs2289789;6·382e-08;1·353106e-06;Nerve_Tibial; rs2414952;1·279e-17;2·120417e-07;Ovary; rs28376010;1·593e-31;1·04331e-07;Thyroid; rs28376697;2·797e-24;1·126413e-08;Adipose_Subcutaneous; rs28418298;1·161e-20;1·618297e-12;Whole_Blood; rs28427879;1·575e-11;2·115887e-06;Ovary; rs28565266;8·838e-21;1·151135e-12;Artery_Tibial; rs28568344;1·574e-26;5·009745e-06;Heart_Atrial_Appendage; rs28645602;5·987e-08;1·353106e-06;Nerve_Tibial; rs28670272;8·628e-13;1·091756e-05;Nerve_Tibial; rs28696998;3·919e-08;1·00721e-06;Lung; rs28735117;3·495e-22;1·640317e-10;Adipose_Subcutaneous; rs28742003;1·108e-28;1·574074e-10;Artery_Tibial; rs2899730;1·548e-09;1·301987e-07;Adipose_Subcutaneous; rs34004236;2·952e-20;1·383029e-07;Heart_Left_Ventricle; rs34221449;4·136e-08;1·363515e-06;Nerve_Tibial; rs34296414;6·124e-08;1·830842e-06;Nerve_Tibial; rs34310656;4·387e-08;1·363515e-06;Nerve_Tibial; rs34583567;4·468e-09;2·277382e-06;Artery_Tibial; rs34796890;3·328e-08;1·216926e-06;Lung; rs35129260;4·205e-08;2·5144e-07;Esophagus_Muscularis; rs35322464;5·281e-08;1·629721e-08;Whole_Blood; rs35395156;1·57e-09;2·277195e-06;Artery_Tibial; rs35722009;5·052e-08;1·047761e-06;Lung; rs35768117;1·095e-10;7·318132e-06;Thyroid; rs35997299;5·112e-08;2·018721e-07;Esophagus_Muscularis; rs36004835;2·657e-09;2·278509e-06;Artery_Tibial; rs3743342;1·133e-07;1·227785e-06;Artery_Aorta; rs3784692;2·515e-09;1·17316e-06;Artery_Tibial; rs3784695;1·168e-17;1·47858e-05;Skin_Sun_Exposed_Lower_leg; rs3784707;2·572e-30;1·384412e-11;Thyroid; rs3784713;2·686e-11;1·570916e-05;Skin_Sun_Exposed_Lower_leg; rs3784719;3·799e-16;1·114565e-06;Adipose_Subcutaneous; rs3825977;2·218e-06;1·373833e-07;Whole_Blood; rs3865018;1·197e-18;1·210362e-07;Breast_Mammary_Tissue; rs405390;4·49e-07;3·209227e-06;Heart_Atrial_Appendage; rs4238411;5·888e-16;3·751164e-06;Breast_Mammary_Tissue; rs4306451;2·852e-10;1·515881e-06;Heart_Atrial_Appendage; rs431489;4·463e-07;2·029818e-06;Heart_Atrial_Appendage; rs4325504;8·416e-17;1·69768e-06;Adipose_Subcutaneous; rs4331293;8·293e-23;1·226489e-10;Adipose_Subcutaneous; rs4363822;4·65e-09;2·276069e-06;Artery_Tibial; rs440341;9·955e-11;1·636554e-05;Thyroid; rs448720;1·048e-10;1·652669e-05;Thyroid; rs4491456;4·95e-08;1·000858e-06;Lung; rs4492996;2·302e-12;1·135805e-06;Heart_Atrial_Appendage; rs4536412;4·158e-08;1·000858e-06;Lung; rs4539549;5·802e-21;1·132909e-05;Skin_Sun_Exposed_Lower_leg; rs47713;4·451e-07;3·169837e-06;Heart_Atrial_Appendage; rs4776350;5·924e-08;1·317473e-06;Nerve_Tibial; rs4776376;8·134e-06;1·114565e-06;Adipose_Subcutaneous; rs4776377;7·816e-06;1·114275e-06;Adipose_Subcutaneous; rs4776382;1·797e-08;1·328231e-05;Thyroid; rs4776383;5·587e-09;1·31988e-05;Thyroid; rs4776913;4·178e-08;1·058618e-06;Lung; rs4776917;3·545e-08;1·121861e-06;Nerve_Tibial; rs4776922;4·235e-08;1·026585e-06;Lung; rs4776942;1·41e-18;1·461929e-11;Nerve_Tibial; rs4776972;5·912e-29;2·134111e-11;Artery_Tibial; rs4776974;8·049e-06;1·114565e-06;Adipose_Subcutaneous; rs4776975;8·317e-06;1·114565e-06;Adipose_Subcutaneous; rs4776976;1·48e-12;1·021769e-11;Adipose_Subcutaneous; rs4776977;8·748e-

					13;2·119319e-09;Whole_Blood; rs4776978;6·964e-13;1·277587e-05;Nerve_Tibial; rs4776982;1·493e-12;1·317525e-05;Nerve_Tibial; rs4776984;9·076e-13;1·237715e-10;Adipose_Subcutaneous; rs4776988;6·863e-12;7·103765e-06;Skin_Sun_Exposed_Lower_leg; rs4776990;2·678e-23;1·007217e-07;Whole_Blood; rs4777009;1·101e-10;1·569281e-05;Thyroid; rs55908993;5·492e-10;2·235041e-07;Heart_Atrial_Appendage; rs55986190;2·991e-14;1·253912e-06;Muscle_Skeletal; rs57030473;3·575e-12;2·308202e-06;Adipose_Subcutaneous; rs57758699;3·271e-15;8·493035e-06;Heart_Atrial_Appendage; rs6494672;2·309e-12;1·738202e-08;Muscle_Skeletal; rs6494678;3·505e-22;1·098057e-08;Thyroid; rs6494696;7·56e-06;1·114275e-06;Adipose_Subcutaneous; rs66926990;1·846e-08;1·027438e-06;Skin_Sun_Exposed_Lower_leg; rs67872952;2·293e-08;1·14172e-07;Esophagus_Muscularis; rs71400364;4·067e-08;1·230235e-06;Nerve_Tibial; rs7162089;5·493e-06;1·256275e-05;Thyroid; rs7166790;2·42e-11;1·027514e-07;Heart_Atrial_Appendage; rs7168156;7·269e-06;1·114275e-06;Adipose_Subcutaneous; rs7169086;1·107e-20;1·076846e-10;Adipose_Subcutaneous; rs7172137;4·504e-06;9·932313e-06;Thyroid; rs7182798;6·138e-08;1·349951e-06;Nerve_Tibial; rs7183168;7·819e-12;7·329433e-06;Skin_Sun_Exposed_Lower_leg; rs72625778;3·776e-08;1·066233e-06;Lung; rs72745470;4·091e-11;1·251281e-05;Adipose_Subcutaneous; rs72745473;1·307e-08;1·635571e-07;Heart_Atrial_Appendage; rs72747464;1·201e-13;1·214548e-06;Thyroid; rs76164701;8·647e-06;1·114641e-06;Adipose_Subcutaneous; rs8025526;1·413e-07;1·775092e-06;Heart_Atrial_Appendage; rs8025774;2·15e-07;1·066797e-05;Nerve_Tibial; rs8025790;8·171e-13;1·174328e-11;Adipose_Subcutaneous; rs8025791;4·39e-06;1·292415e-05;Thyroid; rs8028313;3·842e-13;1·545881e-06;Nerve_Tibial; rs8030456;1·505e-12;1·683911e-08;Whole_Blood; rs8030996;5·595e-15;2·104701e-06;Heart_Atrial_Appendage; rs8031500;5·558e-08;1·539481e-09;Adipose_Subcutaneous; rs8035326;2·391e-10;1·421302e-05;Thyroid; rs8035675;1·974e-09;2·282588e-06;Artery_Tibial; rs8037262;3·648e-16;1·093491e-06;Adipose_Subcutaneous; rs8037476;8·438e-22;1·300606e-13;Artery_Tibial; rs8039943;3·917e-08;1·007274e-06;Lung; rs8042007;4·437e-08;1·000585e-06;Lung; rs8042567;3·586e-17;2·118817e-08;Muscle_Skeletal; rs868036;1·108e-48;1·698393e-06;Adipose_Subcutaneous; rs893475;9·496e-07;8·915664e-06;Artery_Tibial; rs9302245;5·513e-18;2·705639e-08;Heart_Atrial_Appendage; rs9302246;3·098e-18;2·705924e-08;Heart_Atrial_Appendage; rs938875;6·798e-10;1·205208e-06;Muscle_Skeletal; rs9806377;3·633e-08;1·363515e-06;Nerve_Tibial; rs9920643;7·09e-13;2·196126e-09;Whole_Blood; rs9920662;7·252e-06;1·114275e-06;Adipose_Subcutaneous; rs997295;5·866e-09;2·270828e-06;Artery_Tibial;	
SKOR1	7·0E-02	0·1349	rs868036	rs868036	rs10152595;3·623e-06;8·924115e-07;Thyroid; rs10152913;4·134e-08;3·638542e-08;Adipose_Subcutaneous; rs1026731;9·292e-06;1·946017e-08;Thyroid; rs1026733;8·476e-06;1·662754e-08;Thyroid; rs1026734;7·586e-06;1·946017e-08;Thyroid; rs1026735;3·572e-16;1·946017e-08;Thyroid; rs1026737;5·375e-13;1·00519e-06;Thyroid; rs10400882;4·642e-10;6·62852e-06;Adipose_Subcutaneous; rs10438355;9·5e-08;4·464598e-07;Adipose_Subcutaneous; rs10468041;3·596e-12;1·69362e-07;Heart_Left_Ventricle; rs10518739;5·442e-18;1·724165e-06;Heart_Left_Ventricle; rs10775194;5·989e-20;1·504461e-06;Nerve_Tibial; rs10775195;2·629e-07;4·448825e-06;Adipose_Subcutaneous; rs11071940;3·665e-08;2·597901e-06;Thyroid; rs113203559;3·202e-08;4·023326e-08;Adipose_Subcutaneous; rs11401837;2·296e-16;1·228785e-07;Thyroid; rs11451847;3·762e-18;1·155667e-05;Cells_Transformed_fibroblasts; rs11631200;1·025e-14;1·053568e-06;Adipose_Subcutaneous; rs11637027;8·743e-10;1·016298e-06;Adipose_Subcutaneous; rs11638507;1·21e-12;5·236564e-06;Heart_Left_Ventricle; rs11854197;1·121e-16;1·296338e-05;Skin_Sun_Exposed_Lower_leg; rs11856740;1·307e-12;1·87806e-06;Heart_Left_Ventricle; rs11857017;1·499e-22;1·602689e-08;Adipose_Subcutaneous; rs12438118;3·039e-20;1·211606e-05;Nerve_Tibial; rs12439895;2·321e-17;1·207011e-06;Thyroid; rs12441823;7·864e-20;1·609145e-06;Nerve_Tibial; rs12442323;4·109e-08;1·290942e-08;Adipose_Subcutaneous; rs12591495;4·968e-08;3·33134e-06;Thyroid; rs12592101;9·351e-15;1·534501e-05;Skin_Sun_Exposed_Lower_leg; rs12592315;4·026e-15;1·702793e-06;Colon_Transverse; rs12593813;7·892e-17;1·054276e-07;Adipose_Subcutaneous; rs12594229;4·241e-08;3·67307e-08;Adipose_Subcutaneous; rs12594234;4·835e-08;3·671964e-08;Adipose_Subcutaneous; rs12595334;1·279e-07;4·933702e-06;Thyroid; rs12899051;4·385e-08;1·121651e-07;Adipose_Subcutaneous; rs12900758;5·904e-08;4·492032e-06;Thyroid; rs12901789;2·897e-08;1·342207e-05;Thyroid; rs12902804;1·979e-17;1·37902e-05;Skin_Sun_Exposed_Lower_leg; rs12905509;7·015e-17;1·283425e-05;Skin_Sun_Exposed_Lower_leg; rs12910062;6·387e-08;1·39356e-07;Adipose_Subcutaneous; rs12910196;5·201e-08;3·372861e-06;Thyroid; rs12911370;4·42e-08;1·381164e-07;Adipose_Subcutaneous; rs12912974;6·81e-17;1·205251e-05;Skin_Sun_Exposed_Lower_leg; rs12915085;5·893e-08;1·592384e-06;Thyroid; rs12915438;3·904e-16;1·417232e-06;Thyroid; rs12916403;3·837e-08;3·843925e-08;Adipose_Subcutaneous; rs13329413;6·92e-29;1·109415e-05;Artery_Tibial; rs13329567;9·766e-13;1·00761e-06;Thyroid; rs1444935;3·999e-08;3·746073e-08;Adipose_Subcutaneous; rs1478941;3·927e-12;3·078215e-06;Adipose_Subcutaneous; rs16950804;5·276e-08;1·147297e-07;Adipose_Subcutaneous; rs16951001;2·486e-10;1·26723e-06;Heart_Left_Ventricle; rs16951105;1·043e-20;1·053194e-06;Skin_Sun_Exposed_Lower_leg; rs16953610;6·23e-08;3·353857e-06;Thyroid; rs17241403;1·46e-12;1·395114e-06;Heart_Left_Ventricle; rs17242605;4·764e-17;1·350337e-05;Skin_Sun_Exposed_Lower_leg; rs17243334;5·409e-18;1·724165e-06;Heart_Left_Ventricle; rs17526859;3·606e-08;3·638542e-08;Adipose_Subcutaneous; rs1871307;3·062e-16;1·92264e-08;Thyroid; rs2033783;4·35e-08;1·171588e-07;Adipose_Subcutaneous; rs2127163;1·434e-28;1·713678e-06;Cells_Transformed_fibroblasts; rs2241422;7·641e-22;1·773586e-05;Thyroid; rs2289789;6·382e-	NA

	08;3·377116e-06;Thyroid; rs2414952;1·279e-17;1·02291e-06;Cells_Transformed_fibroblasts; rs28376010;1·593e-31;2·164603e-06;Skin_Sun_Exposed_Lower_leg; rs28376697;2·797e-24;2·250393e-07;Skin_Sun_Exposed_Lower_leg; rs28418298;1·161e-20;1·296603e-08;Adipose_Subcutaneous; rs28427879;1·575e-11;2·196003e-09;Adipose_Subcutaneous; rs28565266;8·838e-21;2·04972e-06;Cells_Transformed_fibroblasts; rs28568344;1·574e-26;1·562206e-08;Adipose_Subcutaneous; rs28645602;5·987e-08;3·376577e-06;Thyroid; rs28670272;8·628e-13;1·016536e-05;Testis; rs28696998;3·919e-08;1·309733e-08;Adipose_Subcutaneous; rs28735117;3·495e-22;1·113736e-08;Adipose_Subcutaneous; rs28742003;1·108e-28;1·947454e-08;Nerve_Tibial; rs2899730;1·548e-09;1·224826e-07;Nerve_Tibial; rs338338;9·879e-11;4·622269e-06;Adipose_Subcutaneous; rs34004236;2·952e-20;1·09237e-06;Nerve_Tibial; rs34221449;4·136e-08;3·638542e-08;Adipose_Subcutaneous; rs34296414;6·124e-08;3·353857e-06;Thyroid; rs34310656;4·387e-08;3·638542e-08;Adipose_Subcutaneous; rs34583567;4·468e-09;5·804917e-07;Adipose_Subcutaneous; rs34796890;3·328e-08;2·913297e-06;Thyroid; rs35129260;4·205e-08;1·330801e-05;Thyroid; rs35322464;5·281e-08;1·011503e-07;Adipose_Subcutaneous; rs35395156;1·57e-09;5·693928e-07;Adipose_Subcutaneous; rs35722009;5·052e-08;3·795415e-08;Adipose_Subcutaneous; rs35997299;5·112e-08;3·353857e-06;Thyroid; rs36004835;2·657e-09;5·697383e-07;Adipose_Subcutaneous; rs3743342;1·133e-07;2·770545e-06;Adipose_Subcutaneous; rs3784692;2·515e-09;4·249788e-07;Adipose_Subcutaneous; rs3784695;1·168e-17;1·119679e-05;Skin_Sun_Exposed_Lower_leg; rs3784707;2·572e-30;1·188438e-10;Adipose_Subcutaneous; rs3784713;2·686e-11;3·606142e-06;Adipose_Subcutaneous; rs3784719;3·799e-16;1·946017e-08;Thyroid; rs3825977;2·218e-06;1·621801e-05;Cells_Transformed_fibroblasts; rs383041;5·665e-09;6·749313e-06;Adipose_Subcutaneous; rs3865018;1·197e-18;1·424028e-06;Skin_Sun_Exposed_Lower_leg; rs405390;4·49e-07;2·054739e-06;Adipose_Subcutaneous; rs4238411;5·888e-16;1·812732e-06;Skin_Sun_Exposed_Lower_leg; rs4306451;2·852e-10;1·093716e-06;Adipose_Subcutaneous; rs431489;4·463e-07;2·896497e-06;Adipose_Subcutaneous; rs4325504;8·416e-17;1·06303e-05;Skin_Sun_Exposed_Lower_leg; rs4331293;8·293e-23;2·155694e-05;Thyroid; rs4363822;4·65e-09;5·953221e-07;Adipose_Subcutaneous; rs4491456;4·95e-08;1·362829e-08;Adipose_Subcutaneous; rs4492996;2·302e-12;1·432555e-09;Adipose_Subcutaneous; rs4536412;4·158e-08;1·322701e-08;Adipose_Subcutaneous; rs4539549;5·802e-21;4·547623e-06;Adipose_Subcutaneous; rs47713;4·451e-07;3·038217e-06;Adipose_Subcutaneous; rs4776350;5·924e-08;3·454683e-06;Thyroid; rs4776376;8·134e-06;1·946017e-08;Thyroid; rs4776377;7·816e-06;1·94635e-08;Thyroid; rs4776382;1·797e-08;2·471341e-06;Adipose_Subcutaneous; rs4776383;5·587e-09;2·447165e-06;Adipose_Subcutaneous; rs4776913;4·178e-08;3·792438e-06;Thyroid; rs4776917;3·545e-08;6·403339e-06;Thyroid; rs4776922;4·235e-08;3·768801e-08;Adipose_Subcutaneous; rs4776942;1·41e-18;1·864246e-10;Adipose_Subcutaneous; rs4776972;5·912e-29;2·313471e-06;Cells_Transformed_fibroblasts; rs4776974;8·049e-06;1·946017e-08;Thyroid; rs4776975;8·317e-06;1·946017e-08;Thyroid; rs4776976;1·48e-12;1·033174e-07;Thyroid; rs4776977;8·748e-13;1·100729e-06;Thyroid; rs4776978;6·964e-13;1·338595e-06;Muscle_Skeletal; rs4776982;1·493e-12;1·115185e-05;Nerve_Tibial; rs4776984;9·076e-13;1·350489e-05;Nerve_Tibial; rs4776988;6·863e-12;3·747574e-06;Adipose_Subcutaneous; rs4776990;2·678e-23;1·073603e-06;Artery_Tibial; rs55908993;5·492e-10;1·802644e-06;Skin_Sun_Exposed_Lower_leg; rs57030473;3·575e-12;5·617865e-06;Adipose_Subcutaneous; rs57758699;3·271e-15;1·149729e-07;Adipose_Subcutaneous; rs60734084;2·744e-15;1·118525e-05;Adipose_Subcutaneous; rs6494672;2·309e-12;3·066864e-07;Adipose_Subcutaneous; rs6494678;3·505e-22;1·890915e-05;Thyroid; rs6494696;7·56e-06;1·94635e-08;Thyroid; rs66926990;1·846e-08;3·645306e-06;Heart_Left_Ventricle; rs67872952;2·293e-08;1·135405e-06;Adipose_Subcutaneous; rs71400364;4·067e-08;1·106283e-07;Adipose_Subcutaneous; rs7166790;2·42e-11;3·879686e-06;Heart_Left_Ventricle; rs7168156;7·269e-06;1·94635e-08;Thyroid; rs7169086;1·107e-20;1·017737e-05;Nerve_Tibial; rs7182798;6·138e-08;3·382659e-06;Thyroid; rs7183168;7·819e-12;6·464478e-06;Adipose_Subcutaneous; rs72625778;3·776e-08;3·858673e-08;Adipose_Subcutaneous; rs72747464;1·201e-13;1·156589e-05;Skin_Not_Sun_Exposed_Suprapubic; rs76164701;8·647e-06;1·946017e-08;Thyroid; rs8025526;1·413e-07;1·762995e-05;Adipose_Subcutaneous; rs8025774;2·15e-07;2·153694e-06;Adipose_Subcutaneous; rs8025790;8·171e-13;1·010691e-09;Adipose_Subcutaneous; rs8028313;3·842e-13;1·091988e-09;Adipose_Subcutaneous; rs8030456;1·505e-12;1·226194e-09;Adipose_Subcutaneous; rs8030996;5·595e-15;1·412802e-05;Skin_Sun_Exposed_Lower_leg; rs8031500;5·558e-08;1·024583e-06;Adipose_Subcutaneous; rs8035675;1·974e-09;5·684428e-07;Adipose_Subcutaneous; rs8037262;3·648e-16;1·945822e-06;Skin_Sun_Exposed_Lower_leg; rs8037476;8·438e-22;1·555468e-05;Artery_Tibial; rs8039943;3·917e-08;1·312767e-08;Adipose_Subcutaneous; rs8042007;4·437e-08;1·612667e-08;Adipose_Subcutaneous; rs8042567;3·586e-17;1·068946e-05;Adipose_Subcutaneous; rs868036;1·108e-48;1·068929e-05;Skin_Sun_Exposed_Lower_leg; rs9302245;5·513e-18;1·662244e-06;Heart_Left_Ventricle; rs9302246;3·098e-18;1·659137e-06;Heart_Left_Ventricle; rs938875;6·798e-10;1·419763e-05;Thyroid; rs9806377;3·633e-08;3·638542e-08;Adipose_Subcutaneous; rs9920643;7·09e-13;1·008157e-06;Thyroid; rs9920662;7·252e-06;1·94635e-08;Thyroid; rs997295;5·866e-09;5·671315e-07;Adipose_Subcutaneous;

PIAS1	7·4E-02	0·1374	rs868036	rs868036	rs1001870;2·159e-07;1·547781e-05;Artery_Tibial; rs10152788;1·123e-06;1·501957e-06;Artery_Tibial; rs10400882;4·642e-10;8·409294e-08;Artery_Aorta; rs10775195;2·629e-07;1·252058e-06;Artery_Aorta; rs11071969;7·245e-06;4·991409e-06;Adipose_Subcutaneous; rs11071970;1·493e-08;2·139086e-07;Esophagus_Muscularis; rs11071971;1·49e-06;2·933347e-06;Artery_Tibial; rs11071974;3·096e-06;1·989318e-05;Artery_Aorta; rs111785156;6·883e-08;1·292312e-05;Artery_Tibial; rs12148770;1·416e-08;8·665512e-06;Artery_Tibial; rs12441572;5·255e-11;1·025816e-05;Adipose_Subcutaneous; rs1606524;6·743e-06;5·084498e-06;Adipose_Subcutaneous; rs16951227;9·798e-11;1·417487e-05;Adipose_Subcutaneous; rs17247210;5·795e-09;1·547903e-06;Artery_Tibial; rs17308514;4·185e-06;6·623765e-06;Artery_Aorta; rs2127164;6·44e-11;1·025805e-05;Adipose_Subcutaneous; rs2127165;2·322e-11;1·96575e-05;Adipose_Subcutaneous; rs2127167;5·18e-11;1·026352e-05;Adipose_Subcutaneous; rs2458504;8·294e-06;1·954595e-05;Adipose_Subcutaneous; rs338338;9·879e-11;1·807518e-05;Artery_Tibial; rs338354;3·746e-11;1·315257e-05;Artery_Tibial; rs338371;5·691e-06;2·627577e-08;Nerve_Tibial; rs35768117;1·095e-10;2·132297e-05;Nerve_Tibial; rs379281;5·229e-11;1·5336e-05;Adipose_Subcutaneous; rs383041;5·665e-09;1·337892e-06;Artery_Aorta; rs405390;4·49e-07;6·524515e-07;Artery_Aorta; rs405934;1·404e-07;4·727792e-06;Nerve_Tibial; rs413451;2·755e-08;3·407085e-06;Nerve_Tibial; rs414233;8·689e-11;1·32578e-05;Adipose_Subcutaneous; rs4306451;2·852e-10;5·260301e-07;Artery_Aorta; rs431489;4·463e-07;5·897798e-07;Artery_Aorta; rs440341;9·955e-11;3·633713e-06;Artery_Aorta; rs448720;1·048e-10;1·409745e-05;Artery_Tibial; rs450466;4·622e-08;4·545988e-06;Adipose_Subcutaneous; rs47713;4·451e-07;5·664639e-07;Artery_Aorta; rs4776382;1·797e-08;1·152115e-05;Artery_Aorta; rs4776383;5·587e-09;1·172647e-05;Artery_Aorta; rs4777009;1·101e-10;1·409241e-05;Artery_Tibial; rs56735059;5·262e-11;2·019178e-05;Adipose_Subcutaneous; rs57041894;1·948e-11;1·059564e-05;Adipose_Subcutaneous; rs58317218;3·102e-12;2·269787e-06;Adipose_Subcutaneous; rs59069542;6·635e-11;2·187171e-05;Adipose_Subcutaneous; rs62015113;8·779e-06;1·586239e-06;Skin_Sun_Exposed_Lower_leg; rs62015179;4·901e-11;2·187171e-05;Adipose_Subcutaneous; rs62015181;6·139e-11;9·698815e-06;Adipose_Subcutaneous; rs62015184;5·725e-11;7·577263e-06;Adipose_Subcutaneous; rs62015215;1·311e-10;2·115783e-05;Adipose_Subcutaneous; rs62015217;8·383e-11;7·636153e-06;Adipose_Subcutaneous; rs62015244;5·83e-11;1·536703e-05;Adipose_Subcutaneous; rs62015248;3·897e-11;5·381299e-06;Adipose_Subcutaneous; rs62015249;4·649e-11;5·16471e-06;Adipose_Subcutaneous; rs62015250;4·884e-11;4·739212e-06;Adipose_Subcutaneous; rs62015251;4·399e-11;7·158491e-06;Adipose_Subcutaneous; rs62015253;3·483e-11;1·973664e-06;Adipose_Subcutaneous; rs62016660;4·321e-08;2·800751e-06;Esophagus_Muscularis; rs694267;1·376e-08;1·42909e-05;Adipose_Subcutaneous; rs7162089;5·493e-06;2·526387e-05;Nerve_Tibial; rs7163687;9·639e-06;4·45594e-06;Adipose_Subcutaneous; rs7172137;4·504e-06;1·424455e-05;Adipose_Subcutaneous; rs75654503;1·952e-10;1·683625e-05;Adipose_Subcutaneous; rs8025791;4·39e-06;5·335437e-06;Artery_Aorta; rs8035326;2·391e-10;1·418482e-05;Artery_Tibial; rs922493;7·651e-11;1·10913e-05;Adipose_Subcutaneous; rs9796591;5·593e-08;4·618025e-06;Adipose_Subcutaneous;	NA
TBC1D22B	7·7E-02	0·1387	rs17636328	NA	NA	Enh
CRBN	1·2E-01	0·1821	rs1848460	NA	NA	Enh
SUMF1	1·2E-01	0·1821	rs1848460	NA	NA	Enh
ATP2C1	1·1E-01	0·1821	rs35987657	NA	NA	Enh
COL6A6	1·1E-01	0·1821	rs35987657	NA	NA	Enh
NEK11	1·5E-01	0·2209	rs35987657	NA	NA	Enh
PIK3R4	1·5E-01	0·2209	rs35987657	NA	NA	Enh
TRNT1	2·0E-01	0·2887	rs1848460	NA	NA	Enh
BORA	2·3E-01	0·3184	rs340561	NA	NA	Enh
CMTR1	4·9E-01	0·6742	rs17636328	NA	NA	Enh
DCDC2C	5·3E-01	0·7055	rs10208712	NA	NA	Enh
SETBP1	5·6E-01	0·7275	rs12962305	NA	NA	Enh

C7orf62	6·1E-01	0·7482	rs10952927	rs10952927	NA	NA
C15orf41	6·5E-01	0·7482	rs996064	NA	NA	Enh
DPH6	6·5E-01	0·7482	rs996064	NA	NA	Enh
ETAA1	6·1E-01	0·7482	rs1820989	NA	rs12615683;1·755e-06;6·651875e-08;Adrenal_Gland; rs12713598;2·701e-07;1·490118e-06;Brain_Frontal_Cortex_BA9; rs1568467;5·078e-06;3·584941e-08;Adrenal_Gland; rs2861632;9·143e-07;1·60066e-06;Adrenal_Gland; rs34662163;4·937e-07;1·331127e-06;Cells_Transformed_fibroblasts; rs7594770;8·44e-06;2·61322e-07;Adrenal_Gland;	NA
CCDC148	6·6E-01	0·7482	rs80319144	rs80319144	NA	NA
CCDC167	6·5E-01	0·7482	rs17636328	NA	NA	Enh
ZNF804B	7·1E-01	0·7785	rs10952927	rs10952927	NA	NA
MZT1	7·3E-01	0·7785	rs340561	NA	NA	Enh
IQCH	7·3E-01	0·7785	rs868036	rs868036	rs10152595;3·623e-06;1·161163e-08;Thyroid; rs10152913;4·134e-08;1·233143e-05;Nerve_Tibial; rs10438355;9·5e-08;1·139896e-06;Colon_Transverse; rs11071940;3·665e-08;1·002428e-10;Thyroid; rs113203559;3·202e-08;1·233143e-05;Nerve_Tibial; rs12439895;2·321e-17;1·420215e-05;Esophagus_Mucosa; rs12442323;4·109e-08;1·232016e-05;Nerve_Tibial; rs12591495;4·968e-08;1·109684e-06;Colon_Transverse; rs12594229;4·241e-08;1·245456e-05;Nerve_Tibial; rs12594234;4·835e-08;1·245456e-05;Nerve_Tibial; rs12595334;1·279e-07;1·704751e-06;Colon_Transverse; rs12899051;4·385e-08;1·548296e-05;Esophagus_Mucosa; rs12900758;5·904e-08;1·195978e-06;Colon_Transverse; rs12901789;2·897e-08;1·082061e-10;Thyroid; rs12910062;6·387e-08;1·687191e-05;Artery_Tibial; rs12910196;5·201e-08;1·090851e-06;Colon_Transverse; rs12911370;4·42e-08;1·556997e-06;Skin_Sun_Exposed_Lower_leg; rs12915085;5·893e-08;1·247756e-05;Cells_Transformed_fibroblasts; rs12916403;3·837e-08;1·322595e-05;Nerve_Tibial; rs1444935;3·999e-08;1·284602e-05;Nerve_Tibial; rs16950804;5·276e-08;1·338337e-05;Esophagus_Mucosa; rs16953610;6·23e-08;1·076948e-06;Colon_Transverse; rs17526859;3·606e-08;1·233143e-05;Nerve_Tibial; rs2033783;4·35e-08;1·17977e-06;Colon_Transverse; rs2166185;3·096e-08;1·552355e-08;Cells_Transformed_fibroblasts; rs2289789;6·382e-08;1·102247e-06;Colon_Transverse; rs28645602;5·987e-08;1·102425e-06;Colon_Transverse; rs28696998;3·919e-08;1·24493e-05;Nerve_Tibial; rs34221449;4·136e-08;1·233143e-05;Nerve_Tibial; rs34296414;6·124e-08;1·076948e-06;Colon_Transverse; rs34310656;4·387e-08;1·233143e-05;Nerve_Tibial; rs34796890;3·328e-08;1·253961e-05;Nerve_Tibial; rs35129260;4·205e-08;1·064671e-10;Thyroid; rs35322464;5·281e-08;1·195978e-06;Colon_Transverse; rs35722009;5·052e-08;1·061991e-05;Nerve_Tibial; rs35997299;5·112e-08;1·076948e-06;Colon_Transverse; rs3743342;1·133e-07;1·265142e-10;Thyroid; rs3825977;2·218e-06;9·360941e-09;Thyroid; rs4491456;4·95e-08;1·232053e-05;Nerve_Tibial; rs4536412;4·158e-08;1·232053e-05;Nerve_Tibial; rs4776350;5·924e-08;1·156194e-06;Colon_Transverse; rs4776913;4·178e-08;1·416898e-05;Nerve_Tibial; rs4776917;3·545e-08;1·170525e-06;Skin_Sun_Exposed_Lower_leg; rs4776922;4·235e-08;1·293837e-05;Nerve_Tibial; rs57030473;3·575e-12;2·105886e-06;Cells_Transformed_fibroblasts; rs66926990;1·846e-08;2·006819e-08;Cells_Transformed_fibroblasts; rs67872952;2·293e-08;1·817686e-10;Thyroid; rs71400364;4·067e-08;1·3046e-05;Esophagus_Mucosa; rs7182798;6·138e-08;1·107882e-06;Colon_Transverse; rs72625778;3·776e-08;1·327943e-05;Nerve_Tibial; rs72745470;4·091e-11;4·3953e-09;Thyroid; rs72745473;1·307e-08;1·218874e-07;Cells_Transformed_fibroblasts; rs8025774;2·15e-07;1·002004e-05;Nerve_Tibial; rs8031500;5·558e-08;1·247756e-05;Cells_Transformed_fibroblasts; rs8039943;3·917e-08;1·246608e-05;Nerve_Tibial; rs8042007;4·437e-08;1·231917e-05;Nerve_Tibial; rs9806377;3·633e-08;1·233143e-05;Nerve_Tibial;	NA

C15orf61	7·8E-01	0·8137	rs868036	rs868036	rs12595293;4·823e-08;1·168207e-05;Muscle_Skeletal; rs139107736;7·667e-08;8·458898e-06;Skin_Not_Sun_Exposed_Suprapubic; rs144087544;7·397e-06;9·67596e-06;Skin_Not_Sun_Exposed_Suprapubic; rs144854139;1·186e-06;1·346094e-05;Muscle_Skeletal; rs16950932;2·279e-08;5·9283e-06;Muscle_Skeletal; rs16950946;6·107e-08;1·059358e-05;Muscle_Skeletal; rs16951002;1·952e-06;3·577108e-06;Skin_Not_Sun_Exposed_Suprapubic; rs16951006;8·838e-07;1·358568e-06;Skin_Not_Sun_Exposed_Suprapubic; rs16951007;8·953e-07;1·358568e-06;Skin_Not_Sun_Exposed_Suprapubic; rs16951010;8·918e-07;1·358568e-06;Skin_Not_Sun_Exposed_Suprapubic; rs16951058;7·593e-07;1·361715e-06;Skin_Not_Sun_Exposed_Suprapubic; rs16951060;7·465e-07;1·360624e-06;Skin_Not_Sun_Exposed_Suprapubic; rs2414953;2·344e-08;7·705545e-06;Muscle_Skeletal; rs2583585;1·418e-06;1·049344e-06;Skin_Not_Sun_Exposed_Suprapubic; rs2589977;6·438e-07;1·694323e-06;Muscle_Skeletal; rs2678675;8·31e-07;4·764108e-06;Skin_Not_Sun_Exposed_Suprapubic; rs56264164;1·794e-06;1·26775e-05;Muscle_Skeletal; rs56700149;1·02e-06;1·359994e-06;Skin_Not_Sun_Exposed_Suprapubic; rs56950303;8·536e-07;1·429051e-06;Skin_Not_Sun_Exposed_Suprapubic; rs56951899;5·086e-07;1·085089e-06;Muscle_Skeletal; rs56979146;4·668e-08;4·305738e-06;Muscle_Skeletal; rs59158437;5·353e-07;2·225625e-06;Muscle_Skeletal; rs60359108;3·378e-07;2·435084e-06;Muscle_Skeletal; rs62015061;2·868e-07;1·011233e-05;Muscle_Skeletal; rs62015063;2·905e-07;8·223303e-06;Skin_Not_Sun_Exposed_Suprapubic; rs62015113;8·779e-06;1·011418e-05;Muscle_Skeletal; rs62016025;5·991e-07;4·779978e-06;Muscle_Skeletal; rs62016030;3·808e-07;4·994014e-06;Muscle_Skeletal; rs62016031;2·986e-08;9·693002e-06;Muscle_Skeletal; rs62016057;3·261e-08;3·689023e-06;Muscle_Skeletal; rs62016162;2·08e-07;8·96476e-07;Skin_Not_Sun_Exposed_Suprapubic; rs7164467;3·99e-08;5·136229e-06;Muscle_Skeletal; rs7164713;1·085e-06;1·258777e-05;Muscle_Skeletal; rs7167280;7·936e-07;8·732971e-06;Muscle_Skeletal; rs7170143;8·603e-07;1·288204e-06;Skin_Not_Sun_Exposed_Suprapubic; rs7178729;2·731e-08;5·798928e-06;Muscle_Skeletal; rs7182417;5·854e-07;2·985195e-06;Muscle_Skeletal; rs74023019;8·342e-07;1·347046e-06;Skin_Not_Sun_Exposed_Suprapubic; rs79790429;8·73e-07;4·147626e-06;Muscle_Skeletal; rs8028912;8·227e-07;4·076753e-06;Skin_Not_Sun_Exposed_Suprapubic; rs8030906;3·95e-07;8·223303e-06;Skin_Not_Sun_Exposed_Suprapubic; rs8035065;3·784e-08;9·366861e-06;Muscle_Skeletal;
AAGAB	8·2E-01	0·8351	rs868036	rs868036	rs4776926;8·185e-07;1·703382e-05;Esophagus_Muscularis;
CALML4	9·4E-01	0·9375	rs868036	rs868036	rs17247210;5·795e-09;2·455987e-05;Nerve_Tibial;

Supplementary Table 9: Results of gene set (pathway) enrichment analysis using DEPICT. Original gene set ID, identifier of the predefined gene set; Original gene set description, description of of the predefined gene set; Nominal p value, nominal enrichment p value of the reconstituted gene set (Null hypothesis: Genes in associated loci do not enrich for the reconstituted gene set); False discovery rate, estimated false discovery rate for the reconstituted gene set.

Original gene set ID	Original gene set description	Nominal p value	False discovery rate
ENSG00000090372	STRN4 PPI subnetwork	1·33E-04	>=0·20
GO:0060479	lung cell differentiation	1·71E-04	>=0·20
GO:0015074	DNA integration	3·28E-04	>=0·20
MP:0001939	secondary sex reversal	3·61E-04	>=0·20
GO:0000079	regulation of cyclin-dependent protein kinase activity	6·23E-04	>=0·20
GO:0060487	lung epithelial cell differentiation	7·26E-04	>=0·20
ENSG00000110876	SELPLG PPI subnetwork	8·19E-04	>=0·20
REACTOME_MYOGENESIS	REACTOME_MYOGENESIS	9·34E-04	>=0·20
REACTOME_CDO_IN_MYOGENESIS	REACTOME_CDO_IN_MYOGENESIS	9·34E-04	>=0·20
GO:0042430	indole-containing compound metabolic process	1·10E-03	>=0·20
GO:0006586	indolalkylamine metabolic process	1·10E-03	>=0·20
REACTOME_G1_PHASE	REACTOME_G1_PHASE	1·15E-03	>=0·20
REACTOME_CYCLIN_D_ ASSOCIATED_EVENTS_IN_G1	REACTOME_CYCLIN_D_ ASSOCIATED_EVENTS_IN_G1	1·15E-03	>=0·20
GO:0002089	lens morphogenesis in camera-type eye	1·21E-03	>=0·20
GO:0007215	glutamate receptor signaling pathway	1·33E-03	>=0·20
GO:0042166	acetylcholine binding	1·55E-03	>=0·20
ENSG00000123066	MED13L PPI subnetwork	1·60E-03	>=0·20
ENSG00000175220	ARHGAP1 PPI subnetwork	1·74E-03	>=0·20
ENSG00000103351	CLUAP1 PPI subnetwork	1·80E-03	>=0·20
GO:0004712	protein serine/threonine/tyrosine kinase activity	1·82E-03	>=0·20
GO:0035265	organ growth	2·02E-03	>=0·20
GO:0051568	histone H3-K4 methylation	2·15E-03	>=0·20
GO:0043372	positive regulation of CD4-positive, alpha-beta T cell differentiation	2·19E-03	>=0·20
GO:2000516	positive regulation of CD4-positive, alpha-beta T cell activation	2·19E-03	>=0·20

ENSG00000124762	CDKN1A PPI subnetwork	2·28E-03	>=0·20
GO:0045624	positive regulation of T-helper cell differentiation	2·36E-03	>=0·20
ENSG00000163464	CXCR1 PPI subnetwork	2·54E-03	>=0·20
ENSG00000106105	GARS PPI subnetwork	2·56E-03	>=0·20
MP:0008478	increased spleen white pulp amount	2·73E-03	>=0·20
ENSG00000169057	MECP2 PPI subnetwork	2·85E-03	>=0·20
ENSG00000103343	ZNF174 PPI subnetwork	3·25E-03	>=0·20
ENSG00000147677	EIF3H PPI subnetwork	4·02E-03	>=0·20
ENSG00000124782	RREB1 PPI subnetwork	4·23E-03	>=0·20
GO:0032355	response to estradiol stimulus	4·33E-03	>=0·20
ENSG00000180871	CXCR2 PPI subnetwork	4·77E-03	>=0·20
GO:0001508	regulation of action potential	4·85E-03	>=0·20
ENSG00000105173	CCNE1 PPI subnetwork	5·00E-03	>=0·20
MP:0000611	jaundice	5·01E-03	>=0·20
ENSG00000188687	SLC4A5 PPI subnetwork	5·19E-03	>=0·20
ENSG00000134574	DDB2 PPI subnetwork	5·25E-03	>=0·20
MP:0002572	abnormal emotion/affect behavior	5·45E-03	>=0·20
ENSG00000163879	DNALI1 PPI subnetwork	5·46E-03	>=0·20
GO:0045620	negative regulation of lymphocyte differentiation	5·54E-03	>=0·20
GO:0009070	serine family amino acid biosynthetic process	5·69E-03	>=0·20
ENSG00000063322	MED29 PPI subnetwork	5·90E-03	>=0·20
ENSG00000182944	EWSR1 PPI subnetwork	6·15E-03	>=0·20
GO:0009264	deoxyribonucleotide catabolic process	6·22E-03	>=0·20
GO:0032673	regulation of interleukin-4 production	6·27E-03	>=0·20
GO:0002828	regulation of type 2 immune response	6·62E-03	>=0·20

Supplementary Table 10: Results of gene set (pathway) analysis using DEPICT and UniProtKB annotations. Significance level ("p") and false discovery rate ("FDR") of a one-sided Fisher test for enrichment. "Enrichment" is calculated as the observed count ("Count") divided by the count ("ExpCount") that is expected under the assumption of no enrichment. "Size" is the total number of genes that are annotated in the functional item.

UniProtKB biological process term	p	Enrichment	ExpCount	Count	Size	FDR
Neurogenesis	0·0017	16·7979	0·2516	3	244	0·0487
Differentiation	0·0245	6·0583	0·6631	3	643	0·0935
Transcription	0·0684	3·1468	2·4141	5	2341	0·1910
DNA damage	0·2943	3·1407	0·3424	1	332	0·1910
Host-virus interaction	0·3289	2·7363	0·3908	1	379	0·1910
Cell adhesion	0·3890	2·2036	0·4806	1	466	0·1910
Apoptosis	0·4270	1·9430	0·5414	1	525	0·1914

Supplementary Table 11: Results of tissue enrichment analysis using BI-ENRICH. p, permutation-based empirical significance level; FDR, empirical false discovery rate (adjusted q-value based on Benjamini-Hochberg method); z, signed weighted z-score; sampling-p-value, empirical p value determined by random resampling of pseudo candidate gene sets; sampling-FDR, empirical false discovery rate determined by random resampling of pseudo candidate gene sets; Scaled.FC for candidate genes, candidate genes sorted by scaled fold change.

tissue type	p	FDR	z	sampling-p-value	sampling-FDR	Scaled.FC for candidate genes
Brain - Spinal cord (cervical c-1)	0·024	0·553	3·771	0	0	PKP4(5); PTPRD(3·289); PTPRD(3·289); TOX3(2·11); HOXB2(1·745); HOXB7(1·576); HOXB8(1·563); MYT1(1·44); CCDC167(1·372); CCDC148(1·341); HOXB5(1·221); ATP2C1(1·1); HOXB6(0·965); NEK11(0·928); MDGA1(0·827); HOXB3(0·788); HOXB9(0·642); GLO1(0·562); NTNG1(0·56); SEMA6D(0·535); PRMT6(0·492); CRBN(0·485); SKOR1(0·269); TRNT1(0·252); DPH6(0·218); MZT1(0·213); ASTE1(0·201); MEIS1(0·185); IQCH(0·165); DACH1(0·016)
Brain - Hypothalamus	0·089	0·553	3·359	0·006	0·294	MYT1(3·653); PTPRD(2·26); PTPRD(2·26); NTNG1(1·396); PKP4(1·365); TOX3(1·365); SEMA6D(1·066); NEK11(0·974); MZT1(0·923); CCDC148(0·862); PRMT6(0·732); BTBD9(0·68); CCDC167(0·629); GLO1(0·628); MDGA1(0·627); ATP2C1(0·536); IQCH(0·521); DACH1(0·478); RNF8(0·44); C15orf61(0·402); SKOR1(0·2); AAGAB(0·198); ZNF804B(0·161); TRNT1(0·042); CRBN(0·012); MAP2K5(0·001)
Brain - Frontal Cortex (BA9)	0·115	0·553	3·154	0·001	0·049	MYT1(2·033); PTPRD(1·947); PTPRD(1·947); SEMA6D(1·383); NEK11(1·364); NTNG1(1·332); BTBD9(1·264); MZT1(1·252); PKP4(0·979); TOX3(0·902); GLO1(0·899); C15orf61(0·851); RNF8(0·578); ATP2C1(0·493); CCDC148(0·461); MDGA1(0·397); AAGAB(0·392); PRMT6(0·38); CCDC167(0·332); MAP2K5(0·289); DACH1(0·27); CRBN(0·183); SKOR1(0·174); ZNF804B(0·135); TRNT1(0·107); IQCH(0·052); CLN6(0·046)
Brain - Amygdala	0·129	0·553	3·083	0·09	1	MYT1(3·714); PTPRD(2·123); PTPRD(2·123); PKP4(1·967); TOX3(1·227); SEMA6D(1·089); NTNG1(1·089); MZT1(1·05); NEK11(0·95); BTBD9(0·901); CCDC148(0·778); CCDC167(0·61); MDGA1(0·532); GLO1(0·482); PRMT6(0·468); C15orf61(0·464); ATP2C1(0·375); IQCH(0·273); RNF8(0·117); TRNT1(0·03)
Brain - Cortex	0·104	0·553	3·029	0·069	1	PTPRD(2·151); PTPRD(2·151); MYT1(1·855); BTBD9(1·53); NTNG1(1·301); NEK11(1·284); SEMA6D(1·235); MZT1(0·868); PKP4(0·785); TOX3(0·727); C15orf61(0·598); RNF8(0·523); GLO1(0·482); MDGA1(0·469); CCDC148(0·441); MAP2K5(0·437); CCDC167(0·379); SKOR1(0·347); AAGAB(0·347); ATP2C1(0·257); CLN6(0·164); PRMT6(0·088); ZNF804B(0·07); CMTR1(0·009)
Brain - Anterior cingulate cortex (BA24)	0·132	0·553	3·028	0·007	0·343	MYT1(2·546); PTPRD(1·923); PTPRD(1·923); NEK11(1·432); TOX3(1·327); NTNG1(1·321); BTBD9(1·236); SEMA6D(1·164); PKP4(1·017); MZT1(0·984); GLO1(0·813); CCDC167(0·674); CCDC148(0·661); MDGA1(0·624); C15orf61(0·599); PRMT6(0·421); ATP2C1(0·379); RNF8(0·377); ZNF804B(0·259); AAGAB(0·226); MAP2K5(0·206); SKOR1(0·187); DACH1(0·172); CRBN(0·152); IQCH(0·117); C7orf62(0·023); TRNT1(0·019)
Brain - Hippocampus	0·142	0·553	3·007	0·053	1	PKP4(2·909); PTPRD(2·528); PTPRD(2·528); MYT1(2·468); TOX3(1·206); SEMA6D(1·038); BTBD9(0·897); NTNG1(0·891); MZT1(0·843); CCDC148(0·836); NEK11(0·812); CCDC167(0·673); MDGA1(0·571); ATP2C1(0·493); PRMT6(0·465); C15orf61(0·434); GLO1(0·385); IQCH(0·331); RNF8(0·126); MAP2K5(0·096); TRNT1(0·084); CRBN(0·017)
Pituitary	0·051	0·553	2·933	0·097	1	MYT1(3·077); IQCH(1·785); COL6A6(1·548); AAGAB(1·302); C15orf61(1·166); PRMT6(1·161); CCDC148(1·105); TOX3(1·012); DACH1(0·989); CMTR1(0·968); NEK11(0·888); ATP2C1(0·869); TRNT1(0·709); NTNG1(0·633); PTPRD(0·546); PTPRD(0·546); SKOR1(0·534); PIK3R4(0·532); SEMA6D(0·481); RNF8(0·414); ASTE1(0·339); BTBD9(0·322); MDGA1(0·224); MZT1(0·124); SUMF1(0·092); TBC1D22B(0·027)
Brain - Nucleus accumbens (basal ganglia)	0·177	0·553	2·678	0·018	0·882	MYT1(3·087); MZT1(1·784); PTPRD(1·613); PTPRD(1·613); NEK11(1·404); SEMA6D(1·298); BTBD9(1·238); PKP4(1·189); C15orf61(0·951); DACH1(0·883); CCDC148(0·873); NTNG1(0·862); GLO1(0·559); CCDC167(0·465); C15orf41(0·372); PRMT6(0·338); TOX3(0·329); MDGA1(0·307); ATP2C1(0·291); RNF8(0·262); IQCH(0·259); MAP2K5(0·14); SKOR1(0·133); AAGAB(0·053); TRNT1(0·015)
Artery - Aorta	0·192	0·553	2·652	0·434	1	PTPRD(1·713); PTPRD(1·713); SUMF1(1·554); HOXB2(1·266); MEIS1(1·209); SETBP1(1·156); MDGA1(0·998); ETAA1(0·919); DCDC2C(0·782); RNF8(0·756); SMAD3(0·72); SEMA6D(0·672); HOXB3(0·667); DIS3(0·603); PIK3R4(0·514); PIAS1(0·426); C15orf41(0·415); MZT1(0·335); CMTR1(0·288); DPH6(0·252); TRNT1(0·195); TBC1D22B(0·189); HOXB6(0·157); HOXB7(0·148); HOXB5(0·143); CRBN(0·125); ATP2C1(0·081); SKOR1(0·021)
Brain - Caudate (basal ganglia)	0·21	0·572	2·623	0·011	0·539	MYT1(3·344); PTPRD(2·582); PTPRD(2·582); SEMA6D(1·622); PKP4(1·579); MZT1(1·41); DACH1(1·3); NEK11(1·151); BTBD9(1·081); C15orf61(0·774); CCDC148(0·745); NTNG1(0·699); MDGA1(0·691); GLO1(0·524); TOX3(0·435); C15orf41(0·427); ATP2C1(0·421); CCDC167(0·316); IQCH(0·31); RNF8(0·215); PRMT6(0·194); SKOR1(0·1); TRNT1(0·006); AAGAB(0·001)
Brain - Substantia nigra	0·227	0·585	2·595	0·037	1	PKP4(3·847); MYT1(3·027); PTPRD(2·052); PTPRD(2·052); TOX3(1·337); NEK11(1·313); SEMA6D(1·221); CCDC167(1·194); CCDC148(1·181); NTNG1(1·021); ATP2C1(0·62); GLO1(0·595); PRMT6(0·491); MZT1(0·478); MDGA1(0·475); BTBD9(0·359); CRBN(0·263); RNF8(0·188); C15orf61(0·165); TRNT1(0·15); DPH6(0·075); IQCH(0·031)
Thyroid	0·118	0·553	2·523	0·306	1	ZNF804B(2·277); NEK11(1·726); SMAD3(1·57); IQCH(1·56); PKP4(1·302); TRNT1(1·13); SUMF1(1·051); AAGAB(1·023); ETAA1(0·775); PIK3R4(0·761); CLN6(0·705); CCDC148(0·696); TBC1D22B(0·679); ASTE1(0·67); SEMA6D(0·661); PIAS1(0·626); DPH6(0·595); SETBP1(0·595); CMTR1(0·472); PRMT6(0·399); DIS3(0·394); BORA(0·286); PTPRD(0·143); PTPRD(0·143); C7orf62(0·109); MYT1(0·023); CRBN(0·012); ATP2C1(0·012)
Brain - Putamen (basal ganglia)	0·275	0·613	2·319	0·035	1	MYT1(2·733); PTPRD(2·225); PTPRD(2·225); PKP4(2·17); SEMA6D(1·419); MZT1(1·364); DACH1(1·166); BTBD9(0·925); NEK11(0·888); C15orf61(0·729); CCDC148(0·713); NTNG1(0·606); CCDC167(0·503); MDGA1(0·46); GLO1(0·443); C15orf41(0·442); TOX3(0·301); ATP2C1(0·3); RNF8(0·229); PRMT6(0·216); SKOR1(0·157); AAGAB(0·037); MAP2K5(0·028)

Brain - Cerebellar Hemisphere	0·167	0·553	2·263	0·098	1	MYT1(5·435); MDGA1(4·113); PTPRD(2·794); PTPRD(2·794); MEIS1(1·423); MZT1(1·32); PKP4(1·255); C15orf61(1·131); SKOR1(1·127); RNF8(0·896); NTNG1(0·532); SETBP1(0·512); TBC1D22B(0·42); CCDC148(0·419); C15orf41(0·345); CMTR1(0·314); BORA(0·309); MAP2K5(0·253); CALML4(0·124); CLN6(0·123); ATP2C1(0·119); PRMT6(0·102); BTBD9(0·085); ASTE1(0·082); TRNT1(0·077); CRBN(0·041); DIS3(0·034); ETAA1(0·03)
Kidney - Cortex	0·134	0·553	2·206	0·069	1	HOXB9(6·144); HOXB8(5·322); HOXB7(5·22); HOXB6(4·491); TOX3(3·845); HOXB5(3·408); HOXB2(2·831); HOXB3(2·286); DACH1(2·154); PKP4(1·836); CCDC148(1·625); SUMF1(1·251); IQCH(1·199); CALML4(0·951); PRMT6(0·859); NEK11(0·808); CLN6(0·796); NTNG1(0·728); AAGAB(0·726); PTPRD(0·696); PTPRD(0·696); SETBP1(0·614); CCDC167(0·479); SMAD3(0·472); SEMA6D(0·452); TRNT1(0·422); TBC1D22B(0·359); ETAA1(0·168); ZNF804B(0·161); RNF8(0·117); GLO1(0·087); MEIS1(0·064); ASTE1(0·046)
Pancreas	0·166	0·553	2·116	0·677	1	MEIS1(2·38); TOX3(1·225); PRMT6(1·113); SEMA6D(1·038); ZNF804B(0·83); ETAA1(0·79); CLN6(0·498); CALML4(0·412); IQCH(0·323); C7orf62(0·321); SUMF1(0·308); C15orf61(0·245); DPH6(0·129); HOXB5(0·113); CCDC167(0·1); MYT1(0·094); BORA(0·071); TBC1D22B(0·056); ASTE1(0·02); SETBP1(0·017); MAP2K5(0·004)
Adrenal Gland	0·172	0·553	2·073	0·131	1	MEIS1(3·102); HOXB7(1·988); HOXB8(1·626); PRMT6(1·555); HOXB6(1·528); ETAA1(1·409); HOXB5(1·372); TBC1D22B(1·137); HOXB2(1·082); NEK11(1·047); AAGAB(0·944); SUMF1(0·89); DACH1(0·862); CLN6(0·859); C15orf41(0·765); HOXB3(0·763); RNF8(0·703); CMTR1(0·639); MAP2K5(0·607); MYT1(0·587); GLO1(0·558); TRNT1(0·545); PIK3R4(0·48); DPH6(0·421); ATP2C1(0·418); ASTE1(0·397); IQCH(0·379); CCDC167(0·303); HOXB9(0·303); BTBD9(0·14); CALML4(0·106); PIAS1(0·083); MZT1(0·081); CRBN(0·003)
Brain - Cerebellum	0·189	0·553	2·062	0·044	1	MYT1(3·912); PTPRD(3·394); PTPRD(3·394); MDGA1(2·985); SKOR1(1·557); RNF8(1·169); C15orf61(1·071); MZT1(0·913); PKP4(0·892); MEIS1(0·805); CMTR1(0·724); NTNG1(0·605); MAP2K5(0·529); CCDC148(0·398); SETBP1(0·376); TBC1D22B(0·323); DIS3(0·24); BORA(0·214); BTBD9(0·213); ATP2C1(0·165); CALML4(0·149); C15orf41(0·098); ASTE1(0·067); PRMT6(0·057); TRNT1(0·056); CRBN(0·039); ETAA1(0·03); SEMA6D(0·029); CLN6(0·022)
Artery - Tibial	0·405	0·763	2·000	0·165	1	SETBP1(2·802); CRBN(1·14); DPH6(1·134); DIS3(0·88); PIAS1(0·745); SUMF1(0·716); MZT1(0·672); PIK3R4(0·668); C15orf41(0·656); ETAA1(0·606); APLF(0·545); DCDC2C(0·51); MDGA1(0·51); BTBD9(0·496); MAP2K5(0·328); PTPRD(0·288); PTPRD(0·288); SMAD3(0·287); TRNT1(0·24); SKOR1(0·131); SEMA6D(0·107); HOXB7(0·058)
Esophagus - Mucosa	0·274	0·613	1·562	0·486	1	SUMF1(1·236); RNF8(1·175); TBC1D22B(1·139); C15orf41(1·094); SMAD3(1·091); MEIS1(1·049); HOXB2(0·89); APLF(0·816); AAGAB(0·809); CCDC167(0·8); BORA(0·786); HOXB3(0·759); DIS3(0·569); PIAS1(0·493); PRMT6(0·436); ETAA1(0·389); PKP4(0·321); CLN6(0·292); GLO1(0·175); DPH6(0·125); TRNT1(0·111); BTBD9(0·094); MDGA1(0·08); CALML4(0·067)
Cells - EBV-transformed lymphocytes	0·378	0·763	1·561	0·638	1	BORA(2·703); CCDC167(1·672); DIS3(1·654); CMTR1(1·33); PRMT6(1·209); ETAA1(1·2); PIAS1(1·16); TRNT1(1·155); MZT1(1·072); CLN6(1·057); GLO1(1·008); ATP2C1(0·964); RNF8(0·802); DPH6(0·682); ASTE1(0·649); PIK3R4(0·583); TBC1D22B(0·505); AAGAB(0·443); CALML4(0·422); C15orf41(0·398); HOXB7(0·33); HOXB9(0·233); CRBN(0·193); SMAD3(0·17); HOXB3(0·161); HOXB2(0·101); APLF(0·047); MAP2K5(0·018); NTNG1(0·009)
Heart - Atrial Appendage	0·5	0·818	1·441	0·651	1	COL6A6(1·731); CCDC167(0·976); BTBD9(0·773); C15orf61(0·718); SEMA6D(0·697); SMAD3(0·571); HOXB2(0·564); RNF8(0·52); GLO1(0·493); MEIS1(0·471); C15orf41(0·337); ZNF804B(0·289); DPH6(0·273); DACH1(0·203); CMTR1(0·102); SETBP1(0·01)
Lung	0·421	0·764	1·391	0·476	1	HOXB5(2·673); COL6A6(2·547); HOXB6(2·228); MEIS1(1·682); DACH1(1·316); HOXB3(1·05); PTPRD(1·045); PTPRD(1·045); ATP2C1(1·017); NTNG1(0·956); HOXB2(0·869); PIAS1(0·815); SETBP1(0·767); DIS3(0·719); BTBD9(0·695); SUMF1(0·679); PIK3R4(0·507); ETAA1(0·501); CMTR1(0·427); GLO1(0·415); TRNT1(0·351); HOXB7(0·33); TBC1D22B(0·299); SEMA6D(0·257); SMAD3(0·252); CALML4(0·234); BORA(0·214); AAGAB(0·213); CLN6(0·099); IQCH(0·004); RNF8(0·002)
Artery - Coronary	0·542	0·818	1·267	0·427	1	HOXB2(2·775); PTPRD(1·649); PTPRD(1·649); SETBP1(1·551); SUMF1(1·326); ETAA1(1·152); HOXB3(0·776); PIK3R4(0·664); C15orf41(0·631); PIAS1(0·564); TRNT1(0·564); DIS3(0·544); SMAD3(0·492); MZT1(0·445); COL6A6(0·425); CRBN(0·388); MDGA1(0·369); ASTE1(0·269); DPH6(0·24); DCDC2C(0·2); APLF(0·155); RNF8(0·128); ATP2C1(0·128); SEMA6D(0·046); BORA(0·032); NEK11(0·025); PRMT6(0·011)
Nerve - Tibial	0·573	0·818	1·103	0·441	1	SEMA6D(1·503); HOXB7(1·389); DCDC2C(1·366); PIK3R4(1·326); ASTE1(1·162); DIS3(1·072); CMTR1(0·94); NTNG1(0·804); HOXB2(0·798); ETAA1(0·779); HOXB3(0·699); ATP2C1(0·684); SUMF1(0·632); DPH6(0·624); PIAS1(0·599); MAP2K5(0·592); CRBN(0·57); TRNT1(0·533); CALML4(0·508); NEK11(0·42); SETBP1(0·395); GLO1(0·285); CLN6(0·255); PRMT6(0·253); APLF(0·242); MDGA1(0·175); SMAD3(0·031); SKOR1(0·004)
Cells - Transformed fibroblasts	0·687	0·840	0·998	0·492	1	SUMF1(1·624); ATP2C1(1·609); DIS3(1·594); ETAA1(1·517); PRMT6(1·286); HOXB2(1·282); C15orf41(1·235); APLF(1·1); SMAD3(1·074); BORA(0·953); HOXB7(0·938); GLO1(0·842); AAGAB(0·809); PIK3R4(0·805); TRNT1(0·741); HOXB3(0·738); HOXB6(0·729); C15orf61(0·716); RNF8(0·628); MZT1(0·628); NTNG1(0·587); CLN6(0·562); MAP2K5(0·516); CCDC167(0·483); NEK11(0·469); ASTE1(0·409); HOXB5(0·368); PIAS1(0·344); IQCH(0·333); DPH6(0·191); BTBD9(0·134); CRBN(0·09); CMTR1(0·079)
Esophagus - Gastroesophageal Junction	0·562	0·818	0·930	0·436	1	MEIS1(4·737); HOXB2(4·415); HOXB3(4·084); SETBP1(2·06); ETAA1(1·08); CALML4(1·027); HOXB5(0·958); CMTR1(0·921); C15orf41(0·86); PKP4(0·82); PIK3R4(0·622); HOXB6(0·604); PIAS1(0·601); APLF(0·528); CRBN(0·429); MAP2K5(0·362); TRNT1(0·302); PRMT6(0·265); SEMA6D(0·208); SKOR1(0·186); MDGA1(0·177); DIS3(0·164); ASTE1(0·142); DPH6(0·08); SUMF1(0·06); PTPRD(0·029); PTPRD(0·029); NTNG1(0·025); RNF8(0·022)
Colon - Sigmoid	0·565	0·818	0·879	0·139	1	MEIS1(4·755); HOXB3(4·402); HOXB6(3·81); HOXB2(3·706); HOXB5(3·418); SETBP1(2·078); HOXB7(1·681); CMTR1(1·197); C15orf41(1·121); APLF(1·083); MAP2K5(0·695); HOXB8(0·494); NTNG1(0·477); ETAA1(0·462); PRMT6(0·346); CRBN(0·34); RNF8(0·333); PIK3R4(0·332); SKOR1(0·299); HOXB9(0·276); PIAS1(0·188); MDGA1(0·172); TRNT1(0·146); SEMA6D(0·126); ASTE1(0·105); CCDC167(0·068)
Muscle - Skeletal	0·662	0·840	0·737	0·479	1	C15orf61(1·772); GLO1(1·123); SMAD3(1·022); CRBN(0·745); AAGAB(0·72); SETBP1(0·682); MZT1(0·68); C15orf41(0·606); TBC1D22B(0·574); DPH6(0·55); MAP2K5(0·542); SEMA6D(0·207); C7orf62(0·186); PIAS1(0·155); HOXB7(0·141); CLN6(0·138); APLF(0·137); DACH1(0·13); TRNT1(0·115); DIS3(0·11); HOXB2(0·076); HOXB9(0·074); ZNF804B(0·049)

Esophagus - Muscularis	0·639	0·840	0·680	0·569	1	MEIS1(4·33); HOXB2(3·9); HOXB3(3·62); SETBP1(1·789); PKP4(1·512); C15orf41(1·198); CALML4(1·188); ETAA1(0·942); CMTR1(0·892); PIK3R4(0·68); APLF(0·588); PIAS1(0·532); CRBN(0·52); TRNT1(0·394); HOXB5(0·358); MAP2K5(0·354); PRMT6(0·242); RNF8(0·124); MDGA1(0·051); HOXB6(0·044); DIS3(0·042); NEK11(0·039); AAGAB(0·037); SKOR1(0·025)
Minor Salivary Gland	0·568	0·818	0·596	0·642	1	TOX3(2·247); MEIS1(1·672); SUMF1(1·359); PKP4(1·34); TBC1D22B(1·225); ETAA1(1·117); CCDC148(0·733); CLN6(0·59); BORA(0·587); COL6A6(0·542); APLF(0·534); ATP2C1(0·385); DIS3(0·361); CALML4(0·333); PIK3R4(0·231); PRMT6(0·226); TRNT1(0·202); CCDC167(0·186); AAGAB(0·153); PIAS1(0·13); DPH6(0·093); ASTE1(0·022)
Colon - Transverse	0·582	0·818	0·558	0·635	1	HOXB5(5·183); HOXB6(4·814); HOXB3(4·207); HOXB7(3·349); HOXB8(1·838); HOXB9(1·758); CALML4(1·54); SEMA6D(1·481); TOX3(1·401); ASTE1(1·007); BORA(1·004); PRMT6(0·903); HOXB2(0·867); SUMF1(0·805); AAGAB(0·786); MEIS1(0·752); CLN6(0·541); TRNT1(0·507); PTPRD(0·468); PTPRD(0·468); MAP2K5(0·38); PIK3R4(0·26); DIS3(0·171); CRBN(0·159); SETBP1(0·127); ETAA1(0·103); CMTR1(0·054); DACH1(0·05); BTBD9(0·045)
Heart - Left Ventricle	0·736	0·843	0·348	0·647	1	C15orf41(3·093); CCDC167(1·212); C15orf61(1·052); COL6A6(1·034); MEIS1(0·795); BTBD9(0·691); DPH6(0·55); DACH1(0·511); GLO1(0·455); SEMA6D(0·453); PTPRD(0·328); PTPRD(0·328); MAP2K5(0·314); ATP2C1(0·19); AAGAB(0·146); HOXB2(0·124); RNF8(0·115); CRBN(0·11); C7orf62(0·058); DIS3(0·038)
Adipose - Subcutaneous	0·825	0·898	0·239	0·556	1	HOXB7(2·621); SMAD3(1·563); HOXB3(1·445); PIK3R4(1·293); HOXB2(1·258); CRBN(1·222); SUMF1(1·151); SETBP1(1·142); DIS3(1·124); DACH1(1·093); HOXB6(0·99); C15orf61(0·779); APLF(0·76); DPH6(0·742); MAP2K5(0·682); PKP4(0·559); HOXB8(0·394); ETAA1(0·327); ASTE1(0·281); PIAS1(0·279); CMTR1(0·25); TRNT1(0·239); HOXB5(0·235); CALML4(0·184); ATP2C1(0·16); HOXB9(0·148); COL6A6(0·147); CLN6(0·138); SKOR1(0·124); BORA(0·078); SEMA6D(0·041)
Small Intestine - Terminal Ileum	0·661	0·840	0·144	0·596	1	HOXB5(4·408); HOXB7(4·364); HOXB6(3·982); HOXB3(3·905); HOXB8(3·278); HOXB9(2·581); CALML4(1·922); TOX3(1·708); SEMA6D(1·58); ASTE1(1·558); DACH1(1·226); BORA(1·189); SMAD3(1·103); PTPRD(0·899); PTPRD(0·899); AAGAB(0·76); HOXB2(0·683); PIAS1(0·672); CLN6(0·653); PRMT6(0·602); ETAA1(0·587); BTBD9(0·449); TBC1D22B(0·44); CMTR1(0·413); PIK3R4(0·397); DIS3(0·325); SUMF1(0·257); GLO1(0·246); MEIS1(0·205); CRBN(0·132); TRNT1(0·115); SKOR1(0·001)
Spleen	0·584	0·818	0·036	0·731	1	BORA(1·307); HOXB3(1·206); ASTE1(1·081); CLN6(0·986); CMTR1(0·92); CALML4(0·9); HOXB5(0·835); PRMT6(0·759); HOXB2(0·758); DACH1(0·711); TRNT1(0·602); CCDC167(0·602); AAGAB(0·594); MEIS1(0·522); PIK3R4(0·438); HOXB7(0·416); HOXB6(0·376); ETAA1(0·35); SUMF1(0·323); APLF(0·137); PIAS1(0·08); CRBN(0·045); DIS3(0·012); TBC1D22B(0·008)
Stomach	0·703	0·840	-0·014	0·674	1	TOX3(3·123); HOXB6(1·313); HOXB5(1·312); SUMF1(1·202); AAGAB(1·157); HOXB3(1·083); HOXB8(0·931); PRMT6(0·895); SETBP1(0·66); HOXB7(0·652); TRNT1(0·626); ETAA1(0·591); MEIS1(0·556); CMTR1(0·533); CALML4(0·519); ASTE1(0·518); BORA(0·507); HOXB2(0·495); PIAS1(0·488); PIK3R4(0·346); CLN6(0·325); DIS3(0·294); IQCH(0·146); TBC1D22B(0·085); SMAD3(0·035)
Skin - Sun Exposed (Lower leg)	0·691	0·840	-0·042	0·601	1	TBC1D22B(1·833); APLF(1·246); SUMF1(0·743); ATP2C1(0·728); PIK3R4(0·668); GLO1(0·588); C15orf41(0·552); HOXB7(0·529); CLN6(0·526); MAP2K5(0·517); DPH6(0·498); BTBD9(0·486); CMTR1(0·42); PIAS1(0·412); SKOR1(0·342); SMAD3(0·325); C15orf61(0·316); BORA(0·166); DIS3(0·161); RNF8(0·153); PKP4(0·122); HOXB2(0·105); ETAA1(0·099); HOXB3(0·043)
Adipose - Visceral (Omentum)	0·89	0·909	-0·399	0·35	1	HOXB7(4·796); HOXB6(2·909); HOXB8(2·513); HOXB5(2·273); HOXB3(2·031); HOXB2(2·007); SMAD3(1·167); DACH1(1·108); MEIS1(0·988); SUMF1(0·982); HOXB9(0·809); DIS3(0·755); ATP2C1(0·736); TRNT1(0·659); PIK3R4(0·508); COL6A6(0·494); NTNG1(0·459); SETBP1(0·412); SEMA6D(0·404); CALML4(0·355); PRMT6(0·294); CRBN(0·28); APLF(0·277); PKP4(0·226); BORA(0·167); CLN6(0·11); NEK11(0·087); C15orf61(0·066); ETAA1(0·028); MDGA1(0·004)
Skin - Not Sun Exposed (Suprapubic)	0·869	0·906	-0·761	0·158	1	TBC1D22B(1·791); APLF(1·351); ATP2C1(0·982); SUMF1(0·784); GLO1(0·646); HOXB7(0·602); SMAD3(0·484); CLN6(0·451); PIK3R4(0·449); MAP2K5(0·401); DPH6(0·383); C15orf61(0·38); PKP4(0·374); BORA(0·373); RNF8(0·312); C15orf41(0·308); SKOR1(0·302); MEIS1(0·265); ETAA1(0·256); PIAS1(0·224); CMTR1(0·173); HOXB3(0·119); BTBD9(0·118); HOXB2(0·105); DIS3(0·091); CALML4(0·078); COL6A6(0·071); MDGA1(0·023)
Whole Blood	0·853	0·906	-0·783	0·871	1	PIAS1(1·297); CALML4(0·919); DACH1(0·712); SUMF1(0·486); CLN6(0·445); BORA(0·38); ASTE1(0·371); CCDC167(0·357); HOXB9(0·312); C7orf62(0·299); CRBN(0·253); HOXB2(0·249); TBC1D22B(0·222); ZNF804B(0·154); CMTR1(0·152); MDGA1(0·082); BTBD9(0·037); AAGAB(0·02)
Liver	0·962	0·962	-1·395	0·742	1	TOX3(1·307); CCDC167(1·117); CLN6(0·462); SUMF1(0·449); PTPRD(0·434); PTPRD(0·434); CALML4(0·388); COL6A6(0·323); PRMT6(0·299); ASTE1(0·294); PIK3R4(0·216); GLO1(0·201); TRNT1(0·058); SMAD3(0·035); SETBP1(0·028); DACH1(0·006); ETAA1(0·004)

Supplementary Table 12: Results of tissue enrichment analysis using DEPICT. MeSH term, Medical Subject Heading term for the tissue or cell type annotation; MeSH first level term, description of the tissue or cell type annotation;MeSH second level term, more general description of the tissue or cell type annotation; Nominal p value, nominal enrichment p value of tissue/cell type annotation (Null hypothesis: Genes in associated are not highly expressed in the given tissue or cell type); False discovery rate, estimated false discovery rate of the enrichment P value for the tissue or cell type.

Name	MeSH first level term	MeSH second level term	Nominal p value	False discovery rate
**Mesencephalon**	Nervous System	Central Nervous System	0·03	>=0·2
**Diencephalon**	Nervous System	Central Nervous System	0·06	>=0·2
**Rectum**	Digestive System	Gastrointestinal Tract	0·07	>=0·2
**Uterus**	Urogenital System	Genitalia	0·07	>=0·2
**Brain Stem**	Nervous System	Central Nervous System	0·07	>=0·2
**Serum**	Hemic and Immune Systems	Blood	0·07	>=0·2
**Retina**	Sense Organs	Eye	0·08	>=0·2
**Frontal Lobe**	Nervous System	Central Nervous System	0·08	>=0·2
**Heart Ventricles**	Cardiovascular System	Heart	0·08	>=0·2
**Cicatrix**	Tissues	Connective Tissue	0·08	>=0·2
**Granulation Tissue**	Tissues	Connective Tissue	0·08	>=0·2
**Muscles**	Tissues	Muscles	0·09	>=0·2
**Cecum**	Digestive System	Gastrointestinal Tract	0·09	>=0·2
**Colon Sigmoid**	Digestive System	Gastrointestinal Tract	0·09	>=0·2
**Endometrium**	Urogenital System	Genitalia	0·09	>=0·2
**Embryoid Bodies**	Cells	Stem Cells	0·09	>=0·2

Supplementary Table 13: Results of genetic correlation analysis of RLS versus traits in LD-hub. Trait, trait tested for genetic correlation with RLS; PMID, Pubmed ID of publication that contains the data; Category, trait category; Ethnicity, ethnicity of the population in which the GWAS on the trait was performed; r_g, genetic correlation coefficient; se, standard error of the genetic correlation coefficient estimate; p, p value of genetic correlation.

Trait	PMID	Category	Ethnicity	r_g	se	p
Years of schooling 2016	27225129	education	European	-0·1041	0·049	0·0337
College completion	23722424	education	European	-0·1565	0·0833	0·0603
Childhood IQ	23358156	education	European	-0·1924	0·1192	0·1064
Years of schooling 2013	23722424	education	European	-0·1231	0·079	0·119
Years of schooling (proxy cognitive performance)	25201988	education	European	-0·1212	0·0799	0·1295
Ferritin	25352340	metal	European	-0·1687	0·3725	0·6506
Transferrin	25352340	metal	European	0·0713	0·1738	0·6816
Neuroticism	27089181	personality	European	0·3104	0·0628	7·73E-07
Neuroticism	24828478	personality	European	0·5669	0·1786	0·0015
Neo-conscientiousness	21173776	personality	European	-0·1513	0·164	0·3563
Neo-openness to experience	21173776	personality	European	-0·0815	0·1328	0·5392
Depressive symptoms	27089181	psychiatric	European	0·2787	0·0872	0·0014
PGC cross-disorder analysis	23453885	psychiatric	European	0·1454	0·082	0·0764
Subjective well being	27089181	psychiatric	European	-0·1262	0·0849	0·1372
Major depressive disorder	22472876	psychiatric	European	0·1471	0·1	0·1413
Amyotrophic lateral sclerosis	27455348	psychiatric	European	0·2008	0·1529	0·1891
Alzheimers disease	24162737	psychiatric	European	-0·1642	0·1349	0·2235
Anorexia Nervosa	24514567	psychiatric	European	0·0668	0·0728	0·3588
Bipolar disorder	21926972	psychiatric	European	0·0701	0·092	0·4462
Parkinsons disease	19915575	psychiatric	European	-0·0653	0·1187	0·5824
Autism spectrum disorder	NA	psychiatric	European	-0·0561	0·1091	0·6073
Attention deficit hyperactivity disorder	20732625	psychiatric	European	0·0648	0·1408	0·6457
Number of children ever born	27798627	reproductive	European	0·2241	0·0843	0·0079
Age of first birth	27798627	reproductive	European	-0·1055	0·0667	0·1139
Age at Menarche	25231870	reproductive	European	-0·0072	0·0528	0·8913
Age at Menopause	26414677	reproductive	European	-0·006	0·0666	0·9284
Chronotype	27494321	sleeping	European	-0·1178	0·0622	0·0583
Sleep duration	27494321	sleeping	European	-0·1228	0·0844	0·1458

Supplementary Table 14: Overlap of RLS risk loci with NHGRI-EBI GWAS catalog signals. RLS risk locus definition refers to appendix p 4, "Identification of genome-wide significant SNPs"; GWAS catalog, data based on NHGRI-EBI Catalog of published genome-wide association studies version v1.0.1 with a p value-based cutoff of $p \leq 5 \times 10^{-5}$; RLS meta-analysis, association statistics of RLS meta-analysis; Effect (ßeta) refers to allele 1.

RLS risk locus				GWAS catalog						RLS meta-analysis				
**Locus lead SNP rsID**	**Chr**	**Start**	**End**	**rsID**	**Risk allele**	**p value**	**OR**	**PubmedID**	**Traits/Diseases**	**Allele 1**	**Allele 2**	**Effect (ßeta)**	**SE**	**p value**
rs12046503	1	107058351	107431656	rs17440619	NA	2·00E-06	2·79	26116289	Dysphagia	t	c	0·0043	0·0188	0·8189
rs12046503	1	107058351	107431656	rs12097821	NA	6·00E-10	1·25	22197933	Non-obstructive_azoospermia	t	g	-0·0965	0·0208	3·55E-06
rs10208712	2	4015981	4147979	rs6708110	G	8·00E-07	0·66	26252872	Cognitive_decline_rate_in_late_mild_cognitive_impairment	NA	NA	NA	NA	NA
rs113851554	2	66483748	67022234	rs2901879	NA	7·00E-07	1·11	24743840	Colorectal_cancer_(diet_interaction)	t	c	0·0366	0·0139	0·008511
rs113851554	2	66483748	67022234	rs3891585	A	1·00E-11	2·13	23139255	PR_interval	a	g	0·0942	0·0143	4·13E-11
rs113851554	2	66483748	67022234	rs11897119	C	5·00E-11	1·36	20062060	PR_interval	t	c	0·0632	0·0143	9·57E-06
rs113851554	2	66483748	67022234	rs10865355	A	3·00E-09	2·89	21347284	PR_interval	a	g	0·0935	0·0143	5·69E-11
rs113851554	2	66483748	67022234	rs11678354	A	5·00E-09	1·26	24850809	PR_segment	a	t	-0·0502	0·015	0·000818
rs113851554	2	66483748	67022234	rs2300478	G	3·00E-49	1·68	21779176	Restless_legs_syndrome	NA	NA	NA	NA	NA
rs113851554	2	66483748	67022234	rs10490193	A	7·00E-06	4·50	25378659	Very_long-chain_saturated_fatty_acid_levels_(fatty_acid_20:0)	a	c	-0·0226	0·0175	0·1954
rs1820989	2	68036723	68149107	rs6747972	A	9·00E-11	1·23	21779176	Restless_legs_syndrome	NA	NA	NA	NA	NA
rs61192259	6	38190303	38667852	rs4565302	C	5·00E-06	2·94	25353672	Mammographic_density	t	c	-0·0096	0·02	0·6302
rs61192259	6	38190303	38667852	rs77928651	A	1·00E-06	0·25	26634245	Post_bronchodilator_FEV1/FVC_ratio	NA	NA	NA	NA	NA
rs61192259	6	38190303	38667852	rs139632137	A	2·00E-06	0·28	26634245	Post_bronchodilator_FEV1/FVC_ratio	NA	NA	NA	NA	NA
rs61192259	6	38190303	38667852	rs114407411	A	2·00E-07	0·13	26634245	Post_bronchodilator_FEV1/FVC_ratio_in_COPD	NA	NA	NA	NA	NA
rs61192259	6	38190303	38667852	rs192452148	T	2·00E-07	0·13	26634245	Post_bronchodilator_FEV1/FVC_ratio_in_COPD	NA	NA	NA	NA	NA
rs61192259	6	38190303	38667852	rs150713181	G	4·00E-07	0·12	26634245	Post_bronchodilator_FEV1/FVC_ratio_in_COPD	NA	NA	NA	NA	NA
rs61192259	6	38190303	38667852	rs9357271	T	8·00E-22	1·47	21779176	Restless_legs_syndrome	NA	NA	NA	NA	NA
rs61192259	6	38190303	38667852	rs9296249	T	4·00E-18	1·67	17637780	Restless_legs_syndrome	NA	NA	NA	NA	NA
rs61192259	6	38190303	38667852	rs3923809	A	1·00E-17	1·90	17634447	Restless_legs_syndrome	NA	NA	NA	NA	NA

rs10952927	7	88310316	88551817	rs10248351	C	7·00E-06	1·84	22493691	Hypothyroidism	NA	NA	NA	NA	NA
rs1836229	9	8817244	8858043	rs1975197	A	3·00E-10	1·29	21779176	Restless_legs_syndrome	NA	NA	NA	NA	NA
rs62535767	9	9202034	9374664	rs4626664	A	6·00E-10	1·44	18660810	Restless_legs_syndrome	NA	NA	NA	NA	NA
rs996064	15	36066927	36379533	rs4923705	C	2·00E-06	1·51	20732626	Attention_deficit_hyperactivity_disorder	NA	NA	NA	NA	NA
rs996064	15	36066927	36379533	rs76065397	T	1·00E-06	0·40	26252872	Cognitive_decline_rate_in_late_mild_cognitive_impairment	t	c	0·0194	0·0564	0·7312
rs996064	15	36066927	36379533	rs10520045	T	7·00E-06	1·32	20673876	Major_depressive_disorder	NA	NA	NA	NA	NA
rs996064	15	36066927	36379533	rs1898036	NA	2·00E-07	7·73	22491018	Response_to_tocilizumab_in_rheumatoid_arthritis	t	c	-0·0241	0·0227	0·2881
rs111652004	15	47004724	47489575	rs16958536	NA	4·00E-06	1·66	24785509	Response_to_radiotherapy_in_cancer_(late_toxicity)	NA	NA	NA	NA	NA
rs868036	15	67470208	68533340	rs2241423	G	1·00E-18	0·13	20935630	Body_mass_index	NA	NA	NA	NA	NA
rs868036	15	67470208	68533340	rs16951275	T	2·00E-17	0·03	25673413	Body_mass_index	NA	NA	NA	NA	NA
rs868036	15	67470208	68533340	rs4776970	A	3·00E-07	0·03	24861553	Body_mass_index	NA	NA	NA	NA	NA
rs868036	15	67470208	68533340	rs448720	NA	5·00E-06	NA	20125193	Cognitive_performance	t	c	-0·09	0·0139	1·05E-10
rs868036	15	67470208	68533340	rs8032675	T	2·00E-13	0·04	25231870	Menarche_(age_at_onset)	t	c	-0·0046	0·0142	0·7456
rs868036	15	67470208	68533340	rs7359257	A	2·00E-06	1·70	21102462	Menarche_(age_at_onset)	a	c	0·0575	0·0141	4·35E-05
rs868036	15	67470208	68533340	rs997295	T	3·00E-09	0·03	25628336	Motion_sickness	t	g	-0·0826	0·0142	5·87E-09
rs868036	15	67470208	68533340	rs8028313	C	6·00E-13	1·08	23563607	Obesity	c	g	0·1257	0·0173	3·84E-13
rs868036	15	67470208	68533340	rs11637445	G	6·00E-06	1·49	26993346	Posterior_cortical_atrophy_and_Alzheimer's_disease	t	g	0·1121	0·0141	2·13E-15
rs868036	15	67470208	68533340	rs12593813	G	1·00E-22	1·41	21779176	Restless_legs_syndrome	NA	NA	NA	NA	NA
rs868036	15	67470208	68533340	rs338389	NA	3·00E-06	3·40	25866641	Survival_in_rectal_cancer	a	g	0·049	0·0139	0·0004148
rs868036	15	67470208	68533340	rs4776997	G	1·00E-06	NA	25648963	Verbal_declarative_memory	NA	NA	NA	NA	NA
rs45544231	16	52516607	52648731	rs3803662	A	2.00E-114	1·24	23535729	Breast_cancer	NA	NA	NA	NA	NA
rs45544231	16	52516607	52648731	rs3112612	T	4·00E-10	1·15	21263130	Breast_cancer	a	g	-0·0553	0·0142	9·77E-05
rs45544231	16	52516607	52648731	rs12922061	T	4·00E-10	1·23	24143190	Breast_cancer	t	c	-0·1482	0·0169	1·81E-18
rs45544231	16	52516607	52648731	rs4784227	T	3·00E-09	1·38	25327703	Breast_cancer	NA	NA	NA	NA	NA
rs45544231	16	52516607	52648731	rs2193094	NA	2·00E-07	1·34	25956309	Breast_cancer	t	g	0·0417	0·0139	0·002761
rs45544231	16	52516607	52648731	rs4784223	NA	6·00E-21	1·27	24493630	Breast_cancer_(early_onset)	a	g	0·1143	0·0159	7·36E-13
rs45544231	16	52516607	52648731	rs3803662	NA	4·00E-15	1·50	23001122	Breast_cancer_(male)	NA	NA	NA	NA	NA
rs45544231	16	52516607	52648731	rs3104767	G	9·00E-19	1·35	21779176	Restless_legs_syndrome	t	g	-0·0996	0·0143	2·81E-12
rs12450895	17	46666937	46772821	rs8074700	A	1·00E-06	NA	23517042	Body_mass_index_in_non-asthmatics	a	c	-0·0229	0·0157	0·1436

rs12450895	17	46666937	46772821	rs2326017	T	2·00E-06	NA	23517042	Body_mass_index_in_non-asthmatics	t	c	0·0711	0·0151	2·53E-06
rs12450895	17	46666937	46772821	rs11079830	A	2·00E-06	0·03	26604143	Childhood_body_mass_index	a	g	-0·0085	0·0142	0·5504
rs12450895	17	46666937	46772821	rs2326017	NA	3·00E-07	NA	19734545	Cognitive_performance	t	c	0·0711	0·0151	2·53E-06
rs12450895	17	46666937	46772821	rs3096644	G	6·00E-06	0·08	23934736	Metabolite_levels_(Dihydroxy_docosatrienoic_acid)	t	g	0·0678	0·0146	3·61E-06
rs12450895	17	46666937	46772821	rs9299	NA	4·00E-09	1·14	22484627	Obesity	t	c	0·0431	0·0145	0·003017
rs12450895	17	46666937	46772821	rs9890514	NA	4·00E-06	0·01	22550155	Platelet_thrombus_formation	a	c	-0·0368	0·0342	0·2823

Supplementary Table 15: Results of gene prioritization analysis using DEPICT. Chromosome and position, chromosome and boundaries of the associated locus as defined by DEPICT (HG19/GRCh37 genome build); GWAS p value, P value in discovery stage meta-analysis; Gene symbol, Hugo gene symbol, Nominal P value, nominal gene prioritization P value; Gene closest to lead SNP, indication whether the gene is the gene nearest to the associated SNP (Yes, 1; No, 0); Top cis eQTL SNP, SNPs that are eQTLs for the gene in whole blood in dataset PMID 24013639).

Chromosome and position	GWAS p value	Gene symbol	Nominal p value	Gene closest to lead SNP	Top cis eQTL SNP
chr1:107599267-107601916	3·35E-31	*PRMT6*	0·92	TRUE	rs1623927;rs1730858
chr13:72012098-72441330	3·94E-08	*DACH1*	0·69	TRUE	rs9542624
chr15:47476298-48066420	1·05E-10	*SEMA6D*	0·72	TRUE	rs1377673;rs532598
chr15:67547138-68131217	1·11E-48	*C15orf61*	0·33	FALSE	rs17240463
chr15:67547138-68131217	1·11E-48	*IQCH*	0·75	FALSE	-
chr15:67547138-68131217	1·11E-48	*MAP2K5*	0·48	TRUE	rs12902812;rs12441715
chr16:52471918-52640847	4·78E-48	*TOX3*	0·81	TRUE	rs187680;rs12925035
chr17:46627247-46716647	4·88E-08	*HOXB6*	0·65	FALSE	rs1343832
chr17:46627247-46716647	4·88E-08	*HOXB8*	0·63	FALSE	-
chr17:46627247-46716647	4·88E-08	*HOXB9*	0·56	FALSE	-
chr18:42260138-42648475	1·37E-10	*SETBP1*	0·58	TRUE	rs1036929;rs4890486
chr2:159023162-159539391	3·19E-14	*CCDC148*	0·53	TRUE	-
chr2:159023162-159539391	3·19E-14	*PKP4*	0·71	FALSE	rs1111212;rs2711085
chr2:66660584-66799890	1·10E-180	*MEIS1*	0·92	TRUE	rs2280334
chr20:62783144-62873604	3·36E-14	*MYT1*	0·74	TRUE	-
chr3:130569439-130735556	4·37E-13	*ATP2C1*	0·50	TRUE	rs2669858;rs885076
chr3:3190676-3221394	5·39E-14	*CRBN*	0·88	TRUE	rs1672767
chr6:37450696-37467700	6·44E-11	*CCDC167*	0·39	TRUE	rs1757173
chr6:37598455-37667082	6·84E-10	*MDGA1*	0·41	TRUE	rs6938061
chr6:37787275-38670917	1·36E-78	*BTBD9*	0·60	TRUE	-
chr6:37787275-38670917	1·36E-78	*GLO1*	0·42	FALSE	rs1781735
chr6:37787275-38670917	1·36E-78	*ZFAND3*	0·65	TRUE	rs17648634
chr7:88388682-88966346	1·86E-15	*C7orf62*	0·42	FALSE	-
chr7:88388682-88966346	1·86E-15	*ZNF804B*	0·40	TRUE	-
chr9:8314246-10612723	1·94E-15	*PTPRD*	0·78	FALSE	rs7864527;rs324457;rs4742560;rs7867067

3. Supplementary Figures

Supplementary Figure 1: Regional association plots of all genome-wide significant association signals of the discovery stage meta-analysis, see pages 44 to 86

Supplementary Figure 2: Density plot for polygenic risk score showing distribution of scores in EU-RLS-GENE study

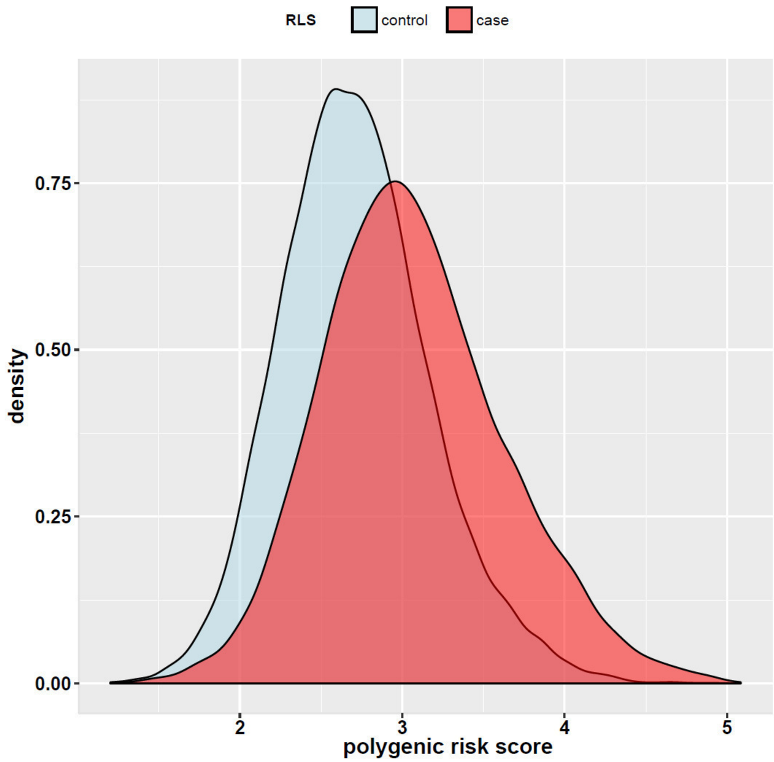

Red color represents cases and light-blue color controls; the x-axis represents the polygenic risk score and the y-axis represents the probability density of the polygenic risk score, which is estimated by 'Gaussian' kernel implemented in the ggplot2 R package.

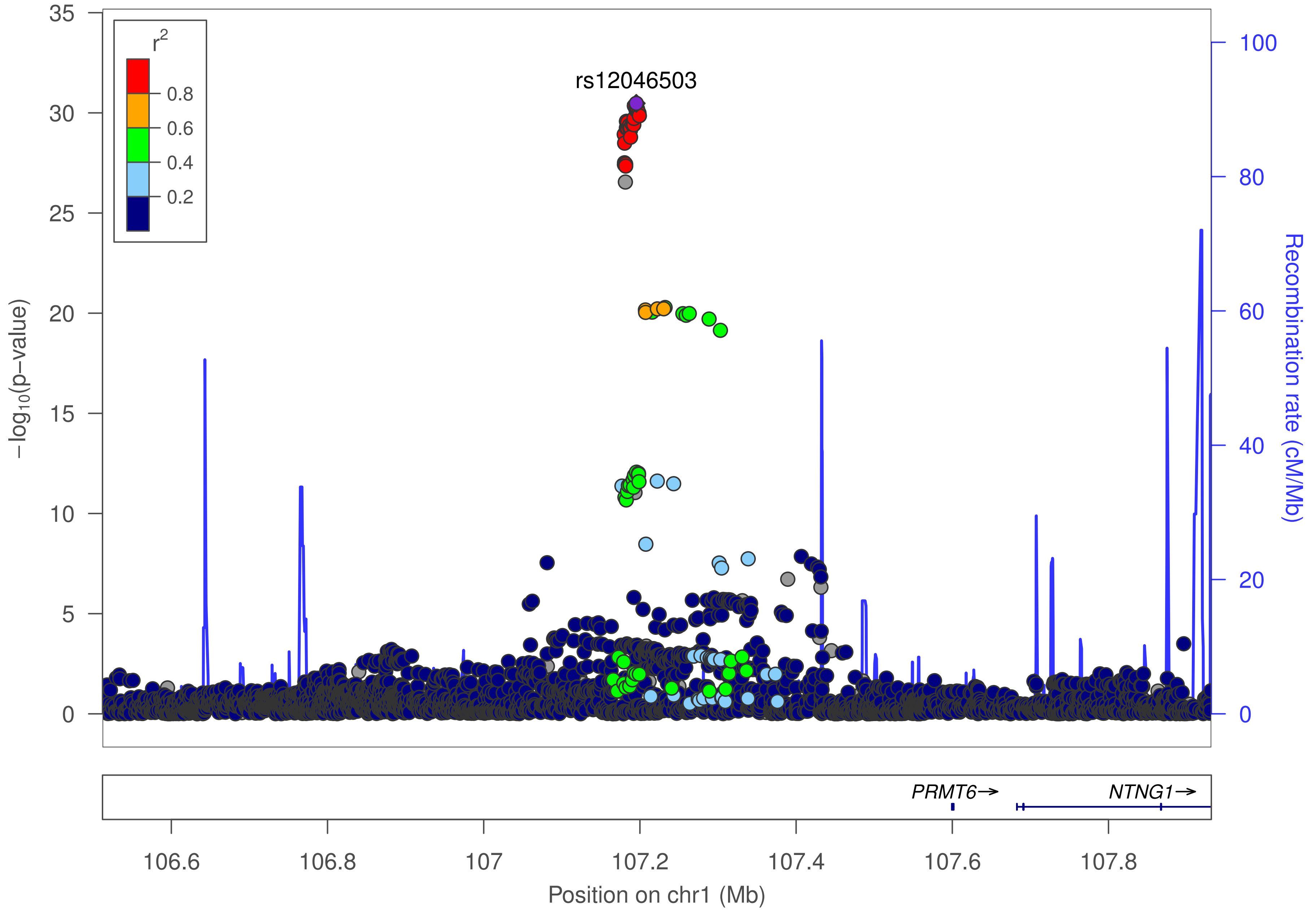
r2
0.8
0.6
0.4
0.2
rs12046503
−log10(p−value)
Recombination rate (cM/Mb)
35
30
25
20
15
10
5
0
100
80
60
40
20
0
PRMT6→
NTNG1→
106.6
106.8
107
107.2
107.4
107.6
107.8
Position on chr1 (Mb)

date:	Mon Nov 21 15:05:53 2016
build:	hg19
display range:	chr1:106511892−107931656 [106511892−107931656]
hilite range:	0 – 0 [0 – 0]
reference SNP:	chr1:107195339
number of SNPs plotted:	3495
min P−value:	3.35E−31 [chr1:107195339]
max P−value:	10E−1 [chr1:107558361]

Make more plots at http://csg.sph.umich.edu/locuszoom/

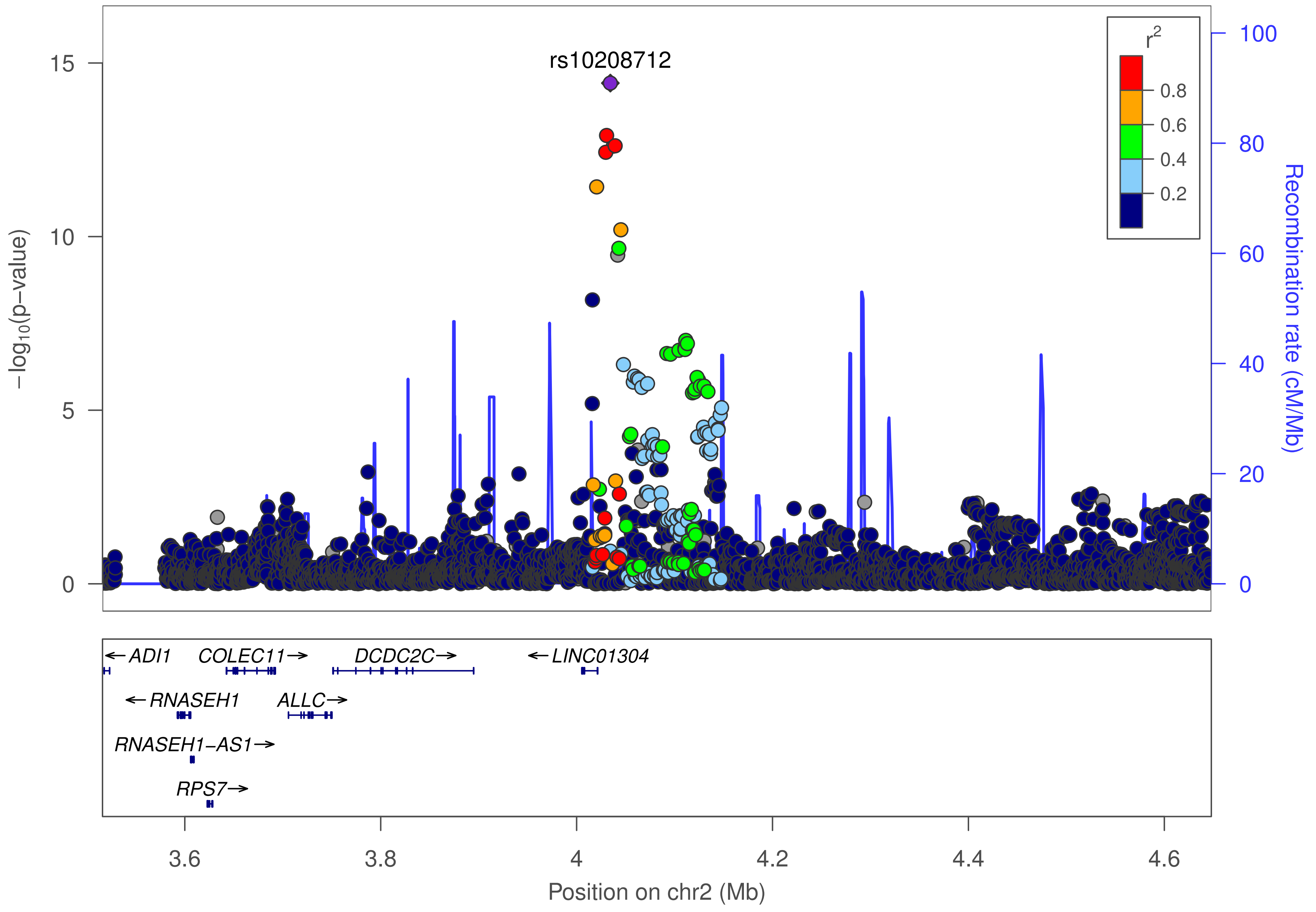

rs10208712
r^2
0.8
0.6
0.4
0.2
−log10(p−value)
Recombination rate (cM/Mb)
← ADI1
COLEC11 →
DCDC2C →
← LINC01304
← RNASEH1
ALLC →
RNASEH1−AS1 →
RPS7 →
3.6
3.8
4
4.2
4.4
4.6
Position on chr2 (Mb)

date:	Mon Nov 21 15:06:37 2016
build:	hg19
display range:	chr2:3515981−4647979 [3515981−4647979]
hilite range:	0 – 0 [0 – 0]
reference SNP:	chr2:4034446
number of SNPs plotted:	3456
min P−value:	3.79E−15 [chr2:4034446]
max P−value:	10E−1 [chr2:3797626]

Make more plots at http://csg.sph.umich.edu/locuszoom/

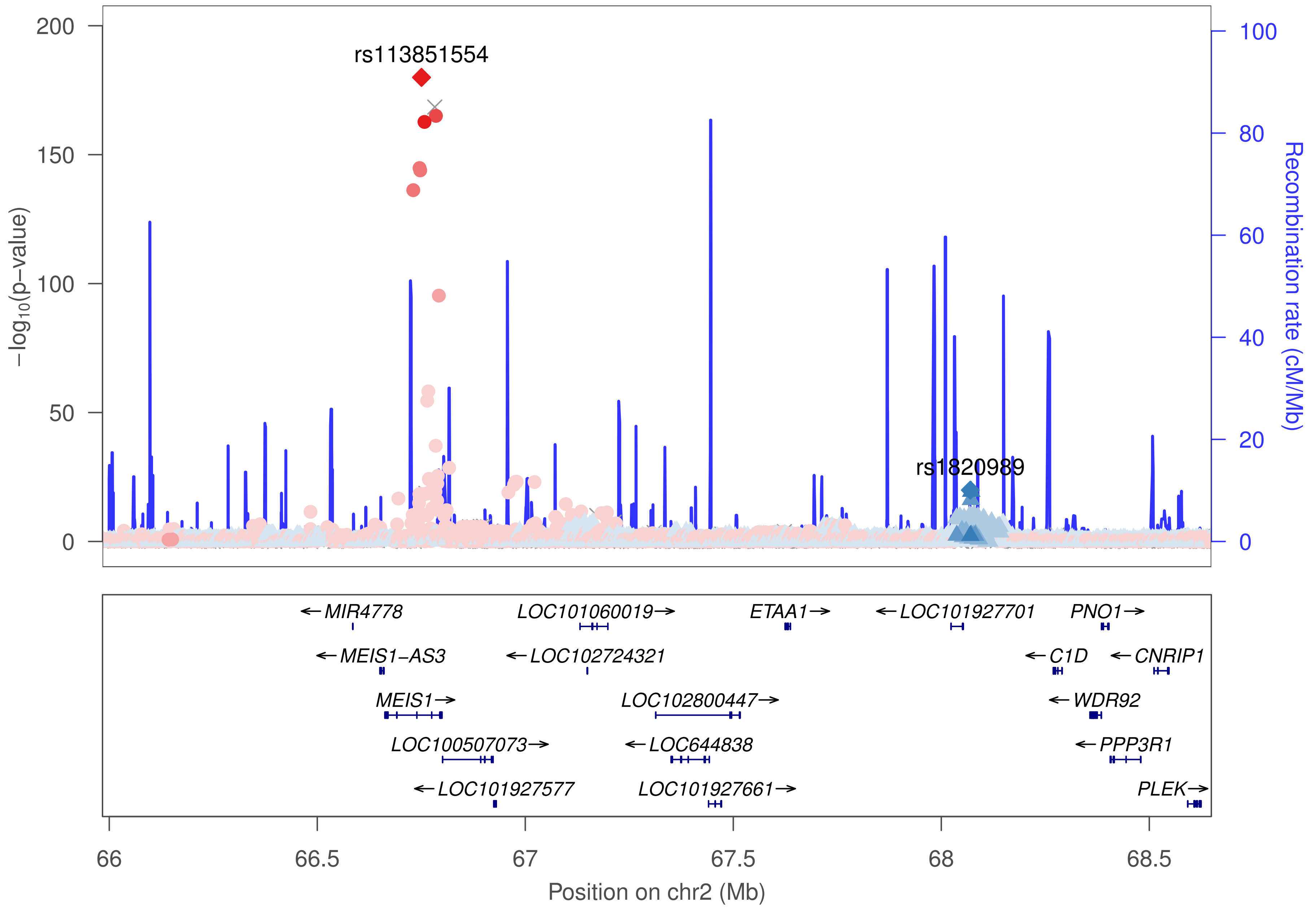
rs113851554
rs1820989
−log10(p−value)
Recombination rate (cM/Mb)
200
150
100
50
0
100
80
60
40
20
0
MIR4778
LOC101060019
ETAA1
LOC101927701
PNO1
MEIS1−AS3
LOC102724321
C1D
CNRIP1
MEIS1
LOC102800447
WDR92
LOC100507073
LOC644838
PPP3R1
LOC101927577
LOC101927661
PLEK
66
66.5
67
67.5
68
68.5
Position on chr2 (Mb)

date:	Mon Nov 21 15:09:47 2016
build:	hg19
display range:	chr2:65983748−68649107 [65983748−68649107]
hilite range:	0 – 0 [0 – 0]
reference SNP:	chr2:66750564
number of SNPs plotted:	7499
min P−value:	1.1E−180 [chr2:66750564]
max P−value:	10E−1 [chr2:66312334]

Make more plots at http://csg.sph.umich.edu/locuszoom/

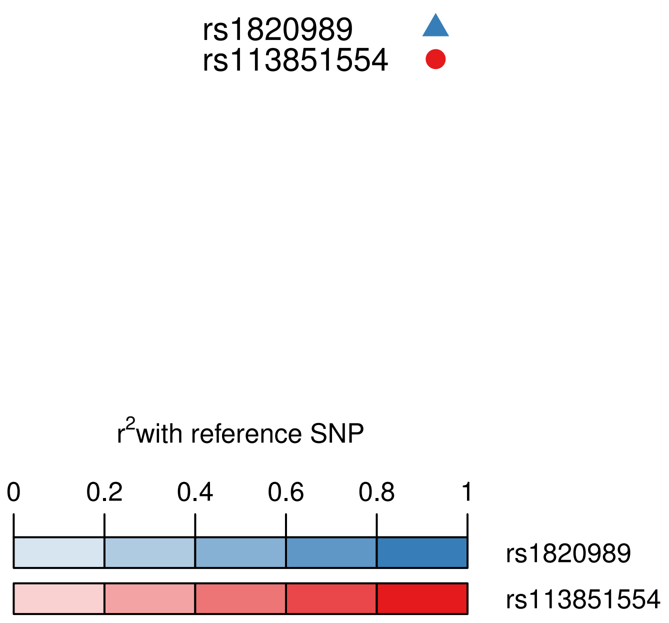
rs1820989
rs113851554
r2 with reference SNP
0
0.2
0.4
0.6
0.8
1
rs1820989
rs113851554

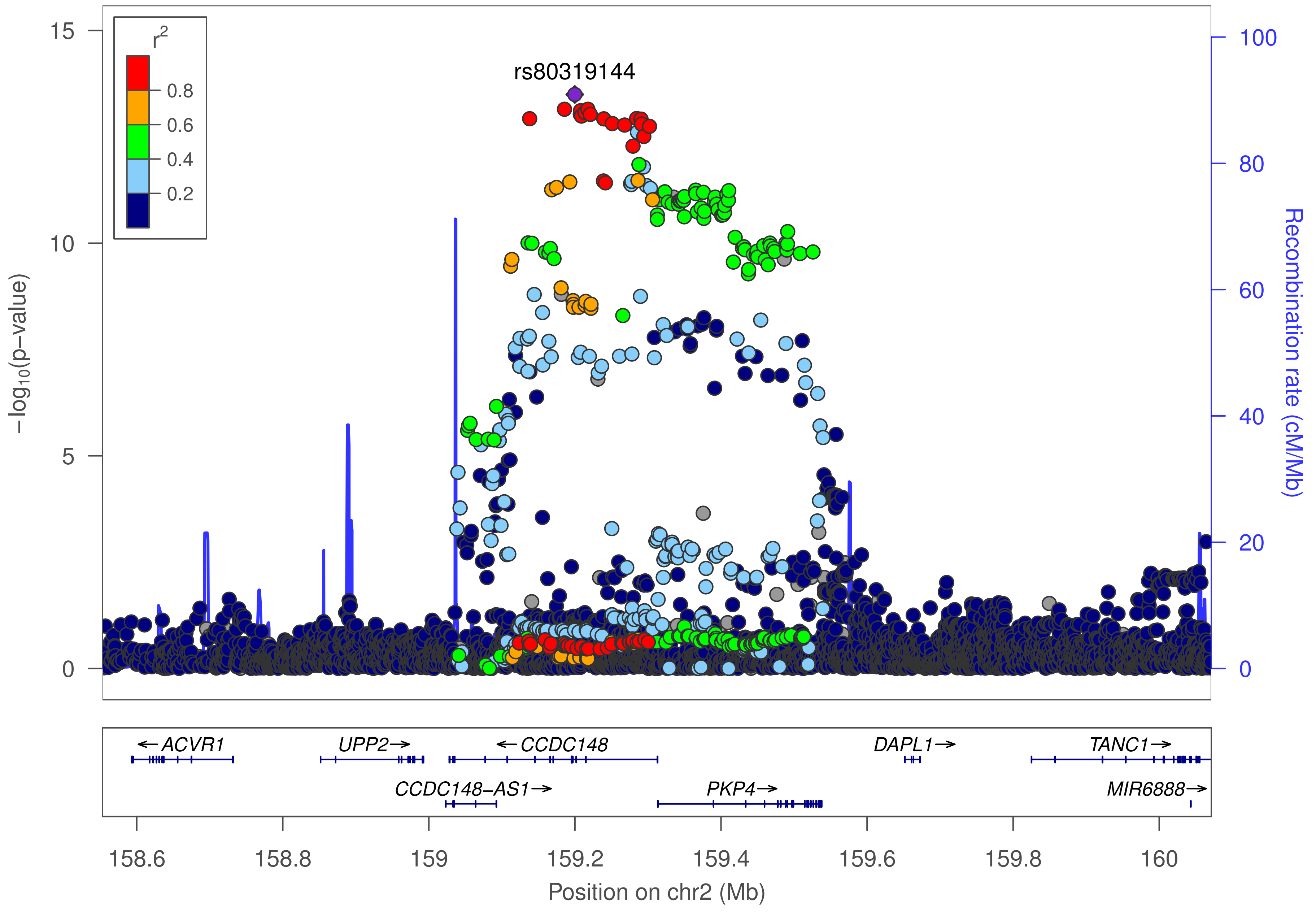

rs80319144
r2
0.8
0.6
0.4
0.2
−log10(p−value)
Recombination rate (cM/Mb)
ACVR1
UPP2
CCDC148
CCDC148−AS1
PKP4
DAPL1
TANC1
MIR6888
158.6
158.8
159
159.2
159.4
159.6
159.8
160
Position on chr2 (Mb)

date:	Mon Nov 21 15:10:25 2016
build:	hg19
display range:	chr2:158553112−160071393 [158553112−160071393]
hilite range:	0 − 0 [0 − 0]
reference SNP:	chr2:159199835
number of SNPs plotted:	4411
min P−value:	3.19E−14 [chr2:159199835]
max P−value:	10E−1 [chr2:159006873]

Make more plots at http://csg.sph.umich.edu/locuszoom/

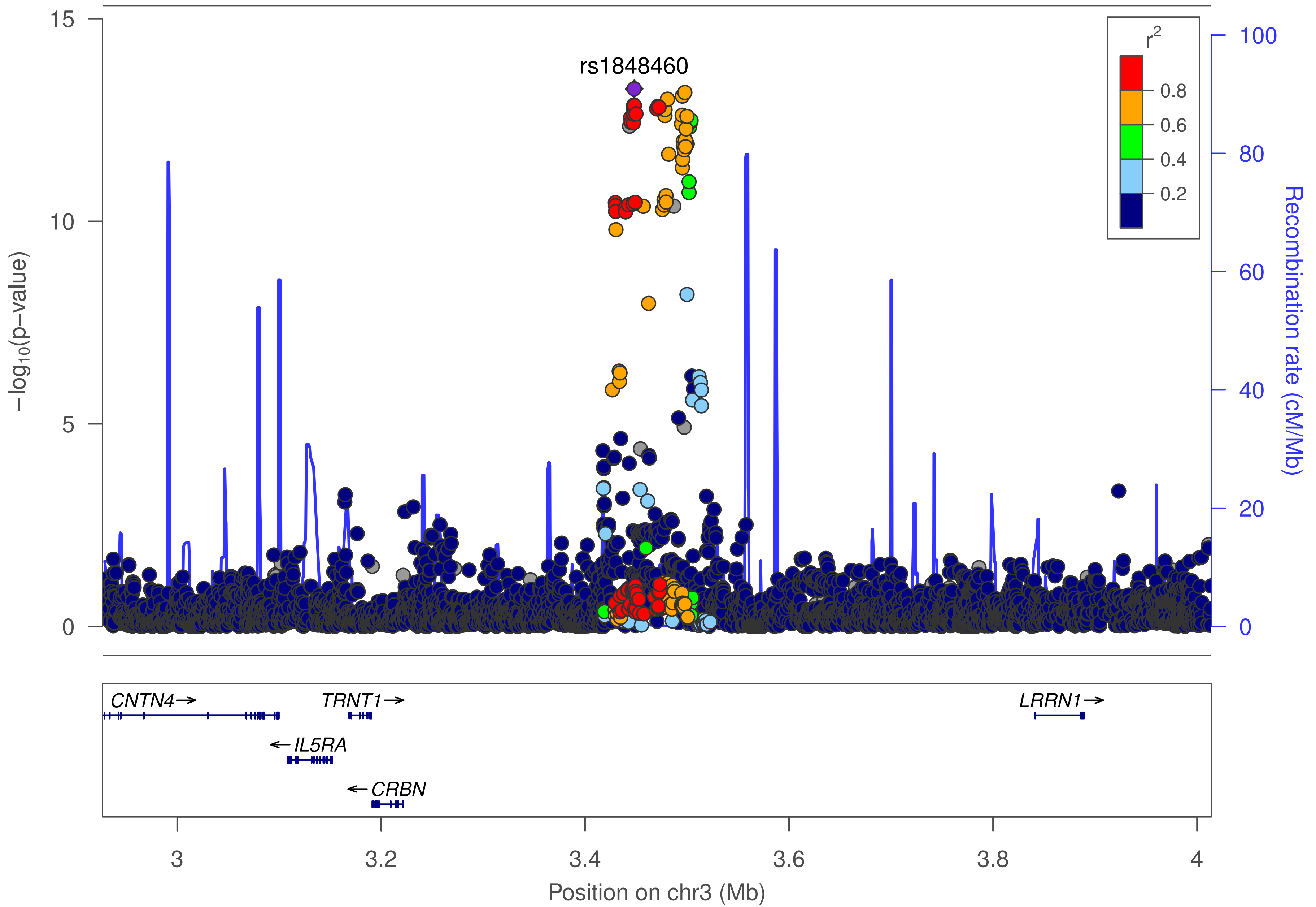

rs1848460
r2
0.8
0.6
0.4
0.2
15
10
5
0
100
80
60
40
20
0
−log10(p−value)
Recombination rate (cM/Mb)
CNTN4→
TRNT1→
LRRN1→
←IL5RA
←CRBN
3
3.2
3.4
3.6
3.8
4
Position on chr3 (Mb)

date:	Mon Nov 21 15:10:52 2016
build:	hg19
display range:	chr3:2926796−4014090 [2926796−4014090]
hilite range:	0 – 0 [0 – 0]
reference SNP:	chr3:3448144
number of SNPs plotted:	4113
min P−value:	5.39E−14 [chr3:3448144]
max P−value:	9.99E−1 [chr3:3003895]

Make more plots at http://csg.sph.umich.edu/locuszoom/

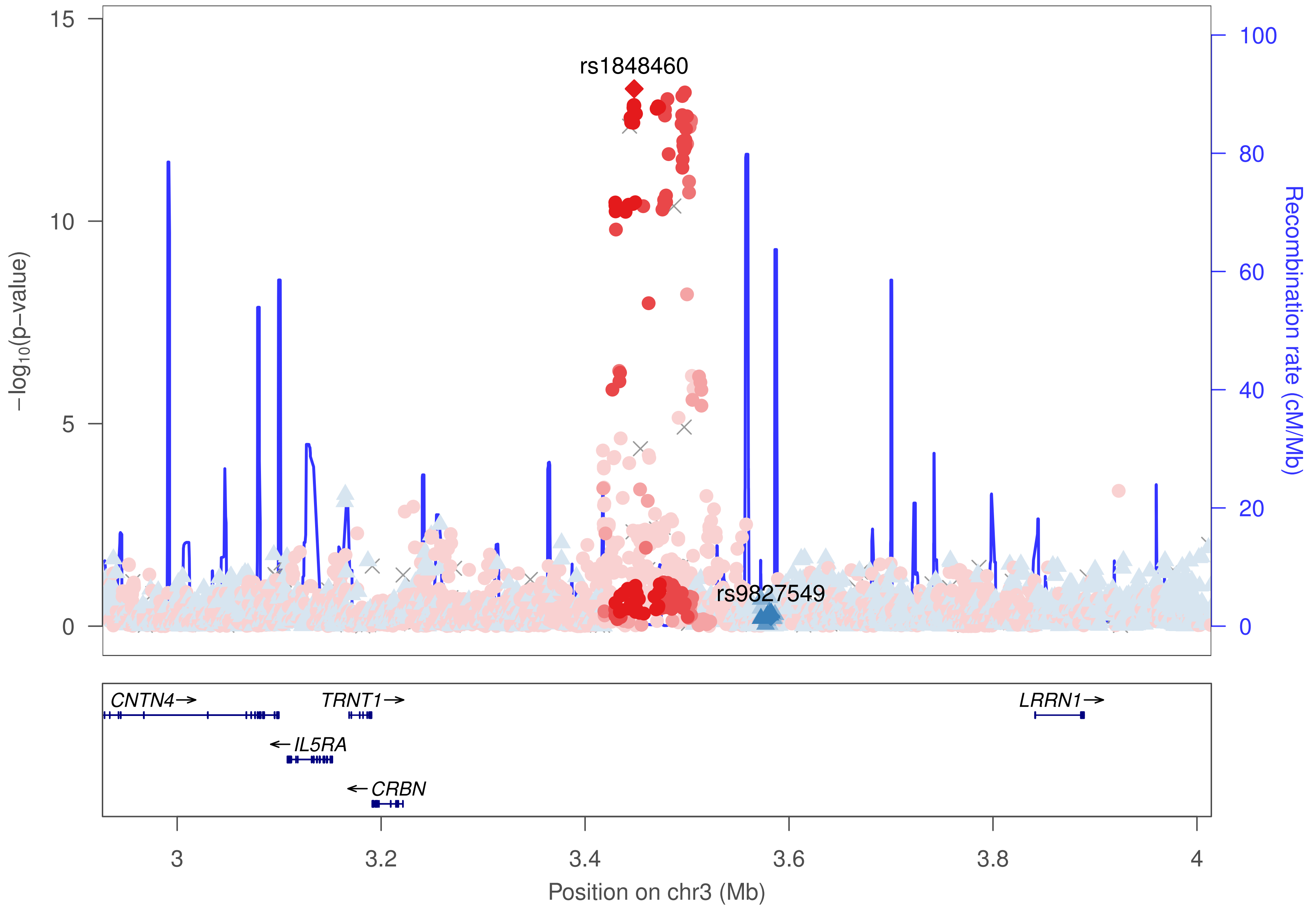
rs1848460
rs9827549
−log10(p−value)
Recombination rate (cM/Mb)
15
10
5
0
100
80
60
40
20
0
CNTN4→
TRNT1→
←IL5RA
←CRBN
LRRN1→
3
3.2
3.4
3.6
3.8
4
Position on chr3 (Mb)

date:	Thu Dec 15 14:51:19 2016
build:	hg19
display range:	chr3:2926796−4014090 [2926796−4014090]
hilite range:	0 – 0 [0 – 0]
reference SNP:	chr3:3448144
number of SNPs plotted:	4113
min P−value:	5.39E−14 [chr3:3448144]
max P−value:	9.99E−1 [chr3:3003895]

Make more plots at http://csg.sph.umich.edu/locuszoom/

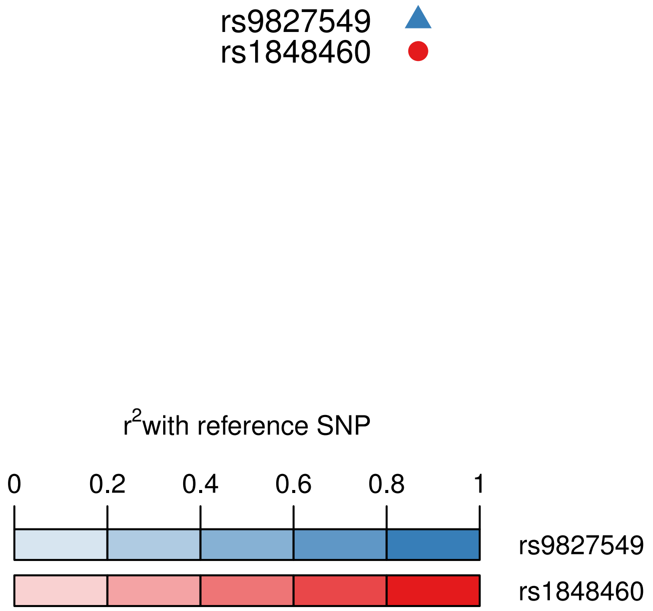
rs9827549
rs1848460
r^2 with reference SNP
0
0.2
0.4
0.6
0.8
1
rs9827549
rs1848460

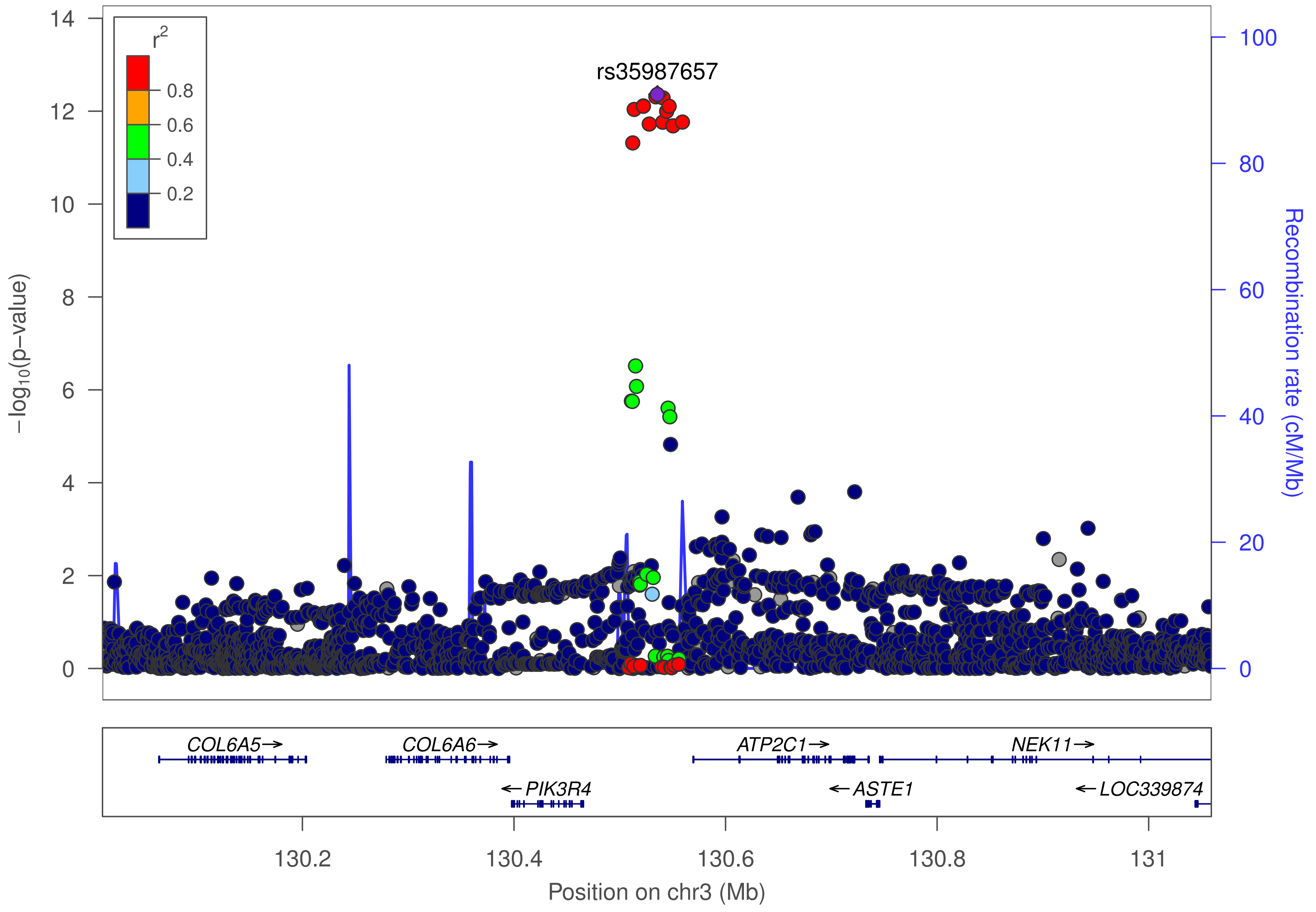

rs35987657
r2
0.8
0.6
0.4
0.2
−log10(p−value)
Recombination rate (cM/Mb)
COL6A5→
COL6A6→
ATP2C1→
NEK11→
←PIK3R4
←ASTE1
←LOC339874
130.2
130.4
130.6
130.8
131
Position on chr3 (Mb)

date:	Mon Nov 21 15:11:17 2016
build:	hg19
display range:	chr3:130011025−131059230 [130011025−131059230]
hilite range:	0 – 0 [0 – 0]
reference SNP:	chr3:130535567
number of SNPs plotted:	2187
min P−value:	4.37E−13 [chr3:130535567]
max P−value:	1E0 [chr3:130961422]

Make more plots at http://csg.sph.umich.edu/locuszoom/

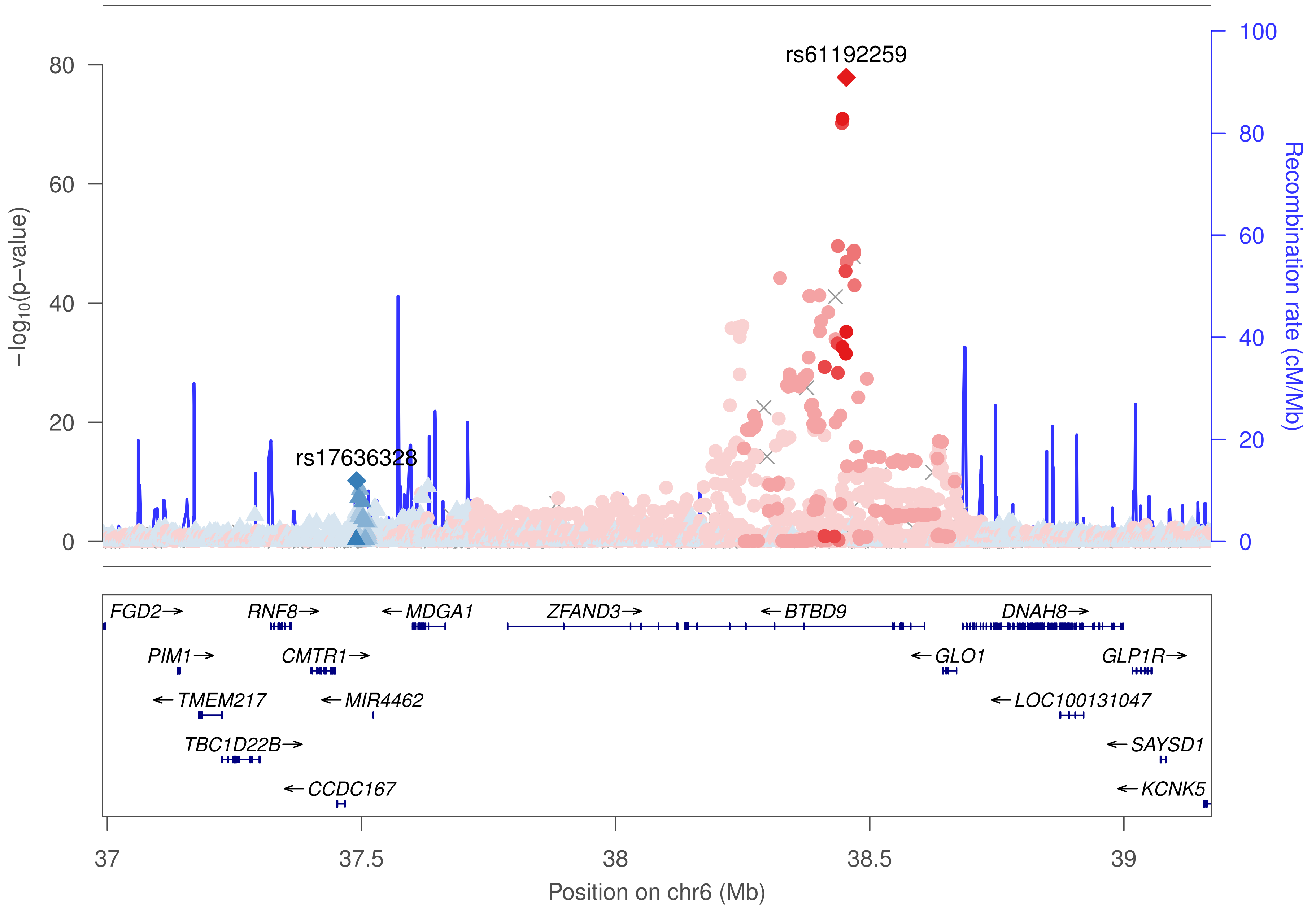
rs61192259
rs17636328
$-\log_{10}$(p−value)
Recombination rate (cM/Mb)
0
20
40
60
80
100
FGD2→
RNF8→
←MDGA1
ZFAND3→
←BTBD9
DNAH8→
PIM1→
CMTR1→
←GLO1
GLP1R→
←TMEM217
←MIR4462
←LOC100131047
TBC1D22B→
←SAYSD1
←CCDC167
←KCNK5
37
37.5
38
38.5
39
Position on chr6 (Mb)

date:	Mon Nov 21 15:13:11 2016
build:	hg19
display range:	chr6:36990531−39172079 [36990531−39172079]
hilite range:	0 – 0 [0 – 0]
reference SNP:	chr6:38453962
number of SNPs plotted:	5937
min P−value:	1.36E−78 [chr6:38453962]
max P−value:	1E0 [chr6:37555402]

Make more plots at http://csg.sph.umich.edu/locuszoom/

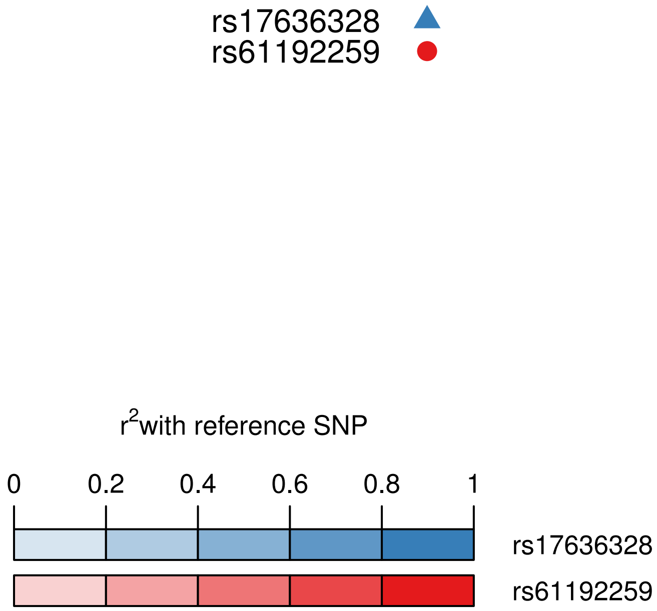

rs17636328
rs61192259
r2 with reference SNP
0
0.2
0.4
0.6
0.8
1
rs17636328
rs61192259

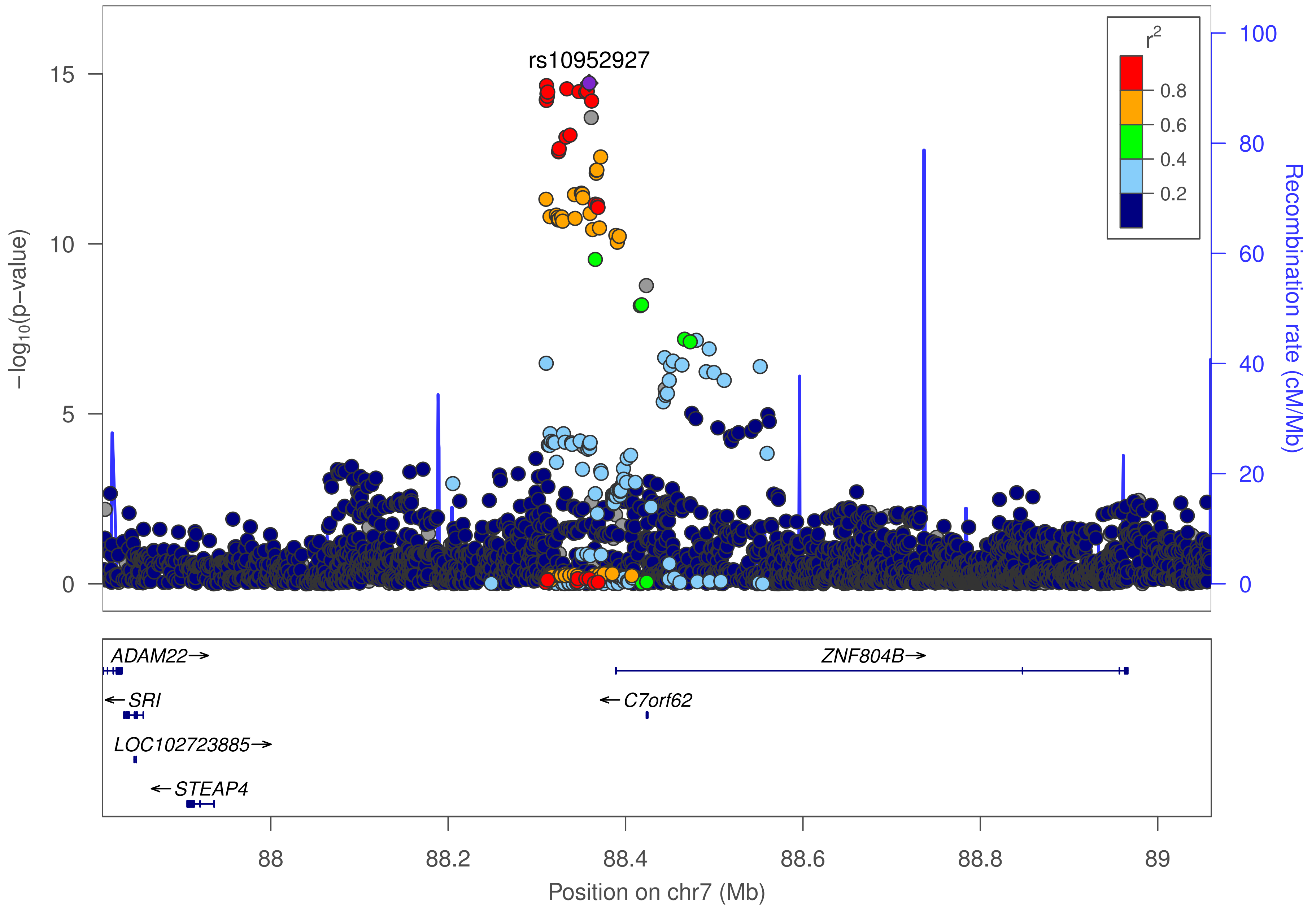

rs10952927
r²
0.8
0.6
0.4
0.2
−log10(p−value)
15
10
5
0
100
80
60
40
20
0
Recombination rate (cM/Mb)
ADAM22→
ZNF804B→
←SRI
←C7orf62
LOC102723885→
←STEAP4
88
88.2
88.4
88.6
88.8
89
Position on chr7 (Mb)

date:	Mon Nov 21 15:13:36 2016
build:	hg19
display range:	chr7:87810316−89060427 [87810316−89060427]
hilite range:	0 – 0 [0 – 0]
reference SNP:	chr7:88359060
number of SNPs plotted:	3428
min P−value:	1.86E−15 [chr7:88359060]
max P−value:	10E−1 [chr7:88039893]

Make more plots at http://csg.sph.umich.edu/locuszoom/

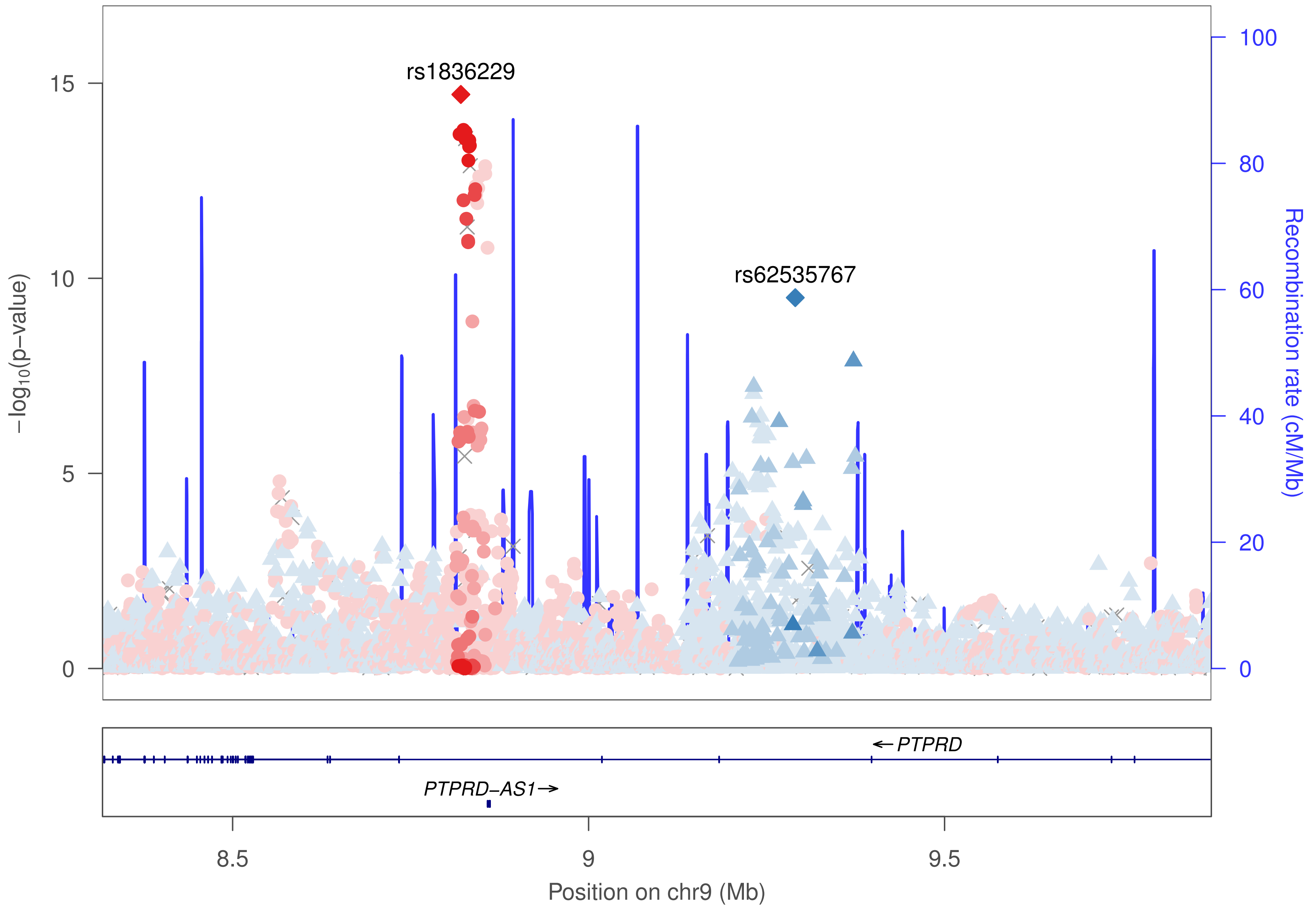

rs1836229
rs62535767
−log10(p−value)
Recombination rate (cM/Mb)
0
5
10
15
0
20
40
60
80
100
← PTPRD
PTPRD−AS1→
8.5
9
9.5
Position on chr9 (Mb)

date:	Mon Nov 21 15:15:34 2016
build:	hg19
display range:	chr9:8317244−9874664 [8317244−9874664]
hilite range:	0 – 0 [0 – 0]
reference SNP:	chr9:8820573
number of SNPs plotted:	5959
min P−value:	1.94E−15 [chr9:8820573]
max P−value:	9.99E−1 [chr9:8971336]

Make more plots at http://csg.sph.umich.edu/locuszoom/

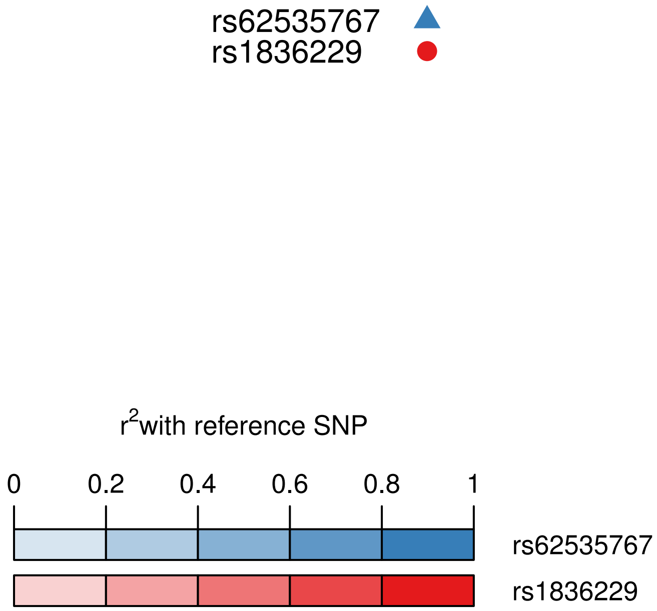
rs62535767
rs1836229
r2 with reference SNP
0
0.2
0.4
0.6
0.8
1
rs62535767
rs1836229

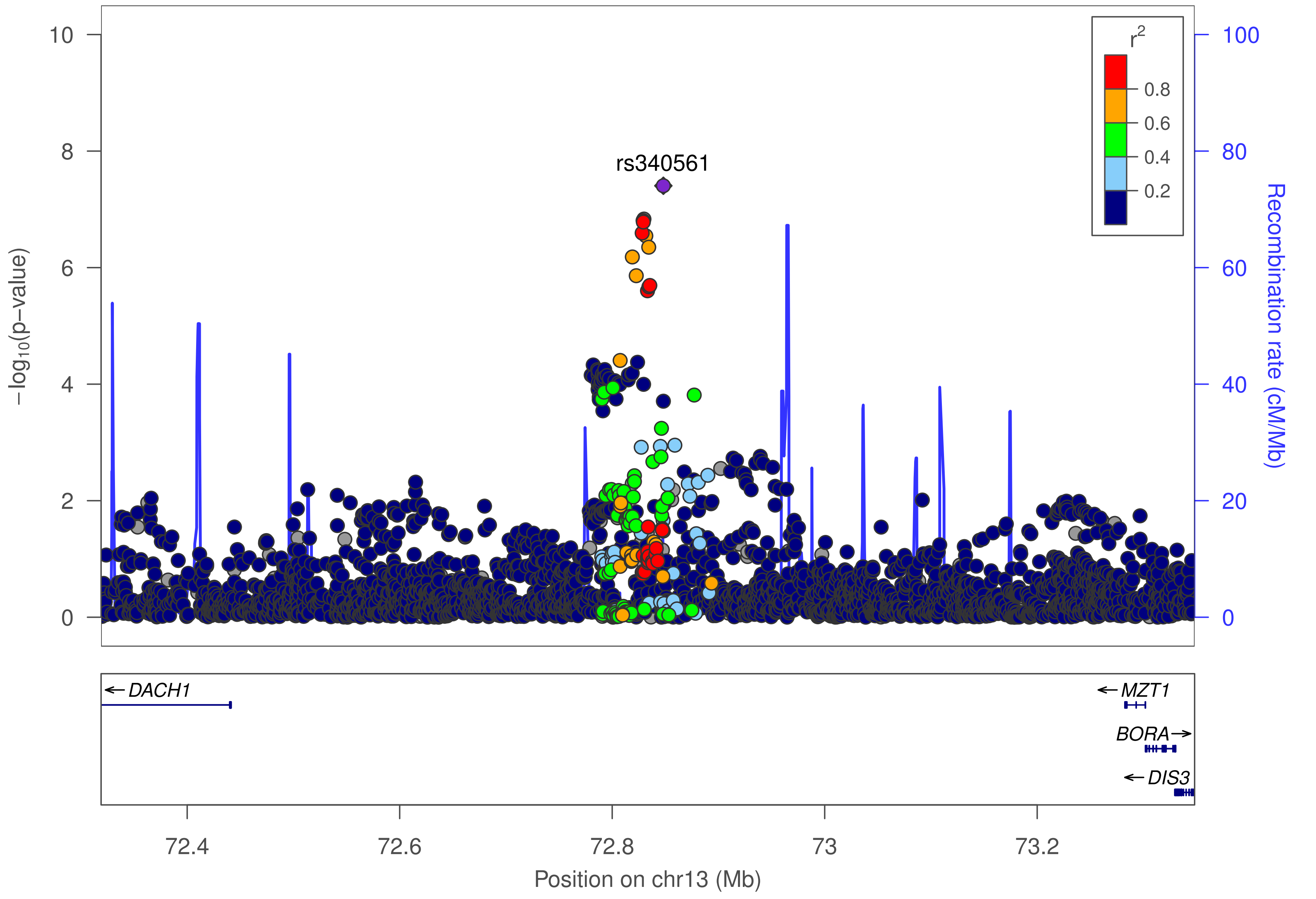

rs340561
r2
0.8
0.6
0.4
0.2
−log10(p−value)
10
8
6
4
2
0
Recombination rate (cM/Mb)
100
80
60
40
20
0
← DACH1
← MZT1
BORA →
← DIS3
72.4
72.6
72.8
73
73.2
Position on chr13 (Mb)

date:	Mon Nov 21 15:16:03 2016
build:	hg19
display range:	chr13:72318973−73348156 [72318973−73348156]
hilite range:	0 – 0 [0 – 0]
reference SNP:	chr13:72848156
number of SNPs plotted:	3067
min P−value:	3.94E−8 [chr13:72848156]
max P−value:	9.99E−1 [chr13:73178387]

Make more plots at http://csg.sph.umich.edu/locuszoom/

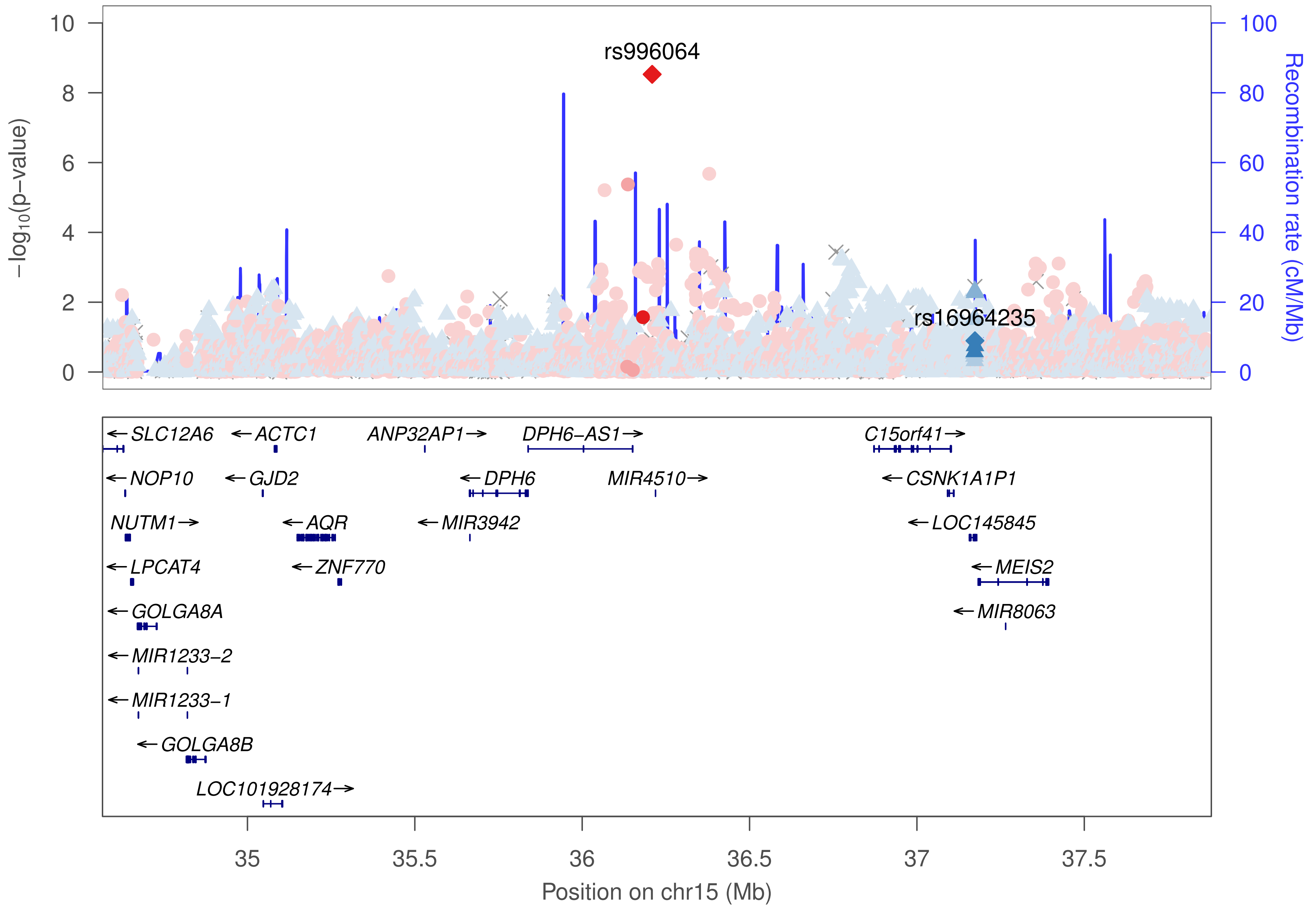

rs996064
rs16964235
−log10(p−value)
Recombination rate (cM/Mb)
0
2
4
6
8
10
0
20
40
60
80
100
SLC12A6
ACTC1
ANP32AP1
DPH6−AS1
C15orf41
NOP10
GJD2
DPH6
MIR4510
CSNK1A1P1
NUTM1
AQR
MIR3942
LOC145845
LPCAT4
ZNF770
MEIS2
GOLGA8A
MIR8063
MIR1233−2
MIR1233−1
GOLGA8B
LOC101928174
35
35.5
36
36.5
37
37.5
Position on chr15 (Mb)

date:	Thu Dec 15 14:53:40 2016
build:	hg19
display range:	chr15:34566927−37879533 [34566927−37879533]
hilite range:	0 – 0 [0 – 0]
reference SNP:	chr15:36208998
number of SNPs plotted:	8242
min P−value:	2.97E−9 [chr15:36208998]
max P−value:	1E0 [chr15:34878102]

Make more plots at http://csg.sph.umich.edu/locuszoom/

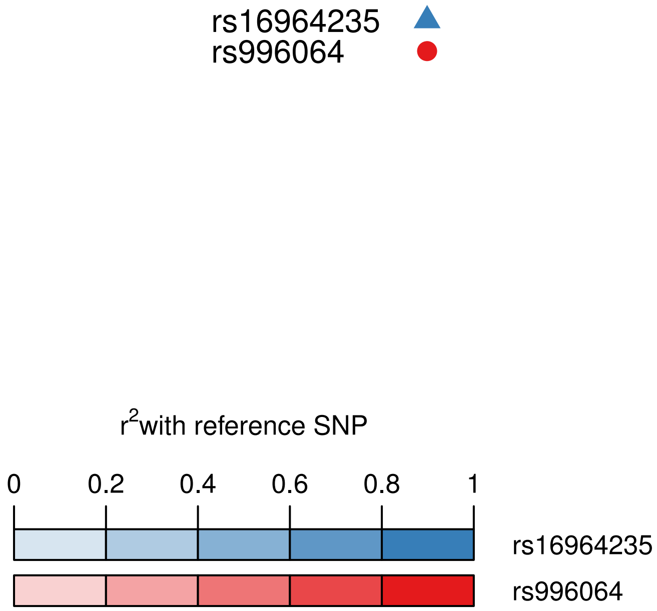
rs16964235
rs996064
r^2 with reference SNP
0
0.2
0.4
0.6
0.8
1
rs16964235
rs996064

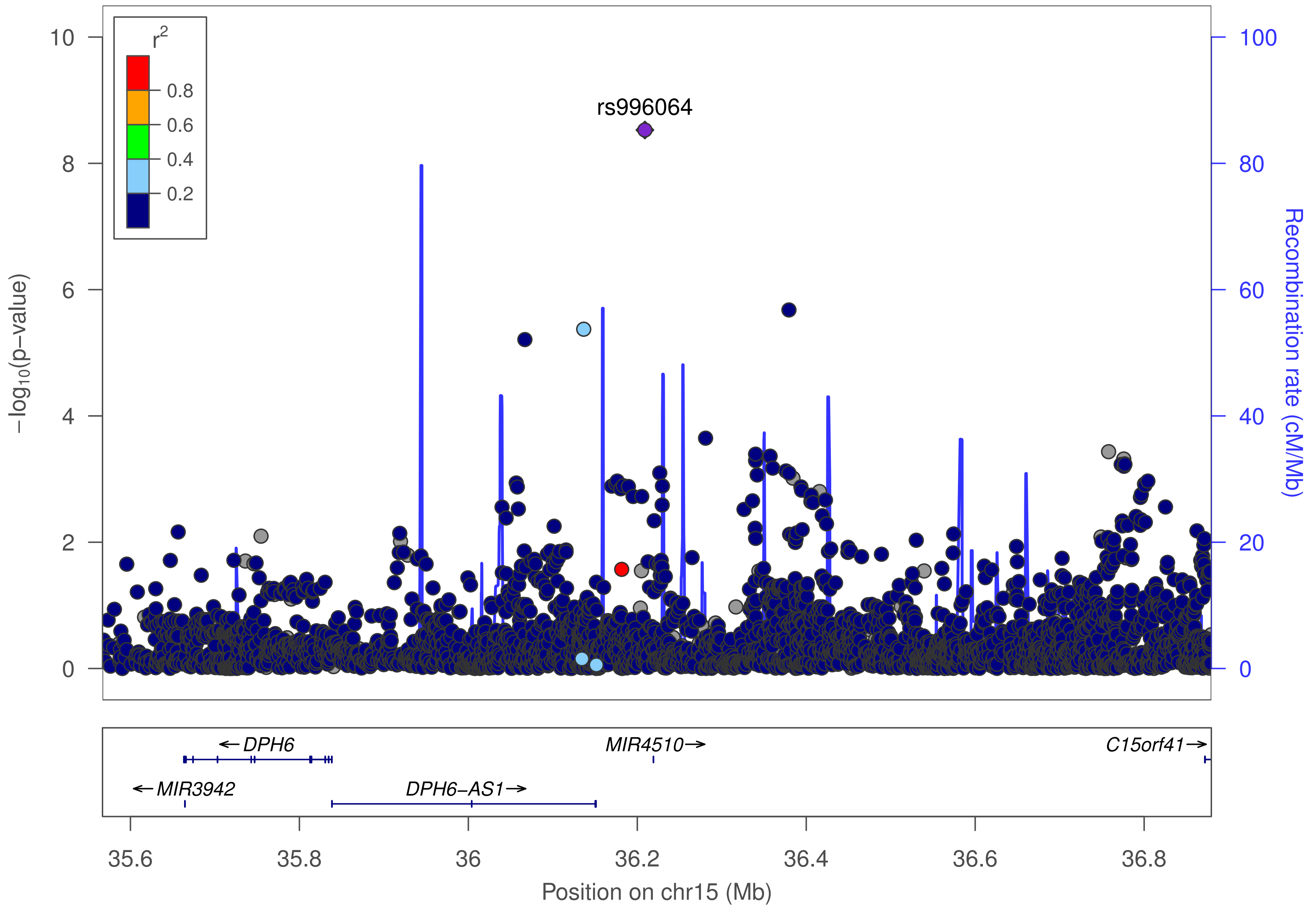

rs996064
r2
0.8
0.6
0.4
0.2
−log10(p−value)
0
2
4
6
8
10
Recombination rate (cM/Mb)
0
20
40
60
80
100
←DPH6
MIR4510→
C15orf41→
←MIR3942
DPH6−AS1→
35.6
35.8
36
36.2
36.4
36.6
36.8
Position on chr15 (Mb)

date:	Mon Nov 21 15:16:34 2016
build:	hg19
display range:	chr15:35566927−36879533 [35566927−36879533]
hilite range:	0 – 0 [0 – 0]
reference SNP:	chr15:36208998
number of SNPs plotted:	3321
min P−value:	2.97E−9 [chr15:36208998]
max P−value:	10E−1 [chr15:36122215]

Make more plots at http://csg.sph.umich.edu/locuszoom/

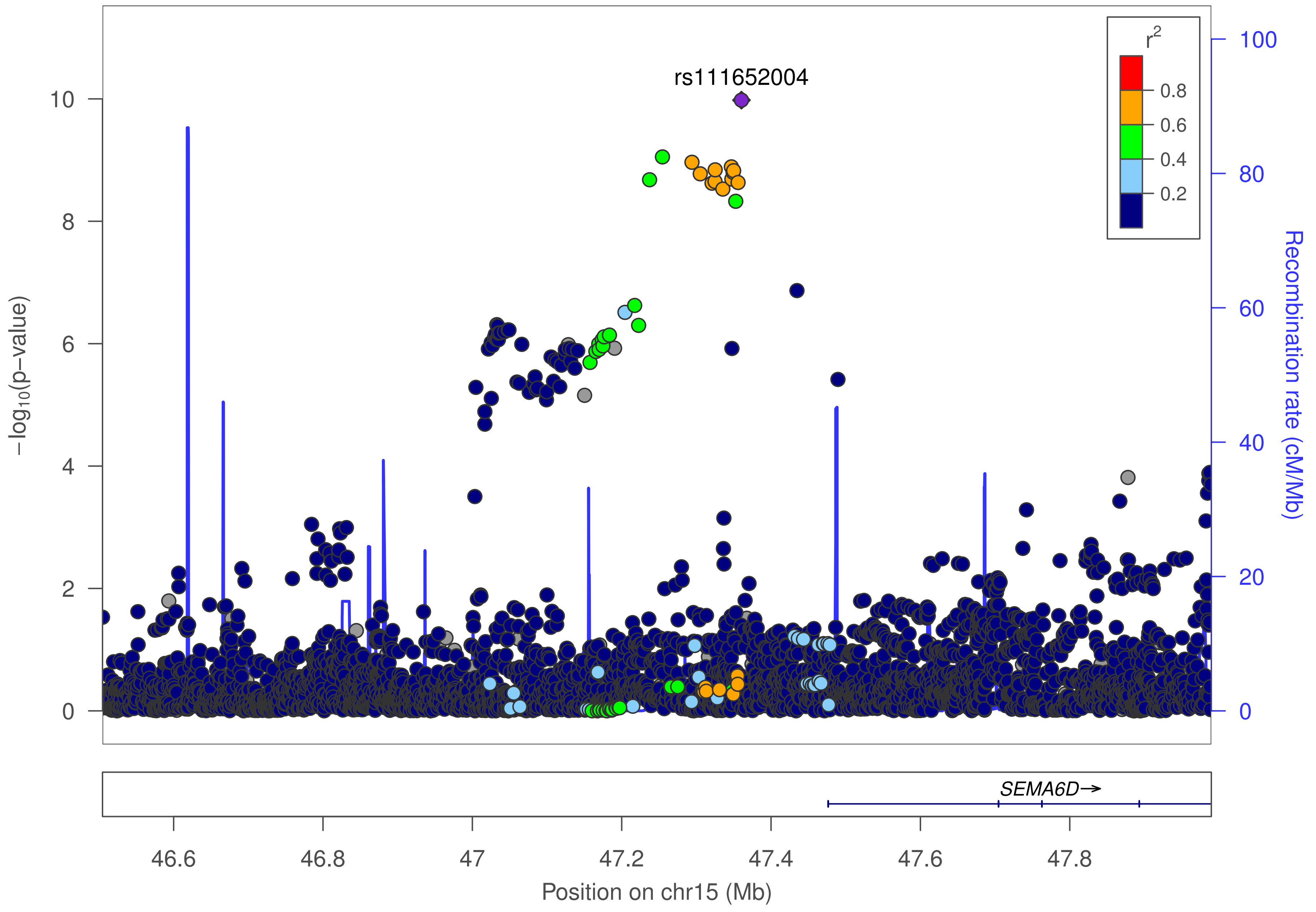
rs111652004
r2
0.8
0.6
0.4
0.2
−log10(p−value)
Recombination rate (cM/Mb)
SEMA6D→
Position on chr15 (Mb)
46.6
46.8
47
47.2
47.4
47.6
47.8
0
2
4
6
8
10
20
40
60
80
100

date:	Mon Nov 21 15:17:05 2016
build:	hg19
display range:	chr15:46504724−47989575 [46504724−47989575]
hilite range:	0 – 0 [0 – 0]
reference SNP:	chr15:47360367
number of SNPs plotted:	4069
min P−value:	1.05E−10 [chr15:47360367]
max P−value:	1E0 [chr15:47894536]

Make more plots at http://csg.sph.umich.edu/locuszoom/

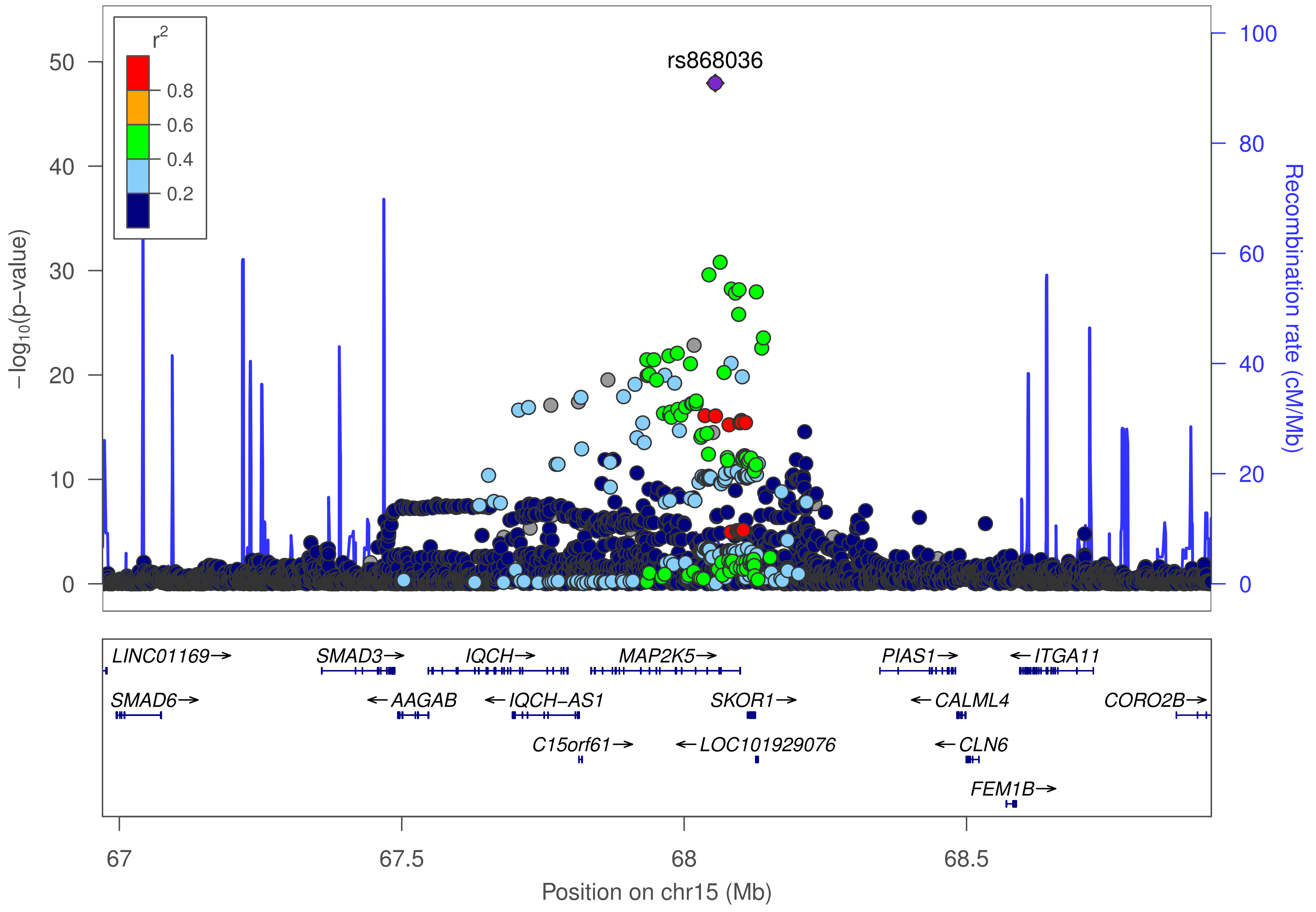

r2
0.8
0.6
0.4
0.2
rs868036
−log10(p−value)
Recombination rate (cM/Mb)
LINC01169→
SMAD3→
IQCH→
MAP2K5→
PIAS1→
←ITGA11
SMAD6→
←AAGAB
←IQCH−AS1
SKOR1→
←CALML4
CORO2B→
C15orf61→
←LOC101929076
←CLN6
FEM1B→
67
67.5
68
68.5
Position on chr15 (Mb)

date:	Mon Nov 21 15:17:39 2016
build:	hg19
display range:	chr15:66970208−68933340 [66970208−68933340]
hilite range:	0 – 0 [0 – 0]
reference SNP:	chr15:68055013
number of SNPs plotted:	4632
min P−value:	1.11E−48 [chr15:68055013]
max P−value:	10E−1 [chr15:68885374]

Make more plots at http://csg.sph.umich.edu/locuszoom/

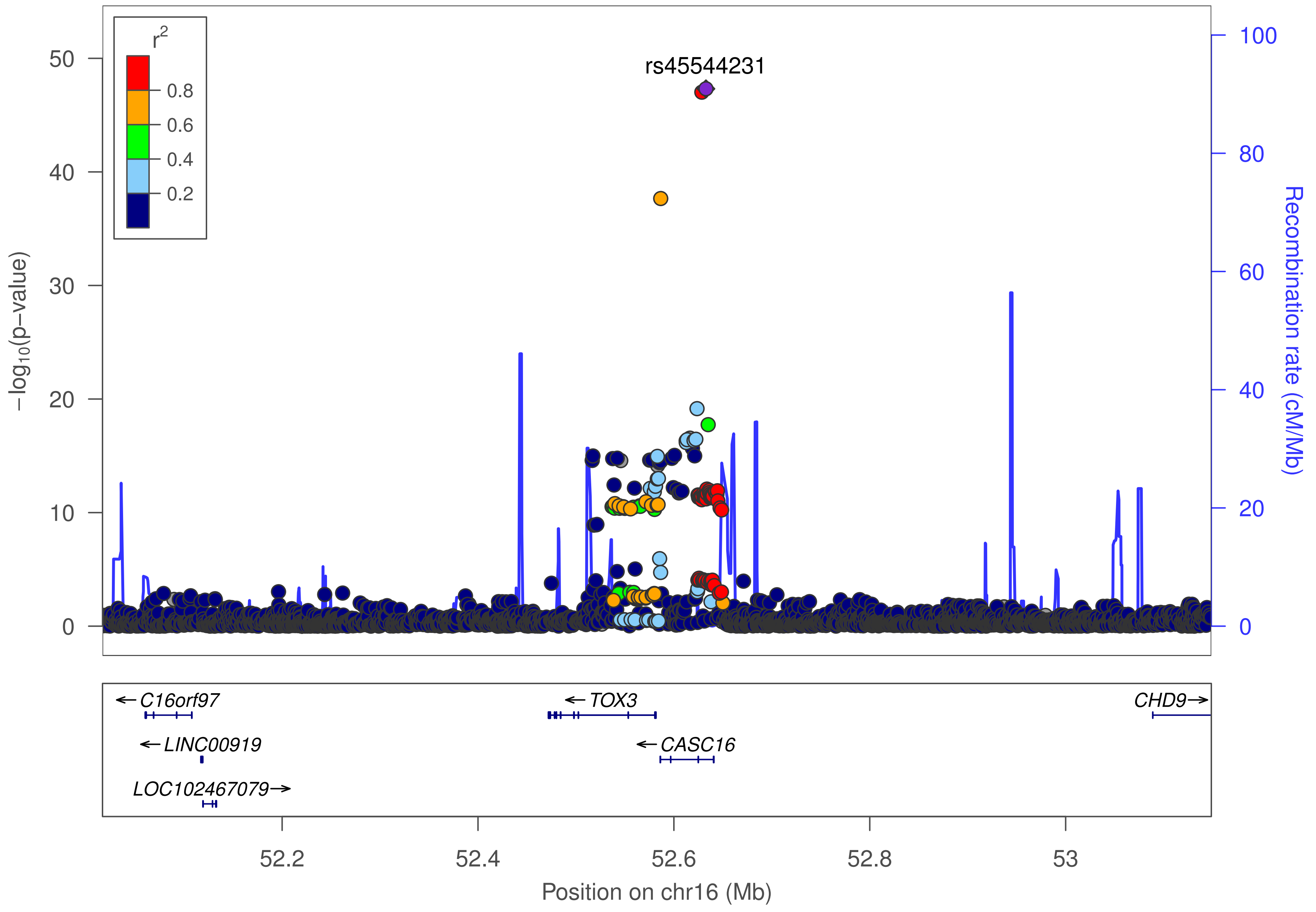

rs45544231
r2
0.8
0.6
0.4
0.2
−log10(p−value)
Recombination rate (cM/Mb)
0
10
20
30
40
50
0
20
40
60
80
100
C16orf97
LINC00919
LOC102467079
TOX3
CASC16
CHD9
52.2
52.4
52.6
52.8
53
Position on chr16 (Mb)

date:	Mon Nov 21 15:18:06 2016
build:	hg19
display range:	chr16:52016607−53148731 [52016607−53148731]
hilite range:	0 – 0 [0 – 0]
reference SNP:	chr16:52632730
number of SNPs plotted:	2839
min P−value:	4.78E−48 [chr16:52632730]
max P−value:	10E−1 [chr16:52074842]

Make more plots at http://csg.sph.umich.edu/locuszoom/

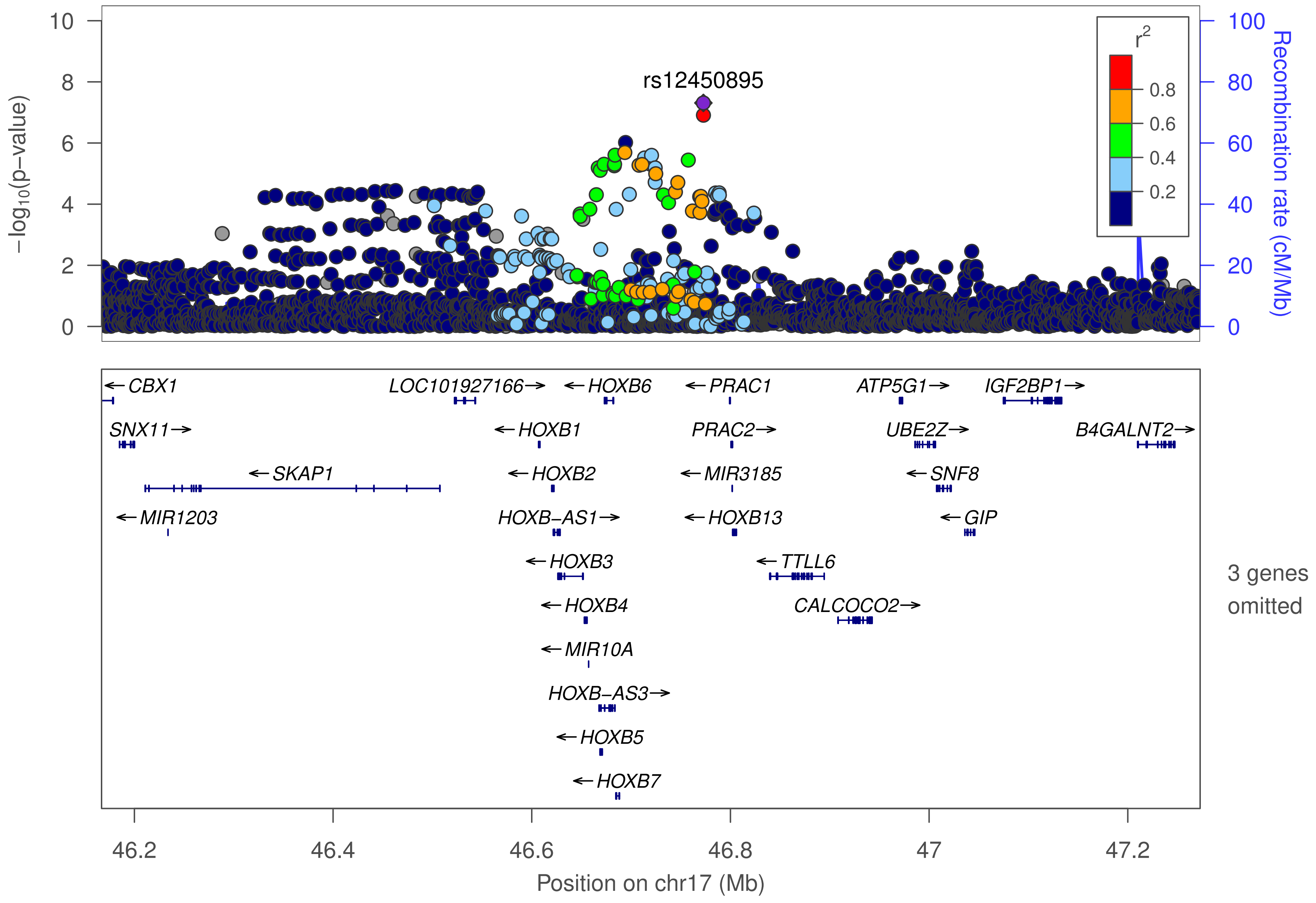
rs12450895
r2
0.8
0.6
0.4
0.2
−log10(p−value)
Recombination rate (cM/Mb)
CBX1
LOC101927166
HOXB6
PRAC1
ATP5G1
IGF2BP1
SNX11
HOXB1
PRAC2
UBE2Z
B4GALNT2
SKAP1
HOXB2
MIR3185
SNF8
MIR1203
HOXB−AS1
HOXB13
GIP
HOXB3
TTLL6
3 genes omitted
HOXB4
CALCOCO2
MIR10A
HOXB−AS3
HOXB5
HOXB7
Position on chr17 (Mb)

date:	Mon Nov 21 15:18:32 2016
build:	hg19
display range:	chr17:46166937−47272821 [46166937−47272821]
hilite range:	0 – 0 [0 – 0]
reference SNP:	chr17:46772776
number of SNPs plotted:	2734
min P−value:	4.88E−8 [chr17:46772776]
max P−value:	10E−1 [chr17:46937322]
omitted Genes:	HOXB8, HOXB9, MIR196A1

Make more plots at http://csg.sph.umich.edu/locuszoom/

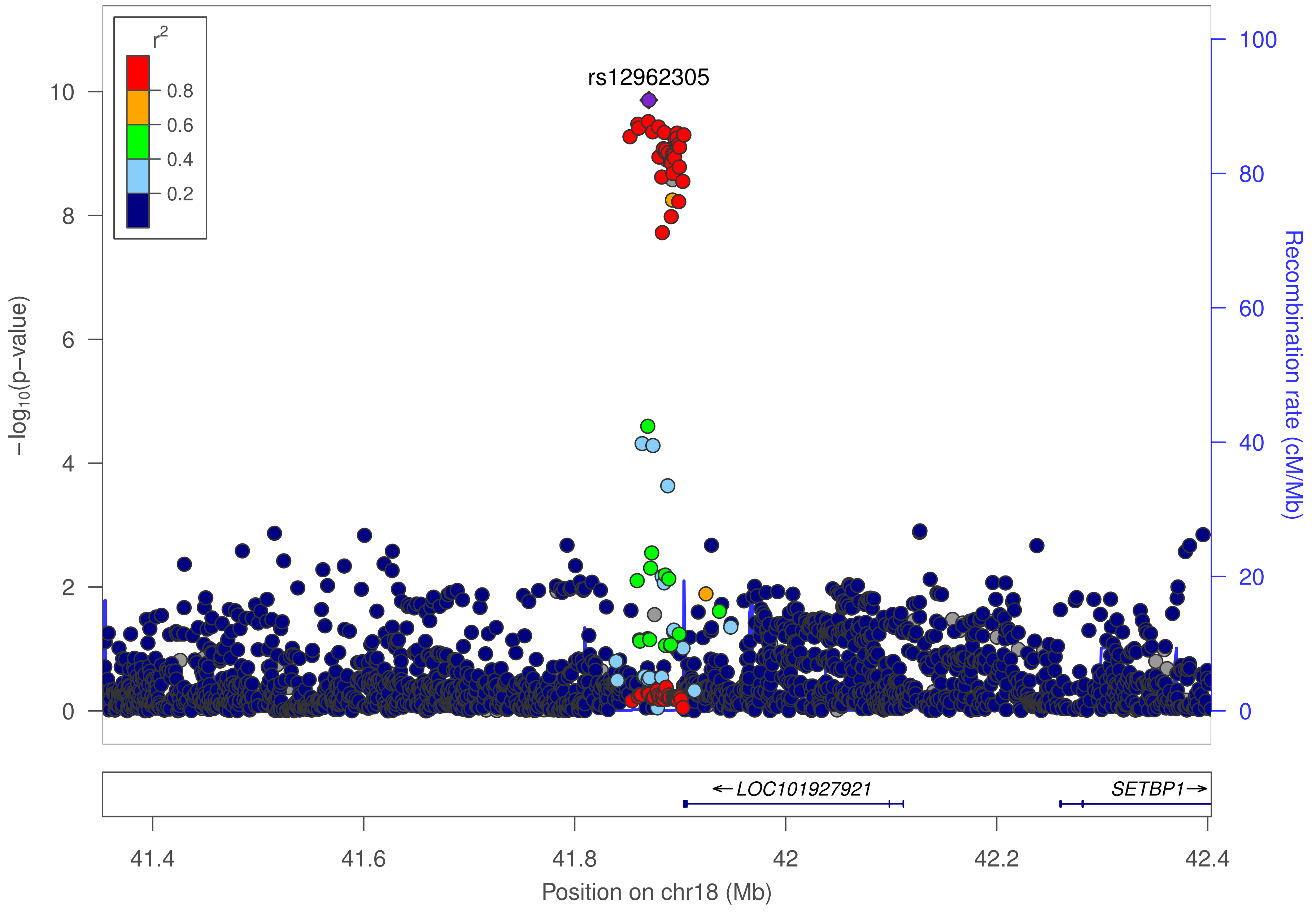

rs12962305
r2
0.8
0.6
0.4
0.2
−log10(p−value)
Recombination rate (cM/Mb)
0
2
4
6
8
10
0
20
40
60
80
100
←LOC101927921
SETBP1→
41.4
41.6
41.8
42
42.2
42.4
Position on chr18 (Mb)

date:	Mon Nov 21 15:18:56 2016
build:	hg19
display range:	chr18:41352436−42403433 [41352436−42403433]
hilite range:	0 – 0 [0 – 0]
reference SNP:	chr18:41870243
number of SNPs plotted:	2351
min P−value:	1.37E−10 [chr18:41870243]
max P−value:	9.99E−1 [chr18:41659547]

Make more plots at http://csg.sph.umich.edu/locuszoom/

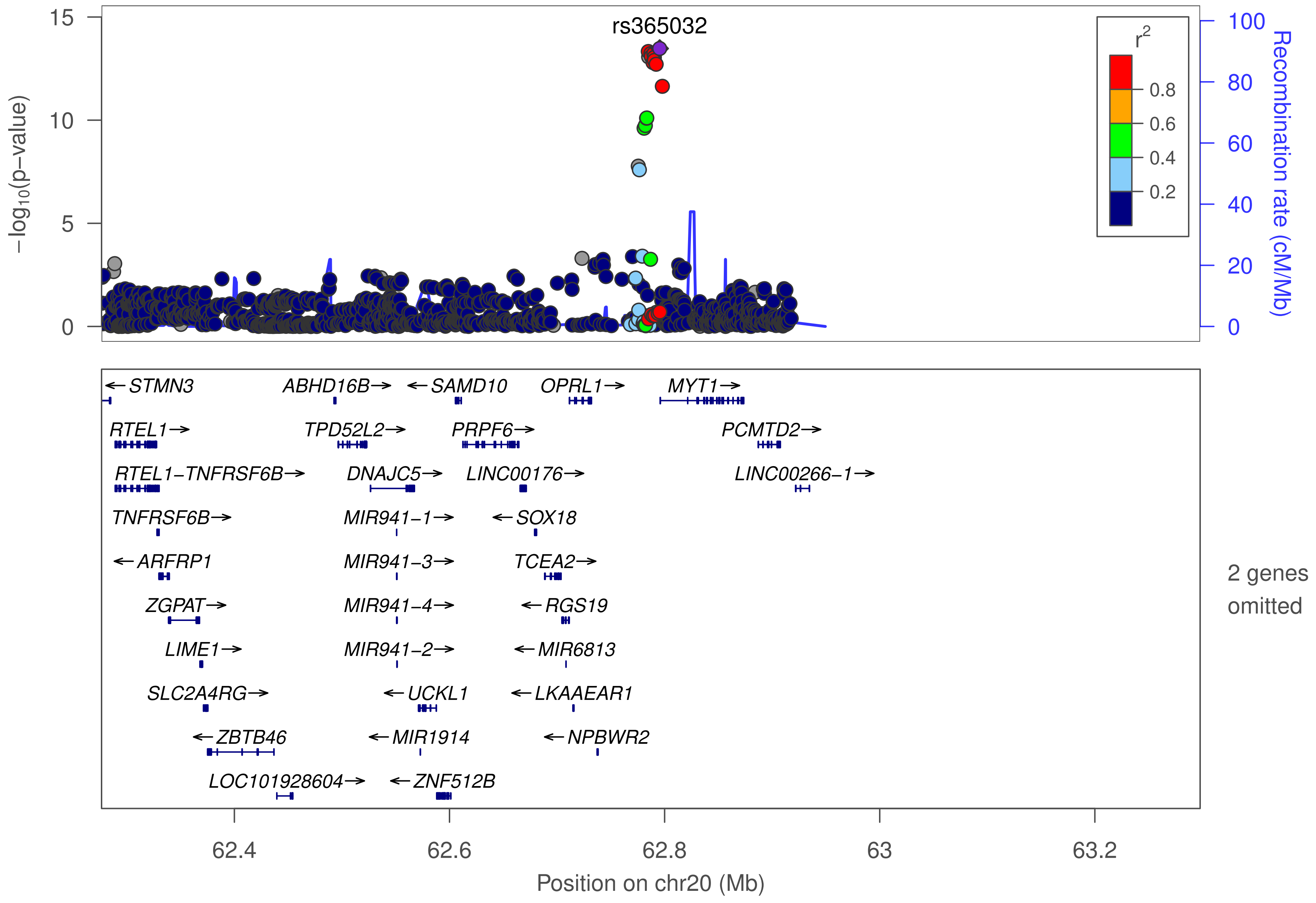
rs365032
−log10(p−value)
Recombination rate (cM/Mb)
r2
0.8
0.6
0.4
0.2
STMN3
RTEL1
RTEL1−TNFRSF6B
TNFRSF6B
ARFRP1
ZGPAT
LIME1
SLC2A4RG
ZBTB46
LOC101928604
ABHD16B
TPD52L2
DNAJC5
MIR941−1
MIR941−3
MIR941−4
MIR941−2
UCKL1
MIR1914
ZNF512B
SAMD10
PRPF6
LINC00176
SOX18
TCEA2
RGS19
MIR6813
LKAAEAR1
NPBWR2
OPRL1
MYT1
PCMTD2
LINC00266−1
2 genes omitted
Position on chr20 (Mb)

date:	Mon Nov 21 15:19:19 2016
build:	hg19
display range:	chr20:62276506−63297873 [62276506−63297873]
hilite range:	0 – 0 [0 – 0]
reference SNP:	chr20:62795405
number of SNPs plotted:	1207
min P−value:	3.36E−14 [chr20:62795405]
max P−value:	9.99E−1 [chr20:62552515]
omitted Genes:	MIR647, UCKL1−AS1

Make more plots at http://csg.sph.umich.edu/locuszoom/
